# Author Correction: From coarse to fine: the absolute *Escherichia coli* proteome under diverse growth conditions

**DOI:** 10.1038/s44320-024-00062-5

**Published:** 2024-10-01

**Authors:** Matteo Mori, Zhongge Zhang, Amir Banaei-Esfahani, Jean-Benoît Lalanne, Hiroyuki Okano, Ben C Collins, Alexander Schmidt, Olga T Schubert, Deok-Sun Lee, Gene-Wei Li, Ruedi Aebersold, Terence Hwa, Christina Ludwig

**Affiliations:** 1 Department of Physics, University of California at San Diego, La Jolla, California USA; 2 Section of Molecular Biology, Division of Biological Sciences, University of California at San Diego, La Jolla, California USA; 3 Department of Biology, Institute of Molecular Systems Biology, ETH Zurich, Zurich, Switzerland; 4 Department of Biology, Massachusetts Institute of Technology, Cambridge, USA; 5 Department of Physics, Massachusetts Institute of Technology, Cambridge, USA; 6 School of Biological Sciences, Queen’s University of Belfast, Belfast, UK; 7 Biozentrum, University of Basel, Basel, Switzerland; 8 Department of Human Genetics, University of California, Los Angeles, Los Angeles, CA USA; 9 Department of Physics, Inha University, Incheon, Korea; 10 Faculty of Science, University of Zurich,, Zurich, Switzerland; 11 Institute for Theoretical Studies, ETH Zürich, Zürich, Switzerland; 12 Bavarian Center for Biomolecular Mass Spectrometry (BayBioMS), Technical University of Munich (TUM), Freising, Germany

## Abstract

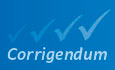

**Correction to:** *Molecular Systems Biology* (2021) 17:e9536. 10.15252/msb.20209536 | Published online 25 May 2021

The authors contacted the journal after being made aware of errors in the manuscript. Based on the exchanges with the authors, the journal has agreed to withdraw and replace the following figures and datasets.

**Figure panels 4D, 4E, 4I and 4J are withdrawn and replaced**.

**Datasets EV6, EV7, EV8, EV9 and EV11 are corrected.**

Author statement:

Figure 4:

In Figure 4, the labelling of Panel 4D and Panel 4E (A-sector and S-sector) has been mistakenly inverted, as well as the indication of regulations. Additionally, in Figure 4I and 4J, the indication of regulation has been inverted (while here, the sector name is correct).

**Figure 4:**
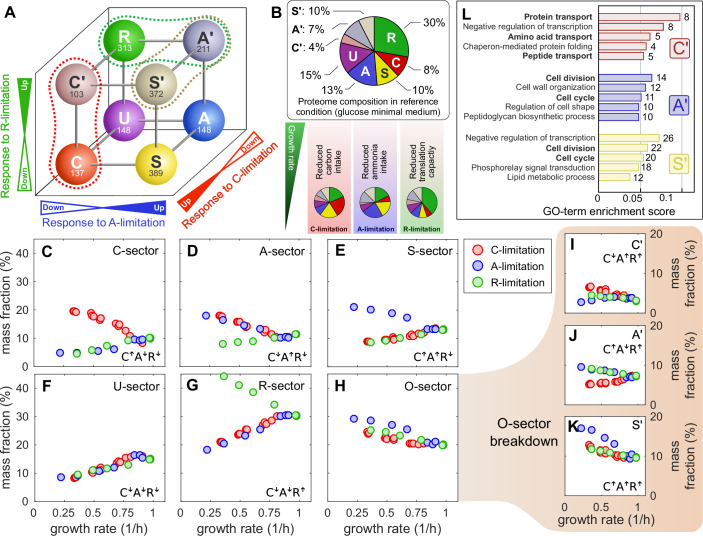
**Original**.

**Figure 4:**
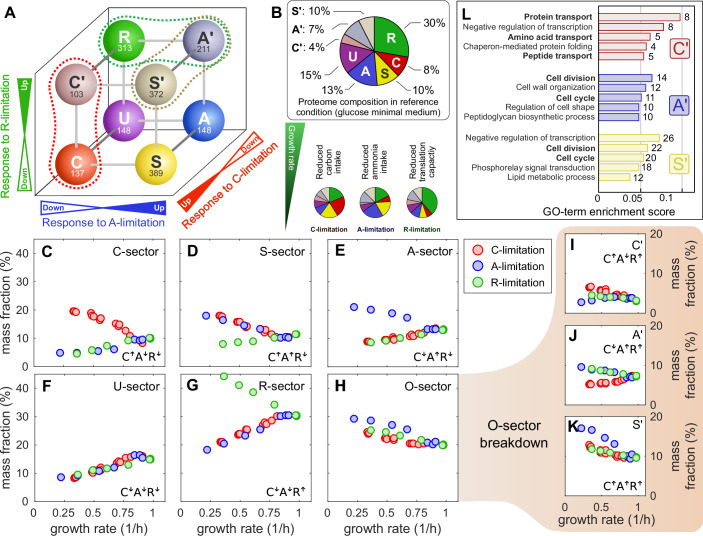
**Corrected**.

Datasets:

It was brought to our attention that the protein molecular weights reported in Dataset EV6 appeared incorrect. We found that these were mistakenly inflated by about 20% due to a bioinformatic error. We corrected the molecular weights and recalculated the protein mass fractions in datasets EV6, EV7, EV8 and EV9, as well as the slopes in dataset EV11. The maximum change in the protein mass fractions compared to the ones reported in the retracted datasets is less than 1%, and the classification of proteins into protein sectors is not affected. Thus, this error does not affect in any way the conclusions of the original paper.

All authors agree to this correction and apologize to the readers for this error.

## Supplementary information

EV6 corrected

EV7 corrected

EV8 corrected

EV9 corrected

EV11 corrected

