# Supplementary material for: From coarse to fine: the absolute Escherichia coli proteome under diverse growth conditions
Source: Mol Syst Biol. 2021 May 25;17(5):e9536. doi: 10.15252/msb.20209536 (PMC8144880; doi:10.15252/msb.20209536)
Supplement: Supplementary file 1 — Appendix [file MSB-17-e9536-s013.docx]

From coarse to fine: The absolute *Escherichia coli* proteome under diverse growth conditions

Appendix

Matteo Mori, Zhongge Zhang, Amir Banaei Esfahani, Jean-Benoît Lalanne, Hiroyuki Okano, Ben C. Collins, Alexander Schmidt, Olga T. Schubert, Deok-Sun Lee, Gene Wei Li, Ruedi Aebersold^†^, Terence Hwa^†^, Christina Ludwig^†^

Table of contents

[Extended experimental methods 2](#_Toc70433784)

[Note S1: Absolute protein mass fractions and concentrations 14](#_Toc70433785)

[Note S2: xTop protein intensities 19](#_Toc70433786)

[Note S3: Quantitative analysis of bias in protein intensities 30](#_Toc70433787)

[Note S4: GO-term enrichment analysis 36](#_Toc70433788)

[Appendix Figures 37](#_Toc70433789)

[Datasets 52](#_Toc70433790)

[References 53](#_Toc70433791)

# Extended experimental methods

## Strains

Most of this work is based on *E. coli* K-12 strain NCM3722 (Brown & Jun, 2015) whose growth physiology has been extensively characterized (Hui *et al*, 2015; You *et al*, 2013). A few derivatives of NCM3722 were also used: NQ1243 and NQ1390, which allow to titrate the glucose intake flux (Basan *et al*, 2015a); NQ393, which expresses GOGAT from a titratable promoter in GDH-null background (Hui *et al.*, 2015); EQ59, a non-motile strain which constitutively expresses GFP from the chromosome (Warren *et al*, 2019); NQ1431, which harbors *phnE^+^* allele and can utilize phosphonate as a sole phosphorus source (Chen *et al*, 1990); NQ1527, which restores a mutation in the *rpoS* gene. Construction of strains NQ1390, NQ1431 and NQ1527 are described below. For the calibration samples A1, C1 and F1, we used strain EQ353, which is the specific MG1655 strain used in Li et al. (Li *et al*, 2014) 2014. Additionally, we used *E. coli* K-12 strain MG1655 obtained from the Coli Genetic Stock Center (CGSC#6300) and *E. coli* Nissle1917 isolated from a Mutaflor capsule (Pharam-Zentrale, Germany).

**Construction of strain NQ1390 (samples C6, C7, D7, D8, F7, F8):** Similarly to the previously characterized strain NQ1243 (Basan *et al.*, 2015a), this strain allows titration of the glucose transporter PtsG, a subunit of the glucose PTS permease. This is done by replacing the *ptsG* promoter with a titratable *Pu* promoter from *Pseudomonas putida*; the activity of the *Pu* promoter is regulated by *xylR* upon induction by 3MBA. Compared to NQ1243, strain NQ1390 differs in the promoter driving the *xylR* gene: expression of *xylR* is driven by a *lacIq* promoter in strain NQ1390, as opposed to a *Ptet* promoter in NQ1243. This allows to titrate PtsG (and hence the growth rate) to lower levels in NQ1390 compared to NQ1243.

**Construction of strain NQ1431 (Lib-09):** In *E. coli* K-12, the phosphonate transport system is cryptic due to an 8 bp insertion in *phnE* gene (Makino *et al*, 1991) while it is functional in *E. coli* B (Chen *et al.*, 1990) such as BL21 (DE3) strain (Novagen). Accordingly, NCM3722, a derivative of K-12, cannot grow on minimal medium supplemented with phosphonate as a sole phosphorus source. To render NCM3722 the ability to utilize phosphonate as a sole phosphorus source, we transferred *phnE^+^* allele from BL21 (DE3) strain into NCM3722 by P1 transduction using phage P1*vir* and selected a transductant which grew on minimal glucose agar medium supplemented with 2-amino-ethylphosphonate as a sole phosphorus source. This strain is designated as NQ1431.

**Construction of strain NQ1527 (Lib-13):** NCM3722 carries an amber nonsense mutation (TAG) at codon 33 of *rpoS* gene (Brown & Jun, 2015; Lyons *et al*, 2011a) while it is mutated to CAG encoding glutamine in MG1655. To “fix” this amber mutation by CAG codon in MG1655, we conducted two-step P1 transduction mediated by phage P1*vir*, taking advantage of the phenotype of the null mutation of *cysC* (i.e. the inability to grow in minimal glucose medium), which is located close to *rpoS* in the chromosome. First, ∆*cysC752*::*kan* allele of strain JW2720-1 from Keio collection (CGSC#: 10139) (Baba *et al*, 2006) was transferred into NCM3722 by P1 transduction. The resulting Kan^r^ strain has a deletion in *cysC* and cannot grow in glucose minimum medium. Next, this strain was transduced with a new phage P1*vir* prepared from MG1655 and the transductants were selected on a minimum glucose agar medium. One of the clones was confirmed to carry CAG at codon 33 of *rpoS* by sequencing, yielding the strain NQ1527.

## Growth media

Unless otherwise indicated, growth media used are based on one of the following base media: modified Record’s MOPS medium (Chen *et al.*, 1990), phosphate-buffered “N^-^C^-^” medium (Csonka *et al*, 1994; Gutnick *et al*, 1969), M9 medium (Kochanowski *et al*, 2013), and Luria-Bertani (LB) medium. Other ingredients such as carbon and nitrogen sources were supplied as described in Datasets EV2 and EV3.

**Standard procedure of growth experiments**

*E. coli* cells collected for the library samples were grown in batch culture as described below. Batch cultures were grown in a 37°C water bath shaker shaking at 250 rpm for aeration. Each growth experiment was carried out in three steps: seed culture in LB broth, pre-culture and experimental culture in an identical growth medium. For seed culture, cells from a single colony grown on LB agar plate was inoculated into liquid LB and grown at 37°C with shaking. Cells in the seed culture were then transferred into the growth medium with proper dilution and grown at 37°C overnight (pre-culture). Cells from the overnight pre-culture was then transferred into the growth medium with proper dilution, and grown at 37°C until harvested for proteomic analysis as described below. Optical densities at 600 nm were measured with a spectrophotometer Genesys 20 (Thermo Scientific).

The strain and the growth condition used for each sample are described in Datasets EV1 – EV3. Below we describe the specific procedure of the growth experiment for each sample.

**Steady state culture with growth limited by carbon uptake (Lib-00, Lib-06, Lib-13, Lib-24 to Lib-30; A1, A2, C1-C8, D6-D8, F1, F4-F8, H1, H5)**.

The growth experiments were carried out according to the standard procedure with strains and growth media described in Datasets EV1-EV3. For the A1, C1 and F1 samples, both strain, growth media and experimental procedures are identical to those used in a ribosome profiling study (Li *et al.*, 2014) to measure protein synthesis rates for *E. coli* in glucose-limited media.

**Steady state culture with growth limited by N or P (Lib-08, Lib-09, Lib-15; D1-D5, F2, F3).**

For **Lib-15**, the growth experiments were carried out according to the standard procedure with aspartate instead of NH_4_Cl as a sole nitrogen source.

For **Lib-09**, NQ1431 (*phnE*^+^) cells were grown according to the standard procedure with 1.32 mM KCl added instead of 1.32 mM KH_2_PO_4_ and 0.4 mM 2-amino-ethylphosphonate (Sigma) supplied as a phosphorus source.

For **Lib-08, D1-D5, F2** and **F3**, the pre-culture of NQ393 (an NCM3722 derivative with low GOGAT expression in GDH-null background (Hui *et al.*, 2015) was grown in M9 medium supplemented with 40 mM glycerol, 2 mM glutamate, and the same concentration of IPTG as in the growth medium or 30 μM IPTG if IPTG concentration in the growth medium is below 30 μM. The overnight pre-culture was then inoculated into M9 medium supplemented with 0.2% glucose and 20 μM IPTG.

**Steady state culture under anaerobic growth (Lib-05).**

The medium used for the anaerobic growth of *E. coli* NCM3722 consisted of 39 mM KH_2_PO_4_, 61 mM K_2_HPO_4_, 100 mM NaCl, 20 mM NH_4_Cl, 20 mM glucose, 0.4 mM MgCl_2_, 0.5 mM CaCl_2_, 4 μM FeSO_4_, 50 μM MnCl_2_, 50 μM CoCl_2_, 20 mM NaHCO_3_, 5 mM cysteine, 2.5 mg/l vitamin B_12_, and 2 mg/l hemin.

Anaerobic batch culture growth was performed similarly as aerobic growth with a number of exceptions: Seed culture of NCM3722 cells was grown aerobically in LB broth and passed into an anaerobic chamber (Coy Laboratory Products) through an airlock. Inside the anaerobic chamber, 100 μl of the seed culture was diluted into 3 ml growth medium in 16 mm glass tubes and grown as a pre-culture in a dry bath (Eppendorf ThermoMixer C with a 15ml Thermoblock) at 37°C shaking at 500 rpm. At OD_600_ = 0.2 - 0.3, the pre-culture was diluted into the growth medium to OD_600_ ≈ 0.02 and grown until harvested for proteomic analysis.

**Steady state culture grown in various stress conditions (Lib-01 to Lib-04, Lib-07, Lib-17, Lib-18, Lib-31, Lib-32, E1-E4).**

**Heat stress (Lib-01):** The pre-culture of NCM3722 cells was grown at 37°C according to the standard procedure, then diluted into the same medium and shifted to 42°C. Samples were taken for mass spec analysis after the culture had adapted to the exponential growth phase.

**Hyperosmolarity stress (Lib-02 to Lib-04, Lib-31, Lib-32)**, NCM3722 cells were grown according to the standard procedure except that the seed cultures were grown in LB supplemented with 0.3 M NaCl.

**Acetic acid stress:** For **Lib-17**, EQ59 cells (an NCM3722 derivative expressing GFP) were first grown in LB with 50 mM acetic acid, and then grown in the growth medium supplemented with 50mM acetic acid.

**Ethanol stress:** For **Lib-18**, EQ59 cells (an NCM3722 derivative expressing GFP) were first grown in LB with 500 mM ethanol, and then grown in the growth medium supplemented with 500 mM ethanol.

**Antibiotics stress:** For **Lib-07**, NCM3722 cells were grown according to the standard procedure with 12 μg/ml kanamycin added to the pre-culture and growth culture. For samples **E1** to **E4**, NCM3722 cells were grown according to the standard procedure with chloramphenicol added to the pre-culture and growth culture.

**Colonies grown on solid substrates (Lib-10, Lib-11, Lib-16)**

For Lib-10 and Lib-16, seed culture of EQ59 cells (a non-motile NCM3722 derivative expressing GFP) grown in LB were transferred to phosphate-buffered “N^-^C^-^“ medium supplemented with 10 mM NH_4_Cl as the sole nitrogen source and 0.2% (w/v) glucose as the carbon source. The overnight pre-culture was then diluted into the same minimal medium. 10 µL of the diluted culture, containing approximately 10 cells, were deposited on a pre-heated agar plate containing the same growth medium. 1% agar was used for Lib-10 and 1.5% for Lib-16. The plates were incubated at 37°C, and a number of colonies formed and expanded on the agar surface (Warren *et al.*, 2019). The colonies were harvested 51 h after seeding for Lib-10 and 46 hours after seeding for Lib-16 for mass spectroscopy analysis. Note that EQ59 is non-biofilm forming.

For Lib-11, *E. coli* Nissle1917 was used. This strain was isolated from a Mutaflor capsule (Pharam-Zentrale, Germany) by plating on a freshly prepared LB agar plate, then storing in a glycerol-stock and kept at -80°C. Nissle1917 is capable of forming biofilms (Sonnenborn & Schulze, 2009). To induce biofilm formation, cells were grown in a minimal medium with low osmolarity. This medium is Neidhardt’s MOPS minimal medium (Neidhardt *et al*, 1974), but with ¼ of the stated MOPS buffer (10 mM final concentration); further, NaCl concentration was adjusted to a final concentration of 10 mM (instead of 50 mM). 10mM NH_4_Cl was provided as the nitrogen source, and 30mM sodium acetate was provided as carbon sources. Nissle1917 cells from seed culture in an LB medium were inoculated into the low osmolarity medium, and allowed to grow for about 24 hours at 37°C. Overnight, strong clumping of cells occurred as was clearly visible by eye. For sample collection, a 2ml of the culture was taken and vortexed ($\sim$30 sec) to (partially) dissolve the cell clumps and then processed as the other samples.

**Non-steady state culture in various conditions (Lib-12, Lib-14, Lib-19 to Lib-23)**

**Transient oxidative stress (Lib-14):** NCM3722 cells were grown according to the standard procedure. At OD_600_ = 0.58, 400 µM of hydrogen peroxide (H_2_O_2_) was added, and samples were collected 20 minutes after H_2_O_2_ addition.

**Different phases of growth on LB (Lib-19 to Lib-22):** For libraries 19-22, NCM3722 cells were grown in LB by successively diluting them twice at OD_600_ ≈ 0.3 to fresh LB medium without reaching late log or stationary phase (no overnight growth). After the second dilution, the culture was allowed to go to the stationary phase, and samples were collected along the way: at OD_600_ ≈ 0.6 (Lib-20), OD_600_ ≈ 2.5 (Lib-21), OD_600_ ≈ 3.44 (Lib-19 and Lib-22). Optical densities were determined by first diluting the culture below OD_600_ ≈ 0.4 and then multiplying back the dilution factor.

**Stationary phase following growth on LB at reduced temperature (Lib-12):** MG1655 (variant CGSC#6300 from Coli Genetic Stock Center) was first grown in LB as a seed culture at 37°C. At OD_600_ ≈ 0.5, the seed culture was diluted 1000-fold into fresh LB medium and incubated at 25°C for 24 hours (well into the stationary phase).

**Stationary phase following growth in low buffer, low osmolarity medium (Lib-23):** MG1655 cells (same as in sample Lib-12) were first grown in LB as seed culture and subsequently transferred to a low buffer, low salt medium. This medium is as described above for Lib-11, except that 20mM maltose and 20mM pyruvate were provided as the carbon source. The pre-culture grown in this medium was diluted into fresh medium and allowed to grow overnight. Note that this growth condition induces the formation of biofilm for Nissle1917 (see description below for Lib-11); however, the variant of MG1655 used here is not biofilm forming.

## Spectral library generation

### Sample preparation

The proteomic sample preparation was performed using an optimized *E. coli* protocol described previously by Schmidt *et al.* (Schmidt *et al*, 2016b). Briefly, *E. coli* cell pellets, derived from cells grown under the various growth conditions indicated above, were lysed by resuspension in lysis buffer (2% sodium deoxycholate in 100 mM ammonium bicarbonate) and two rounds of ultrasonication for 10 seconds in a vial tweeter (Hielscher). Subsequently all sample were heated for 10 minutes at 95°C, and the total protein content was determined by a bicinchoninic acid assay (BCA assay, Pierce). Cysteine residues were reduced with 5 mM Tris(2-carboxyethyl)phosphine (TCEP, Sigma) and alkylated with 10 mM iodoacetamide (Sigma). Next proteins were digested using a combinatorial LysC (Wako) and trypsin (Promega) approach: First, sodium deoxycholate concentration was lowered to 1% using 0.1 M ammonium bicarbonate buffer and then LysC was added to a final enzyme/protein ratio of 1:200 w/w and digestion was started at 37°C for 4 hours. Second, trypsin was added to a final enzyme/protein ration of 1:50 and digestion was started at 37°C over night. Finally, the resulting peptide mixtures were purified and desalted by solid-phase extraction (SEP-PAK columns, standard procedure). The purified peptides were dried to completeness with a speedvac system and resuspended in MS-buffer (2% acetonitrile, 98% water, 0.1% formic acid) to a final concentration of 0.5 or 0.66 µg/µl. Finally, the iRT peptide mix (Biognosys) was spiked into all samples at a concentration of 1:20 v/v for the purpose of retention time alignment. In each LC-MS/MS measurement 2 µg of total peptide amount (according to nanodrop measurements) was injected onto the nano-HPLC system.

Exclusively to the three biological replicates of the calibration sample (*E. coli* strain K-12 MG1655 grown in glucose minimal media at exponential growth phase) a set of 29 stable isotope labeled peptides (AQUA peptides (Gerber *et al*, 2003)) was spiked after digestion and before C18 purification. Depending on the previously determined endogenous peptide intensities, either a concentration of 10 fmol/µl or 100 fmol/µl was spiked. Those 29 isotope-labeled AQUA peptides were used to absolutely quantify 29 anchor proteins and to hereby confirm the high proteome similarity (also in absolute terms) between the calibration sample generated in-house and the sample studied and published by Li *et al.* using ribosomal profiling (Li *et al.*, 2014). The absolute quantitative analysis using AQUA peptides has been performed with the Skyline software (MacLean *et al*, 2010) and all the data has been deposited to Panorama public (Sharma *et al*, 2018).

### Off-gel electrophoresis (OGE)

To increase the number of peptides and proteins covered in the *E. coli* spectral library, a peptide fractionation procedure based on off-gel isoelectric focusing (OGE) was used, which separates peptides according to their pI, as described previously (Malmstrom *et al*, 2006; Picotti *et al*, 2009). 33 µg peptides from the sample Lib-01 to Lib-30 (Dataset EV2) were pooled and solubilized in OGE buffer (5.6 M urea, 1.6 M thiourea, 5% v/v glycerol, 1% w/v dithiothreitol (DTT), and 1% v/v carrier ampholytes mixture (IPG buffer pH 3.0-10.0, GE Healthcare). The peptides were separated on a 3100 OFFGEL Fractionator (Agilent Technologies) using an immobilized pH gradient strip of 24 cm (pH 3-10, GE Healthcare) at a maximum of 8000 V, 50 µA and 200 mW. In total 24 OGE fractions were generated, which were further pooled into 13 fractions using a previously established pooling scheme that maximizes peptide coverage and minimizes fraction numbers (F1 = OGE1, F2 = OGE2, F3 = OGE3, F4 = OGE4, F5 = OGE5, F6 = OGE6 + OGE7, F7 = OGE8 + OGE 9, F8 = OGE10 + OGE11, F9 = OGE12 + OGE13 + OGE 14 + OGE15, F10 = OGE16 + OGE17 + OGE18 + OGE 19, F11 = OGE20 + OGE 21, F12 = OGE 22, F13 = OGE23 + OGE 24). The 13 final fractions were desalted (MicroSpin columns, The Nest Group Inc.), dried to completeness in a SpeedVac system, re-suspended in 0.1 % v/v formic acid, the iRT peptide mix (Biognoysis) added and analyzed individually by DDA-based LC-MS/MS.

### DDA mass spectrometry

All 53 LC-MS/MS runs in DDA mode were performed on a TripleTOF 5600 mass spectrometer (SCIEX) interfaced with a NanoLC Ultra 2D Plus HPLC system (Eksigent) and a NanoSpray Source. Peptides were directly injected onto a self-packed, 75 µm diameter, 20 cm long fused silica emitter (PicoFrit), self-packed with Magic C18 AQ 3 µm resin (200 Å material, Michrom BioResources), and then separated using a 120 min gradient from 2 – 35% buffer B (buffer A: 0.1% v/v formic acid, 2% v/v acetonitrile; buffer B: 0.1% v/v formic acid, 90% v/v acetonitrile) at a flow rate of 300 nL/min. The data-dependent acquisition parameters were set as follows: MS1 spectra were collected in the range between 360 – 1460 m/z for 500 msec. The 20 most intense precursors with charge state 2 to 5 which exceeded 250 counts per second were selected for fragmentation, and MS2 spectra were collected in the range 50 – 2000 m/z for 150 msec. The precursor ions were dynamically excluded from reselection for 20 sec and the quadrupole resolution was 0.7 Da. Precursors were fragmented with the collision energy equation 0.0625 x m/z - 10.5 with a 15 eV collision energy spread to mimic fragmentation occurring in SWATH-MS mode. The samples Lib1 – Lib 30, as well as the 13 fractions generated by off-gel fractionation were measured in a single MS injection. The samples Lib-31 and Lib-32 were measured in technical duplicates and Lib-33 and Lib-34 in technical triplicates (see Dataset EV2).

### DDA data analysis

All profile-mode WIFF files acquired in DDA mode were centroided and converted to mzXML as described previously (Schubert *et al*, 2015). MS2 spectra were queried against a canonical *E. coli* proteome database from Uniprot (uniprot_ecoli_3AUP000000625 version 2014.10.22, containing 4305 protein sequences) appended with 9 control or antibiotic resistance proteins (Bla - Ampicillin resistance, Cat – chloramphenicol resistance, Neo – kanamycin resistance, GFP – green fluorescent protein, RFP – red fluorescent protein, TetR – repressor of tet promotor, XylR – activator of Pu promotor, BSA – bovine serum albumin, IL13RA1 - interleukin 13 receptor subunit alpha). Further, a decoy proteome database was generated, which contained the reversed peptide sequences for all proteins (in total 8630 protein sequences, including targets and decoys). The spectra were searched using four different search engines in parallel: Comet (Eng *et al*, 2013) (version “2013.02 rev. 2”), Myrimatch (Tabb *et al*, 2007) (version 2.1.138), X!Tandem (Craig & Beavis, 2003) (with k-score plugin (MacLean *et al*, 2006), version 2013.06.15.1) and OMSSA (Geer *et al*, 2004) (version 2.1.9). Carbamidomethyl was set as a fixed modification for cysteines and no further variable modification was specified. Fully tryptic peptides and peptides with up to two missed cleavages were allowed. Precursor mass error was set as 50 ppm and the fragment mass error as 0.05 m/z. The search results were further processed and analyzed through the Trans-Proteomic-Pipeline (Deutsch *et al*, 2010) (TPP version 4.7) using PeptideProphet (Choi & Nesvizhskii, 2008) and the parameters -dDECOY_ -OAPdlIw. The results of the four search engines were combined using iProphet (Shteynberg *et al*, 2011). The combined results were then filtered at a 1% protein FDR using MAYU (Reiter *et al*, 2009), which resulted in an iProphet peptide probability cutoff of 0.983022.

### Spectral library generation

A non-redundant consensus spectral library (Lam *et al*, 2008) was generated from the iProphet results with SpectraST (Lam *et al*, 2007) (TPP version 4.7). During this process, a single consensus spectrum was formed for all peptide precursors that were identified with several spectra. Further, a transformation of retention time information into iRT values (Escher *et al*, 2012) was performed using the linear iRT regression function of SpectraST. The resulting spectral library file (sptxt format) is available at [www.swathatlas.org](http://www.swathatlas.org) ([PASS01421](http://www.peptideatlas.org/PASS/PASS01421)).

### Generation of peptide query parameters for targeted proteomics

To extract peptide query parameters from the spectral library file the python script “spectrast2tsv” was used (<https://pypi.python.org/pypi/msproteomicstools>). This script automatically extracted the six most abundant singly or doubly charged b- and y-ion fragments for each peptide precursor in the range between 350 to 2,000 m/z, excluding the precursor isolation window region. iRT peptides were used to generate normalized retention times for all peptides. The resulting peptide query parameters were outputted as a tsv-file and converted to TraML format (Deutsch *et al*, 2012) using the OpenSWATH tool “ConvertTSVToTraML”. Sequence-shuffled decoys were appended using the OpenSWATH tool “OpenSwathDecoyGenerator”. The resulting *E. coli* spectral library is in various file formats (csv, tsv, xlsx and TraML) freely available and downloadable at [www.swathatlas.org](http://www.swathatlas.org) ([PASS01421](http://www.peptideatlas.org/PASS/PASS01421)).

## Quantitative proteomics using DIA/SWATH

### DIA/SWATH-MS mass spectrometry

Peptide mixtures were measured in SWATH-MS mode on two different SCIEX 5600 TripleTOF mass spectrometers using the same instrument setup and method parameters. Both TripleTOFs were interfaced with an Eksigent NanoLC Ultra 2D Plus HPLC system as described previously (Collins *et al*, 2017; Rosenberger *et al*, 2017b). Peptides were directly injected onto a 20-cm PicoFrit emitter (New Objective, self-packed to 20 cm with Magic C18 AQ 3-μm 200-Å material), and separated using a 60 minutes gradient from 2–35% buffer B (buffer A = 0.1% (v/v) formic acid, 2% (v/v) acetonitrile; buffer B 0.1% (v/v) formic acid, 90% (v/v) acetonitrile) at a flow rate of 300 nL/min. In SWATH-MS mode, the TripleTOF was specifically tuned to optimize the quadrupole settings for the selection of 64 variable wide precursor isolation windows. The applied 64-variable window schema covered the precursor mass range of 400–1,200 m/z. The effective isolation windows were 399.5~408.2, 407.2~415.8, 414.8~422.7, 421.7~429.7, 428.7~437.3, 436.3~444.8, 443.8~451.7, 450.7~458.7, 457.7~466.7, 465.7~473.4, 472.4~478.3, 477.3~485.4, 484.4~491.2, 490.2~497.7, 496.7~504.3, 503.3~511.2, 510.2~518.2, 517.2~525.3, 524.3~533.3, 532.3~540.3, 539.3~546.8, 545.8~554.5, 553.5~561.8, 560.8~568.3, 567.3~575.7, 574.7~582.3, 581.3~588.8, 587.8~595.8, 594.8~601.8, 600.8~608.9, 607.9~616.9, 615.9~624.8, 623.8~632.2, 631.2~640.8, 639.8~647.9, 646.9~654.8, 653.8~661.5, 660.5~670.3, 669.3~678.8, 677.8~687.8, 686.8~696.9, 695.9~706.9, 705.9~715.9, 714.9~726.2, 725.2~737.4, 736.4~746.6, 745.6~757.5, 756.5~767.9, 766.9~779.5, 778.5~792.9, 791.9~807, 806~820, 819~834.2, 833.2~849.4, 848.4~866, 865~884.4, 883.4~899.9, 898.9~919, 918~942.1, 941.1~971.6, 970.6~1006, 1005~1053, 1052~1110.6, 1109.6~1200.5 (including 1 m/z window overlapping). SWATH MS2 spectra were collected from 50 to 2,000 m/z. The collision energy (CE) was optimized for each window according to the calculation for a charge 2+ ion centered upon the window with a spread of 15 eV. An accumulation time (dwell time) of 50 ms was used for all fragment-ion scans in high-sensitivity mode. For each SWATH-MS cycle a precursor ion scan in high-resolution mode was acquired for 250 ms (400-1250 m/z), resulting in a cycle time of ~3.45 s. Nominal resolving power for MS1 and SWATH-MS2 scans were 30,000 and 15,000 respectively. Per MS injection 2 μg of protein amount (4 μL injection volume) was loaded onto the HPLC column. While the 30 samples from Dataset EV2 were acquired on one TripleTOF instrument, the 7 calibration samples as well as the limitation series were acquired on the second instrument with the exact same settings. A detailed comparison of samples measured on the two different TripleTOF 5600 instrument setups revealed no systematic biases or batch-effects between the two instruments. For a detailed overview of all samples measured by DIA/SWATH-MS see Datasets EV2 and EV3.

### DIA/SWATH-MS data analysis with OpenSWATH

The generated DIA/SWATH-MS data was analyzed using the software tool OpenSWATH ([www.openswath.org](http://www.openswath.org)). Briefly, the SWATH WIFF files were first converted to profile mzXML using the tool qtofpeakpicker (Rost *et al*, 2014). Our spectral library and derived peptide query parameters were used as input for the targeted data extraction procedure, for which the same parameters were applied as described previously (Collins *et al.*, 2017). The only parameter exceptions were a fragment ion extraction window of 50 ppm and a 600 s retention time extraction window. The tool PyProphet-cli, an extended version of PyProphet (<https://pypi.python.org/pypi/pyprophet>), optimally combined OpenSWATH peptide query scores into a single discriminant score and estimated q-values using a semi-supervised algorithm (Rosenberger *et al*, 2017a; Teleman *et al*, 2015). PyProphet-cli was run using the experiment-wide and global context with a fixed lambda of 0.8, and the results were filtered with a 1% protein- and peptide false discovery rate (Rosenberger *et al.*, 2017a). The tool TRIC was applied to align extracted and scored peak groups across all runs (Rost *et al*, 2016). For the purpose of absolute protein-level quantification we developed a novel algorithm termed xTop (see Appendix Note S2), which was compared to TopPep1, TopPep3 and iBAQ. The resulting quantitative data matrices for the “calibrarion” samples are available in Dataset EV6. The final results of our “versatile” proteomics pipeline, including the calibration with ribosome profiling, are reported in Datasets EV8 and EV9.

## Ribosome profiling methods

### Culture conditions

Seed cultures of strain NCM3722 and NQ1390 were started in LB from single colonies on freshly streaked plates. Pre-cultures in 20 mL (125 mL flasks) of M9+0.5% glucose (+40 μM 3-MBA for NQ1390) were started by diluting the seed LB cultures 10000× (NCM3722) and 300×. Overnight pre-cultures of MG1655 were started directly in MOPS complete (Neidhardt *et al.*, 1974) (Teknova) from a single colony of a freshly streaked plate. Pre-cultures were diluted (prior to saturation for NCM3722 and NQ1390) to starting OD_600_=0.003 (NCM3722), OD_600_=0.015 (NQ1390), and OD_600_=0.0003 (MG1655) in 250 mL of growth medium (M9+0.5% glucose for NCM3722, M9+0.5% glucose +40 μM 3-MBA for NQ1390, MOPS complete for MG1655). 2.8 L flasks were used. All growth was performed at 37°C under rapid shaking (220 rpm).

Steady-state growth rates (𝜆 = log(2)/𝜏, where 𝜏 is the doubling time) in these conditions were: NCM3722: 𝜆=0.88±0.04 h^-1^ , NQ1390: 𝜆=0.34±0.01 h^-1^, MG1655: 𝜆=2.0±0.1 h^-1^.

### Ribosome profiling

Ribosome profiling was performed as described by Li *et al.* (Li *et al.*, 2014) with slight modifications. Briefly, 250 mL of cell culture (OD_600_ = 0.3) was rapidly filtered at 37°C by passing through a nitrocellulose filter with 200 nm pore size (Supor Membrane Disc Filters, Sigma Aldrich). For NQ1390 cells, the harvest was split in 2 rounds of 125 mL due to filter clogging with larger volumes. Following filtration, cell pellets were rapidly collected using a prewarmed metal table crumber, flash frozen in liquid nitrogen, and combined with 650 μL of frozen droplets of lysis buffer (10 mM MgCl_2_, 100 mM NH_4_Cl, 20 mM Tris pH 8.0, 0.1% NP-40, 0.4% Triton X-100, 100 U/mL DNase I (Sigma-Aldrich), 1 mM chloramphenicol). Filtration and harvest were performed at 37°C adjacent to growth shaker using pre-warmed equipment to minimize perturbation to the gene expression program. Cells and lysis buffer were pulverized in 10 mL canisters (10 mL grinding jars, QIAGEN) prechilled in liquid nitrogen using TissueLyser II (QIAGEN) for 5 cycles of 3 min at 15 Hz. Pulverized lysate was thawed on ice and clarified by centrifugation at 20,000 rcf for 10 min at 4°C. 5 mM CaCl_2_ was added to 0.1 mg of RNA from the clarified lysate containing, which was then digested with 1125 U of micrococcal nuclease (Roche) at 25°C for 1 hr. The reaction was quenched by adding EGTA to 6 mM and moved on ice. The monosome fraction following nuclease digestion was collected using sucrose gradient and the RNA extracted by hot-phenol extraction. In order to multiplex cDNA libraries preparations for other ribosome profiling experiments done in species *B. subtilis*, RNA protected fragments from *E. coli* and *B. subtilis* species were pooled at a 1:1 ratio. This procedure is justified as downstream demultiplexing by alignment of the sequenced reads to the respective genomes is unambiguous, as thoroughly assessed by prior *in silico* mixing of raw data from both species and processing through our synthesis rate quantification pipeline.

Ribosome-protected mRNA fragments were isolated by size excision on a denaturing polyacrylamide gel (15%, TBE-Urea, Thermo Fisher Scientific). Fragments with size ranging from 15 to 45 nucleotides were excised from the gel. The 3’ end of footprints was dephosphorylated using 20 units of T4 polynucleotide kinase (New England Biolabs) at 37°C for one hour. Three picomoles of footprints were ligated to 100 pmole of 5’ adenylated and 3’ end blocked DNA oligo (linker1: 5’App/CTGTAGGCACCATCAAT/3ddC) using truncated T4 RNA ligase 2 K277Q at 37°C for 2.5 hr (25% PEG 8000). The ligated product was purified by size excision on a 10% TBE-Urea polyacrylamide gel (Thermo Fisher Scientific). cDNA was generated by reverse transcription using Superscript III (Thermo Fisher Scientific) at 50°C for 45 min with primer ocj485 (/5Phos/AGATCGGAAGAGCGTCGTGTAGGGAAAGAGTGT/iSp18/CAAGCAGAAGACGGCATACGAGATATTGATGGTGCCTACAG), and isolated by size excision on a 10% TBE-Urea polyacrylamide gel (Thermo Fisher Scientific). Single-stranded cDNA was circularized using 100 U of CircLigase (Epicenter) at 60°C for 2 hr (additional 100 U added after the first hour). Ribosomal RNA fragments were removed using biotin-linked DNA oligos:

/5Biosg/TCATCTCCGGGGGTAGAGCACTGTTTCG
/5Biosg/GGCTAAACCATGCACCGAAGCTGCGGCAG
/5Biosg/AAGGCTGAGGCGTGATGACGAGGCACT
/5Biosg/CGGTGCTGAAGCAACAAATGCCCTGCTT

and MyOne Streptavidin C1 Dynabeads (Thermo Fisher Scientific). After being purified using isopropanol precipitation, the remaining cDNA was amplified using Phusion DNA polymerase (New England Biolabs) with o231 primer (5’CAAGCAGAAGACGGCATACGA) and indexing primers (5’AATGATACGGCGACCACCGAGATCTACACGATCGGAAGAGCACACGTCTGAACTCCAGTCACNNNNNNACACTCTTTCCCTACAC). After 10-15 rounds of PCR amplification, the product was selected by size excision on a 8% TB polyacrylamide gel (Thermo Fisher Scientific).

Sequencing for the ribosome profiling experiment was performed on an Illumina NextSeq500. 3’ linker sequences were stripped. Bowtie v. 1.0.1 (options -v 1 -m 2 -k 2) (Langmead *et al*, 2009) was used for sequence alignment to CP011495.1 (chromosome) and CP011496.1 (F plasmid) for strains NCM3722 and NQ1390, and to NC_000913.2 for strain MG1655. To deal with non-template addition during reverse transcription, reads with a mismatch at their 5’ end had their 5’ end reassigned to the immediate next downstream position. The footprint reads with size between 15 to 42 nucleotides in length were mapped to the genome using the center-weighted approach.

Reads mapped to coding regions:

MG1655 MOPS complete: 4.0 M
NCM3722, M9 + 0.5% glucose: 7.0 M
NQ1390, M9 + 0.5% glucose + 40 μM 3-MBA: 20.5 M

Number of genes with more than 100 reads mapped to coding sequence:

MG1655 MOPS complete: 1501
NCM3722, M9 + 0.5% glucose: 2216
NQ1390, M9 + 0.5% glucose + 40 μM 3-MBA: 3082

### Synthesis rate calculation from ribosome profiling data

Conversion from ribosome footprint reads to protein synthesis rates was performed as described by Li et al. (Li *et al.*, 2014), with slight modifications. Briefly, the absolute protein synthesis rate is proportional to the mean ribosome footprint density across a gene as determined from ribosome profiling. The mean ribosome footprint read density across a gene was calculated by excluding the first and last five codons (to avoid biases from increased ribosome footprint densities arising from initiation and termination). Two corrections were applied.

First, to correct for ribosome pausing at Shine-Dalgarno like sequences (Li *et al*, 2012), we followed the same approach as in Li *et al.* (Li *et al.*, 2014). Specifically, the average ribosome occupancy downstream of each hexanucleotide sequence was determined. A line was fit through these ribosome occupancies versus the affinity of the respective hexanucleotide sequence for the anti-Shine-Dalgarno sequence. This line was then used to adjust the ribosome occupancy at each position in each gene: at each position, the measured occupancy is divided by the expected pause duration (in relative units) based on the strongest hexanucleotide sequence at the 6-11 bases upstream. The adjusted ribosome occupancy is no longer correlated with the anti-Shine-Dalgarno affinity.

Second, the mild 5’ ramp in the ribosome footprint density was corrected for as follows (Li *et al.*, 2012): the ribosome occupancy profiles for genes longer than 175 nt with density above 1 read/nt were smoothed by a traveling average (window size 100 nt) and normalized to the average of the first 100 nt (after the first excluded 5 codons at the 5’ end, see above). For the NQ1390 sample, a short depletion region in footprints of 65 nt was observed, possibly due to the slightly slower filtration step. To avoid biases from this depletion region, the first 25 codons of each gene were excluded for the analysis of the NQ1390 data. The median of these normalized profiles at each position (forming a meta-gene profile) was used to fit an exponential decaying function of the form f(*x*) = A + (1-A)e^-^*^x^*^/D^, where *x* is the position from start of the profile (i.e., distance in the gene minus the excluded length at the 5’ end of the gene), D is the decay length and A is the offset level. The fit parameters obtained for the various samples are listed below. The decaying function f(*x*) was then used to correct for the increased ribosome density at the 5’ end of coding sequences. Specifically, the ribosome occupancy at position x along a gene of total length L (excluding regions not considered at the gene’s 5’ and 3’ ends of genes in our analysis) was weighted by a factor f(L)/f(*x*).

The corrected ribosome occupancy was used to compute the mean density, which was taken as directly proportional to the protein synthesis rate. Overall, the above corrections (combined from ramp and Shine-Dalgarno like sequences) were small.

Ramp parameters:

MG1655, MOPS complete: A= 0.75±0.01, D=690±60 nt
NCM3722, M9 + 0.5% glucose: A=0.3±0.4, D=4500±3000 nt (very weak ramp leads to loosely constrained parameters)
NQ1390, M9 + 0.5% glucose + 40 μM 3-MBA: A=0.53±0.01, D=720±20 nt
Error bars are 95% confidence intervals on the fit.

5^th^ and 95^th^ percentile of fold-change of read density pre vs. post-correction:

MG1655 MOPS complete: 0.86 to 1.18
NCM3722, M9 + 0.5% glucose: 0.89 to 1.12
NQ1390, M9 + 0.5% glucose + 40 μM 3-MBA: 0.91 to 1.18

### Sequencing of flhDC promoter region

The flhDC promoter regions from strains BW25113 (wild-type strain with no mutations in the flhDC regulatory region), NCM3722, EQ59 and Nissle1917 were amplified by PCR using forward primer TTCCTTATTCTGTGAACTTCAGGTGAC and reverse primer GCTAACGTTGTCGC
CATTTCTTC. After gel purifications, the PCR products were subject to sequencing analyses by the same primers. Pairwise sequence alignments were performed to determine if there are any mutations between BW25113 and each of other strains. EQ59 has the wild-type flhDC promoter region, with 100% identity in the 725nt region probed. Nissle1917 has a number of mismatches (94.5% identity) in the same region. Instead, the promoter region of NCM3722 could not be amplified using the same primers due to the presence of the large insertion element (Lyons *et al*, 2011b).

# Note S1: Absolute protein mass fractions and concentrations

This note discusses the absolute quantification of protein abundances in terms of mass fractions and number fractions, the possibility of estimating those from ribosome profiling data, and the quantification of protein concentrations.

Absolute quantification can refer to two different concepts, which is best keeping distinct. In the first case, it refers to the ability of comparing the intensities of *different* proteins in the *same* sample, as opposed to relative quantification, i.e. the comparison of the intensities of the *same* protein across *different* samples. The second meaning of absolute quantification is the computation of cellular protein concentrations, expressed often in units of protein per cell or per cytoplasmic volume. However, this requires additional information, such as the average cellular volume or the total abundance of proteins per cell, which might potentially change from sample to sample, especially if the samples originate form cell cultures characterized by different physiological states. In this work, “absolute protein quantification” refers to the first of the two meanings, while we use “quantification of protein concentrations” for the second one.

### Absolute protein quantification and protein mass fractions

The mass fractions $\phi_{k}$, which we use extensively in this work, provide a measure of absolute protein abundance. They are defined as:

|  | $\phi_{k}=\frac{mass of k^{\mathrm{th}} protein in the sample}{total protein mass in the sample} .$ | (N1.1) |
| --- | --- | --- |

The definition ensures that the sum over all mass fractions yields 1, $\sum_{k} \phi_{k}=1$. A similar absolute quantity is provided by the protein abundance per total protein mass (in units such as mol/g), obtained by dividing the protein mass fraction by the molecular weight $\mu_{k}$ of the corresponding protein:

|  | $\frac{\phi_{k}}{\mu_{k}}=\frac{amount of k^{\mathrm{th}} protein in the sample}{total protein mass in the sample} .$ | (N1.2) |
| --- | --- | --- |

When the protein intensities allow to compare reliably the concentrations of different proteins in the same sample, then each of these two quantities can be computed from the knowledge of protein intensities and the specific weight of each protein. Both xTop, TopPep1/3 and iBAQ intensities (which we indicate here as $I_{k}$ for the *k*^th^ protein) are defined so as to best reflect protein copy numbers or concentrations, but they are not normalized to an absolute scale. This means that, while the ratio ${I_{j}}/{I_{k}}$ gives (in principle) the ratio of the concentrations or copy numbers of proteins *j* and *k* in the same sample, the values of $I_{j}$ and $I_{k}$ do not have any particular meaning *per se*. However, the ratio of two mass fractions ${\phi_{j}}/{\phi_{k}}$ can be computed as:

|  | $\frac{\phi_{j}}{\phi_{k}}=\frac{\mu_{j}I_{j}}{\mu_{k}I_{k}}$ | (N1.3) |
| --- | --- | --- |

By summing over all proteins $j$ and taking the reciprocal of both sides, one obtains an expression for the absolute mass fraction in terms of the protein intensities:

| (protein mass fractions) | $\phi_{k}=\frac{\mu_{k}I_{k}}{\sum_{j} \mu_{j}I_{j}}$ | (N1.4) |
| --- | --- | --- |

The denominator $\sum_{j} \mu_{j}I_{j}$ enforces the normalization $\sum_{k} \phi_{k}=1$. Of course, this expression relies on the assumption that the ratio of protein intensities corresponds to the ratio of the corresponding concentrations, underpinning Eq. (N2.3); proteins that are detected less (or more) efficiently than the average will suffer an underestimation (overestimation) for their absolute mass fraction. For a given protein, if the detection efficiency varies across different samples compared to the average detection efficiency, both the relative ($\phi_{k}$ evaluated across different samples) and the absolute ($\phi_{k}$ vs $\phi_{j}$ in the same sample) protein quantification will be affected. If instead the detection efficiency is a fixed multiple $c$ of the average detection efficiency across different samples, then only the absolute quantification is affected, with $\phi_{k}/\phi_{j}$ being overestimated by the same factor $c$. For these reasons, in this work we rely on xTop protein intensities, which provide consistent relative quantification, which are calibrated by ribosome profiling to compensate for different protein detection efficiencies, as discussed in the following section.

As a side note, we observe that the most direct way to normalize the protein intensities is by dividing them by their sum. Doing so allows to define protein number fractions $\psi_{k}$, representing the amount (copy number) of the $k^{th}$ protein divided by the total amount (copy number) of proteins in the sample:

| (protein number fractions) | $\psi_{k}=\frac{I_{k}}{\sum_{j} I_{j}}$ | (N1.5) |
| --- | --- | --- |

From the definition, it is clear that $\psi_{k}/\psi_{j}$ equals the ratio of the concentrations or copy numbers of the corresponding proteins. This can also be considered an absolute quantity, except that the total number of proteins in a sample cannot be easily quantified experimentally, complicating comparisons across different samples. Nevertheless, number and mass fractions are completely equivalent, since it is possible to convert ones into the others using the protein molecular weights:

|  | $\psi_{k}=\frac{\phi_{k}/\mu_{k}}{\sum_{j} \phi_{j}/\mu_{j}} , \phi_{k}=\frac{{\mu_{k}\psi}_{k}}{\sum_{j} \mu_{j}\psi_{j}}$ | (N1.6) |
| --- | --- | --- |

Here, $\sum_{k} \mu_{k}\psi_{k}$ represents the average molecular weight of a protein in the sample. Eq. (N1.6) shows that if the molecular mass of protein $k$is equal to the average molecular weight, $\mu_{k}=\sum_{j} \mu_{j}\psi_{j}$, then the corresponding number and mass fractions match, $\phi_{k}=\psi_{k}$. However, the two can be very different for very large or, in particular, very small proteins.

## Ribosome profiling and absolute protein quantification

As explained in detail in the extended experimental methods, ribosome profiling allows to measure the mean density of ribosomes on the mRNA, which is taken to be proportional to the protein synthesis rates, allowing for cross-gene comparisons in the same sample, i.e. they are an “absolute” measure of protein synthesis rate. The proportionality of ribosome density and synthesis rates depends on a few assumptions, most importantly (1) that most elongating proteins ribosomes successfully terminate translation, and (2) that the ribosome elongation speed is similar across different mRNA species. These conditions are met in exponentially growing *E. coli* cells, which are the subject of our study.

Furthermore, in exponential (balanced) growth, protein concentrations $[P_{k}]$ do not vary over time, implying that protein synthesis fluxes $J_{P,k}$ has to match the sum of three components: the protein dilution flux, $\lambda[P_{k}]$ where $\lambda$ represent the cellular growth rate; the protein degradation rate usually taken to satisfy first-order kinetics, $\delta_{k}[P_{k}]$; and the flux $J_{ex,k}$ describing the excretion of protein in the extracellular environment:

|  | $J_{P,k}=\left( \lambda+\delta_{k} \right)\left[ P_{k} \right]+J_{\mathrm{ex},k}$ | (N1.7) |
| --- | --- | --- |

For exponentially growing *E. coli*, studies have shown that protein degradation is limited to a few specific cases, including the stress sigma factor RpoS, and have found no significant contribution of protein degradation to the total protein synthesis flux (Koch & Levy, 1955). Similarly, protein excretion is limited for few specific systems, such as the flagellar anti-sigma factor FlgM. So, for most proteins, the protein degradation rate is much smaller than the growth rate, $\delta_{k}\ll\lambda$, and the excretion rate is negligibile compared to protein synthesis, $J_{\mathrm{ex},k}\ll J_{P,k}$, leading to :

|  | $J_{P,k}=\lambda\left[ P_{k} \right]$ | (N1.8) |
| --- | --- | --- |

So, for the vast majority of proteins, the protein synthesis flux is directly proportional to the protein concentration via the same proportionality coefficient (the growth rate). Since the protein synthesis rates from ribosome sequencing, $R_{k}$, are proportional to $J_{P,k}$, they are proportional to the protein concentration $[P_{k}]$, too, and they can be treated in the same way as the protein intensities $I_{k}$. In particular, it is possible to compute the ribosome-profiling-based estimate $\rho_{k}$ of protein mass fractions as:

|  | $\rho_{k}=\frac{\mu_{k}R_{k}}{\sum_{j} {\mu_{j}R}_{j}}$ | (N1.9) |
| --- | --- | --- |

An advantage of ribosome profiling compared to proteomics is that ribosome profiling rates are based on RNA sequencing, which is less susceptible to sequence-dependent biases compared to the vast variability of peptide precursors detection efficiencies. To test the accuracy of ribosome sequencing Li et al. (Li *et al.*, 2014) compared the synthesis rates computed using the reads mapping to the first half of the genes to those computed mapping to the second half, finding that most synthesis rates coincide within a 30% margin. Furthermore, synthesis rates of selected proteins known to be associated in complexes match well with the expected stoichiometry. On the other hand, the vast variability (orders of magnitude) of peptide precursor intensities due to varying detection efficiencies does not allow to easily obtain an accurate protein intensity.

In summary, ribosome profiling can provide accurate and precise estimates of absolute protein abundances as long as the bulk of the proteome is stable (not degraded) and is not transported in the extracellular media, conditions that are satisfied by exponentially growing *E. coli* cells. This comes with the caveat that it will overestimate the protein mass fractions of proteins whose degradation rate is comparable or faster than the growth rate ($\delta_{k}\gtrsim\lambda$) or are actively transported outside the cell.

## Quantification of protein concentrations

While the absolute quantification in terms of protein mass fractions (or protein copy abundance per total protein mass) is extremely valuable, actual cellular concentrations can be necessary for molecular biology and biochemistry point of view. However, the evaluation of protein concentrations, usually in “per cell” or “per volume” units, requires the knowledge of the conversion factor between total protein mass and total cell number or total cellular volume. If such conversion factor depends on the growth condition of the cells, it can crucially influence relative (cross-sample) comparisons of protein concentrations.

For *E. coli* cells, the average cell volume changes dramatically with growth conditions, varying more than fourfold between slow and fast growth (see e.g. (Basan *et al*, 2015b)). On the other hand, protein concentrations (protein mass or abundance per cellular volume) can be estimated directly from the protein mass fractions. In fact, total protein mass is found to be approximately proportional to the optical density of the growth culture at 600 nm ($\mathrm{OD}_{600}$). Since the density of the cell (dry mass per cellular volume) is found to be roughly constant across different growth conditions, protein concentrations can be estimated from optical density measurements, if the mass fraction of the same protein over the total protein mass is known. In formulas, the abundance of the *k*-th in units of mass per volume is given by the following expression:

|  | $\rho_{k}=\phi_{k}\cdot\rho_{TP} , \rho_{TP}=13.5\cdot{10}^{-8} \mu g/\mu m^{3} .$ | (N1.10) |
| --- | --- | --- |

The total protein density $\rho_{TP}$ is computed from the observed cellular volumes and protein masses across several growth conditions (as summarized in Mori *et al.* (Mori *et al*, 2017), Figure S1, based on data from Basan et al. (Basan *et al.*, 2015b)). From here, the concentration of the protein can be obtained by dividing $\rho_{i}$ by the protein molecular weight; if the cellular volume is known for the condition of interest, one can then obtain the protein copy number per cell. Alternatively, it is possible to compute the total protein concentration $[P]$ as the ratio between the total protein mass $\rho_{TP}$ and average protein mass $\left\langle\mu\right\rangle=\sum_{k} \psi_{k}\mu_{k}$. In our data, we observe that $\left\langle\mu\right\rangle$ is roughly constant, $\sim4.4\cdot{10}^{-14} \mu g$, leading to a constant total concentration of about $3\cdot{10}^{6}$ proteins per $\mu m^{3}$. Hence, concentrations of individual proteins can be expressed as:

|  | $\left[ P_{k} \right]=\psi_{k}\cdot\left[ P \right] , \left[ P \right]=3\cdot{10}^{6}/\mu m^{3} .$ | (N1.11) |
| --- | --- | --- |

As a concrete example, consider an average-sized protein with $l_{k}=240$ residues and an absolute abundance close at our detection limit $\phi_{k}={10}^{-5}$ (Appendix Figure S4A). The mass of this protein is close to the average protein mass, $\mu_{k}=\langle\mu\rangle$, and hence number and mass protein fractions coincide, $\psi=\phi$. For a fraction $\psi={10}^{-5}$, Eq. (N1.11) yields a protein concentration $[P_{k}]= 30/\mu m^{3}$. For cells growing in glucose minimal media, in which the average cellular volume is about $2 \mu m^{3}$ (Basan *et al.*, 2015b), this corresponds to about 60 proteins per cell. At faster growth, since cellular volumes are about 2-fold larger, this value increases above 120 copies/cell, while it reduces to about 15/cell at slow, carbon-limited growth. The concentrations of proteins with different mass fractions $\phi_{k}$ and protein mass $\mu_{k}$ are easily computed from the same expression: at fixed protein mass fraction, proteins that are twice (half) as large as the average protein will have half (twice) their concentrations.

# Note S2: xTop protein intensities

The main motivation for the introduction of the xTop protein intensities is shown in Figure N2.1 (top row), where we study the peptide precursor intensities for three exemplary proteins (Sbp, YgeR and FadH) in *E. coli* cells subject to different degrees of carbon-limitation via titration of the glucose transporter PtsG (15 distinct samples). Cultures in which the transporter is induced less are characterized by slower growth rates, which represent a convenient independent variable against which to plot peptide and protein intensities. We made the following observations:

- The peptide precursor intensities have a smooth dependence on the growth rate. This is expected since the data at similar growth rates correspond to similar degree of carbon starvation.
- In these examples, not all the peptides are detected across all conditions. In the case of Sbp, many peptides are not detected at slow growth, while for FadH peptides are mostly missing at fast growth.
- The most abundant peptides are not necessarily the ones detected most consistently. This is clearly seen in the case of YgeR, where the most consistently detected peptide precursor has the lowest intensity among the three peptide precursors highlighted in colored circles.
- Given the heterogeneity in peptide detection across samples, the total peptide intensity $I_{ps}^{\mathrm{all}}=\sum_{p} I_{ps}$ appears to either have inflate the fold-change compared to that of the peptide precursors (for Sbp and FadH), or behave erratically when the peptide precursors yielding the largest intensities are only detected in a few samples (as for YgeR).

These observations prompted us to define a novel protein quantification method, termed *xTop*, which is designed to be minimally susceptible to absent or noisy peptide precursor intensities. The aim of this Note is to detail the logic and the concrete implementation of method. We show in Figure N2.1, bottom row, *xTop* protein intensities (red circles) for the three cases discussed above. The xTop intensities smoothly vary with the growth rates, correlating with the intensities of individual peptide precursors, and are minimally impacted by missing peptides intensities. For comparison, we show iBAQ protein intensities, which are proportional to $I_{ps}^{\mathrm{all}}$ and are hence strongly impacted by missing or noisy peptides.


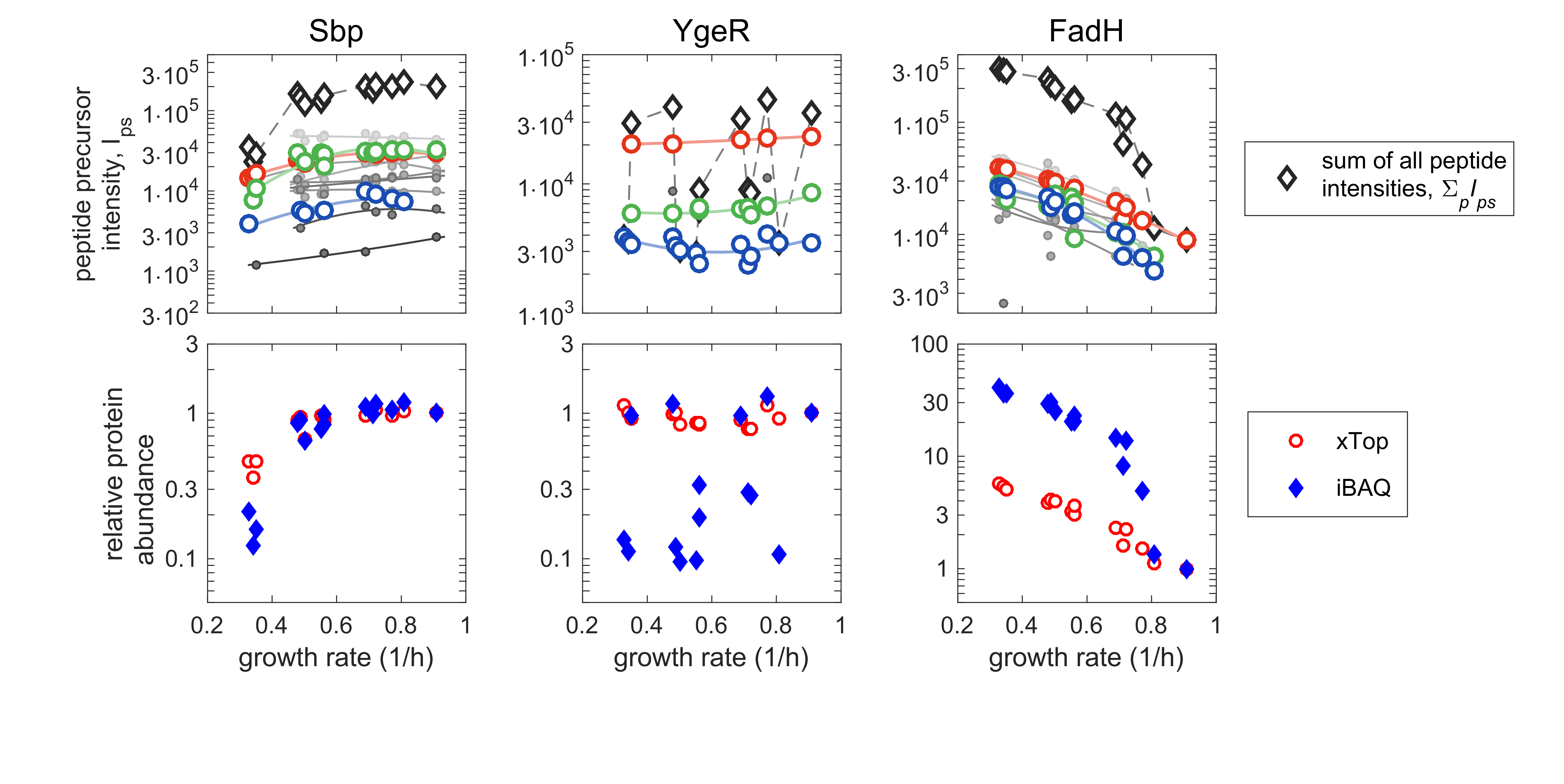


Figure N2.1. Top row: peptide precursor intensities for three representative proteins (left to right: Sbp, YgeR, FadH) in carbon-limited conditions (C-limitation) as a function of the growth rate. The intensities are shown as grey circles, with smooth eye guides of the same color; peptide precursors with good coverage across all samples are highlighted using red, green or blue colors. The black open diamonds indicate the sum of all peptide intensities, $I_{s}^{\mathrm{all}}=\sum_{p} I_{ps}$. Bottom row: xTop protein intensity (red circles) and iBAQ protein intensities (blue diamonds), normalized to the maximum growth condition. iBAQ protein intensities are proportional to the sum of all peptide precursor intensities from the top row. Instead, xTop mirrors more closely the growth dependence of individual peptides (colored circles in the top row).

## Definition of the xTop protein intensities

The first step to compute the xTop intensities is to rank the peptide precursors by total intensity, as described in the Methods. Then, we organized the peptide intensities in a matrix $I_{ps}$ whose components are the intensity of the peptide precursor $p=1,\ldots,N_{P}$ in sample $s=1,\ldots,N_{S}$. We assume that the peptides have been sorted in decreasing order, so that the TopPepN protein intensity is given by $I_{s}^{\mathrm{TopN}}\equiv\sum_{p=1}^{N} I_{ps}$; in particular, TopPep1 protein intensity is simply given by $I_{1s}$.

The xTop estimator $I_{s}^{\mathrm{xTop}}$ is a refinement of $I_{1s}$, obtained by modelling the intensity of each peptide as the product of two factors: the sample-dependent intensity $I_{s}^{\mathrm{xTop}}$, and the peptide-dependent detection efficiency $\varepsilon_{p}$ (see also Fig. N2.2 below):

|  | $I_{ps}\approx I_{s}^{\mathrm{xTop}}\times\varepsilon_{p} .$ | (N2.1) |
| --- | --- | --- |

For the top peptide precursor we take the efficiency to be one, $\varepsilon_{1}=1$, so that $I_{1s}\approx I_{s}^{\mathrm{xTop}}$; instead, the detection efficiencies of the other peptide precursors will be less than one and are determined from the data. While $I_{s}^{\mathrm{xTop}}$ matches on average the intensity of the top peptide, its value is also affected by the intensities of all other peptides, and in particular by their variation, for each peptide, across the different samples. To combine the different intensities we also take into account the fact that different peptides precursors not only have different average intensities, but they are also more or less consistent, i.e. $I_{ps}$ will fluctuate around its “true” value (which we estimate as $I_{s}^{\mathrm{xTop}}\times\varepsilon_{p}$) with different amplitudes for different peptides *p*; the scatter amplitudes are also to be determined from the dataset. This allows us to combine the signals from the different peptides into the xTop estimator weighing more the most consistent peptides (with smaller fluctuations).

After the xTop protein intensities and the detection efficiencies have been calculated – as we describe in the next section – it is possible to further “adjust” the absolute scale of the protein intensities with a protein-dependent scale factor, thus affecting the absolute quantification of the proteome (but not the relative one). As explained above, $I_{s}^{\mathrm{xTop}}$ is a more accurate version of the TopPep1 protein intensity, $I_{s}^{TopPep1}=I_{1s}$. The equivalent of the TopPep3 protein intensity ($I_{s}^{TopPep3}=I_{1s}+I_{2s}+I_{3s}$) is obtained by multiplying the xTop intensities by a factor $\sum_{p=1}^{3} \varepsilon_{p}=(1+\varepsilon_{2}+\varepsilon_{3})$ which accounts for the abundances of the Top 3 peptide precursors. iBAQ-like protein intensities can be computed in a similar way, by multiplying $I_{s}^{\mathrm{xTop}}$ by the sum of all peptide efficiencies, $\sum_{p=1}^{P} \varepsilon_{p}$, and dividing by the number of fully tryptic peptides expected for the protein of interest. Another possibility is to rescale the protein intensities with a factor dependent on the protein size: as we show in Appendix Note S3, a scaling factor $\left( number of residues \right)^{0.6}$ can significantly improve the agreement between xTop/TopPep1/TopPep3 protein intensities and ribosome profiling data. Given the vast range of possibilities, we left the detailed study of different possible scaling factors for future investigations, while instead making use of the ribosome profiling synthesis rates to improve the absolute quantification of xTop.

## Maximum a posteriori (MAP) estimators of xTop intensities

In order to compute the xTop intensity $I_{s}^{\mathrm{xTop}}$ and the peptide detection efficiencies $\varepsilon_{p}$ appearing in Eq. (N2.1) from the empirical peptide precursor intensities $I_{ps}$, we used a common statistical technique named Maximum *a Posteriori* probability (MAP) estimation, rooted in the Bayesian framework.

Following Bayes theorem, a “posterior” probability distribution for the model parameters $\vec{\theta}$ as a function of the experimental data $\vec{y}$, $p_{\mathrm{post}}\left( \vec{\theta} | \vec{y} \right)$, is computed as the product of two terms: the “likelihood” $\mathcal{L}\left( \vec{y} | \vec{\theta} \right)$, which describes the statistical model used to analyze the data, and the “prior” $p_{\mathrm{prior}}(\theta)$, encapsulating additional information on the model parameters. The MAP estimators are the parameters $\vec{\theta}_{\mathrm{MAP}}$ that maximize the posterior probability distribution $p_{\mathrm{post}}\left( \vec{\theta} | \vec{y} \right)$, and are thus the most likely parameters given the experimental data and the statistical model employed.


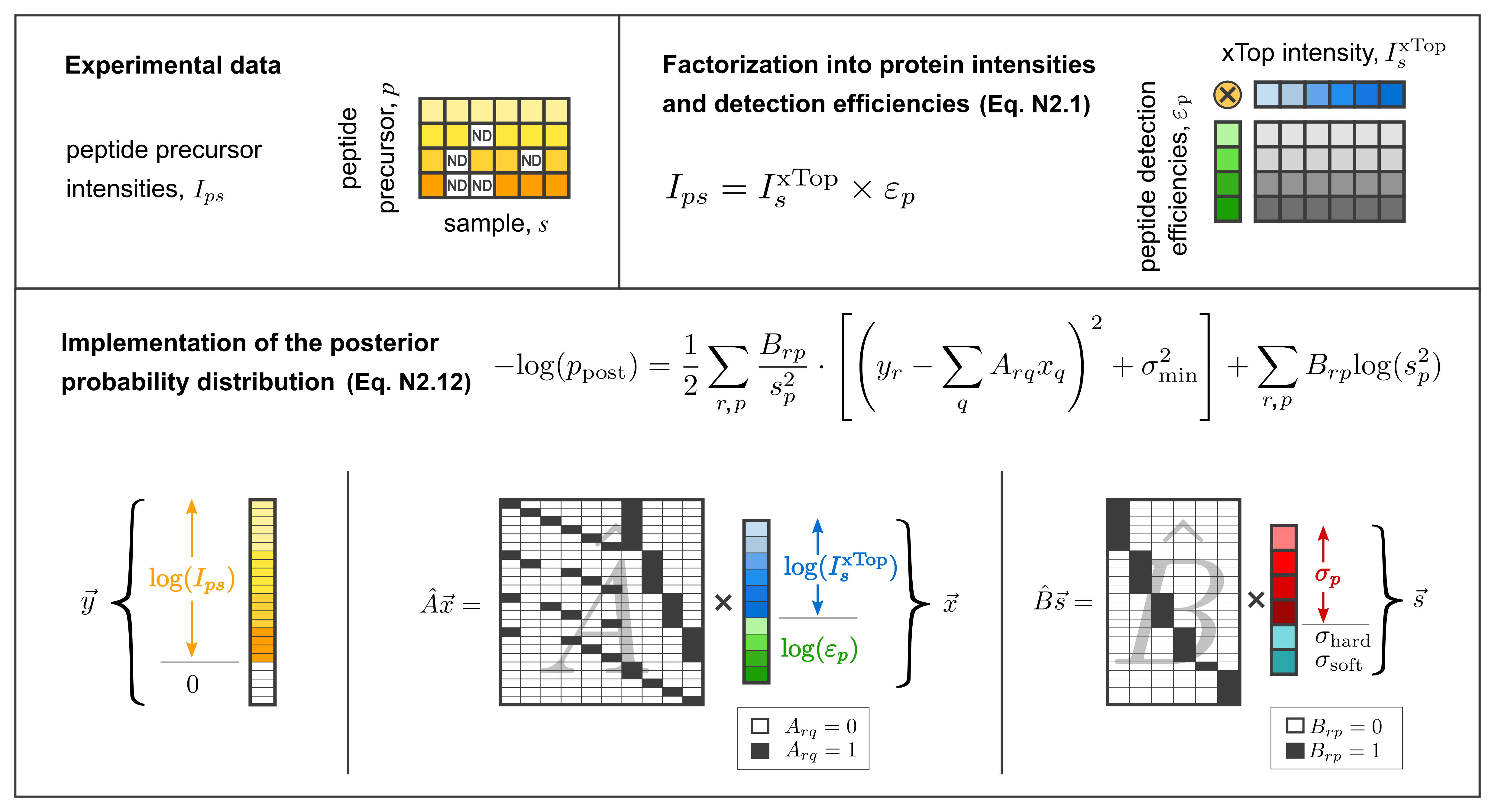


**Figure N2.2. Definition of the xTop protein intensities and implementation of the MAP estimators.** For each protein in the dataset at hand, the peptide precursor intensities (yellow) are arranged in a matrix $I_{ps}$ (top-left panel; ND, not detected). In the xTop approach (top-right panel), this intensity matrix is modeled (top-right panel) as the product of a sample-dependent xTop protein intensity $I_{s}^{\mathrm{xTop}}$ (blue) and a peptide-dependent detection efficiency coefficient $\varepsilon_{p}$ (green). In the bottom panel we describe the transformations introduced in the text of this Note to write down in a compact and algorithmically efficient way the posterior probability distribution, including the vector $\vec{y}$ which includes the log-transformed experimental intensities; the vector $\vec{x}$ including the log-transformed xTop intensities and detection efficiencies; the vector  $\vec{s}$ including the scatter associated to each peptide precursor.

In our case, Eq. (N2.1) describes the deterministic part of the model included in the likelihood function. We take the intensities of each peptide to be log-normally distributed. This assumption is both convenient from the analytical point of view and supported by the small and scatter of the log-transformed peptide intensities across replicates seen in Appendix Figure S1. We further assume that the scatter $\sigma_{p}$ for the log-transformed intensity of peptide $p$ is sample-independent. Therefore, the statistical model can be written as:

|  | $\log I_{ps}=\log I_{s}^{\mathrm{xTop}}+\log\varepsilon_{p}+\sigma_{p}\cdot\mathrm{Gauss}\left( 0,1 \right) ,$ | (N2.2) |
| --- | --- | --- |

where $\mathrm{Gauss}(0,1)$ is a Gaussian random variable with zero mean and unit variance. With this choice, the likelihood $\mathcal{L}$ of the intensity $I_{ps}$ takes the following form:

|  | $\mathcal{L}\left( I_{ps} \vert I_{s}^{\mathrm{xTop}},\varepsilon_{p},\sigma_{p}^{2} \right)=\frac{1}{\sqrt{2\pi\sigma_{p}^{2}}}\exp\left( -\frac{1}{2}\frac{\left( \log I_{ps}-\log I_{s}^{\mathrm{xTop}}-\log\varepsilon_{p} \right)^{2}}{\sigma_{p}^{2}} \right) .$ | (N2.3) |
| --- | --- | --- |

The posterior probability is then given as:

|  | $p_{\mathrm{post}}\left( I^{\mathrm{xTop}},\varepsilon,\sigma^{2}\vert I \right)=\prod_{ps} \mathcal{L}\left( I_{ps} \vert I_{s}^{\mathrm{xTop}},\varepsilon_{p},\sigma_{p}^{2} \right)\times p_{\mathrm{prior}}(I_{s}^{\mathrm{xTop}},\varepsilon_{p},\sigma_{p}^{2}) .$ | (N2.4) |
| --- | --- | --- |

The MAP estimators are then obtained by substituting the experimental values $I_{ps}$ into Eq. (N2.4) and maximizing $p_{\mathrm{post}}$ as a function of $I_{s}^{\mathrm{xTop}}$, $\varepsilon_{p}$ and $\sigma_{p}^{2}$. We take the prior term $p_{\mathrm{prior}}$ to be the product of three terms. The first term is needed to make sure that the efficiency of the top peptide precursor is set to 1:

|  | $p_{\mathrm{prior}}^{(1)}=\exp\left( \frac{1}{2}\frac{\left( \log\varepsilon_{1} \right)^{2}}{\sigma_{hard}^{2}} \right) ,$ | (N2.5) |
| --- | --- | --- |

where $\sigma_{\mathrm{hard}}^{2}$ is a small parameter (we found that ${10}^{-4}$ or less works well for our purposes) in order to force $\log\varepsilon_{1}$ to zero, and hence $\varepsilon_{1}$ to unity. A second term is needed in the (exceedingly rare) case in which some peptides are only detected in samples in which no other peptides (including the Top1 peptide) are detected. In this case it is not possible to set their detection efficiency relative to the Top1 peptide, so we decided to set it to 1 with the following term:

|  | $p_{\mathrm{prior}}^{(2)}=\prod_{p} \exp\left( \frac{1}{2}\frac{\left( \log\varepsilon_{p} \right)^{2}}{\sigma_{\mathrm{soft}}^{2}} \right) .$ | (N2.6) |
| --- | --- | --- |

Setting $\sigma_{\mathrm{soft}}^{2}$ to a value much larger than the actual spread of peptide precursors (e.g. ${10}^{2}$ or larger) ensures that the solutions are not altered significantly. This term is only relevant in exceptional situations in which the protein is badly detected across most samples, and its main purpose is to ensure that the optimization of the posterior probability converges to a finite result even in these ill-behaved cases.

These first two prior terms are only introduced for technical convenience. For instance, the first term can be avoided by setting $\varepsilon_{1}=1$ from the start, instead of treating it as a variable. In both cases the MAP estimators do not depend on the precise values of $\sigma_{\mathrm{hard}}$ and $\sigma_{\mathrm{soft}}$. However, as explained in detail below, it is also necessary to introduce a minimum value $\sigma_{\min}$ of the scatter of each peptide in order to make the MAP estimators well-defined. This is done by employing a third prior term:

|  | $p_{\mathrm{prior}}^{(3)}=\prod_{p} \exp\left( +\frac{n_{p}}{2}\frac{\sigma_{\min}^{2}}{\sigma_{p}^{2}} \right) ,$ | (N2.7) |
| --- | --- | --- |

where $n_{p}$ is equal to the number of samples in which the peptide $p$ is detected. Importantly, $\sigma_{\min}^{2}$ should be regarded as a parameter that has to be tuned in order to obtain the best performance with xTop. We will discuss both the rationale behind the specific functional form of $p_{\mathrm{prior}}^{(3)}$ and the effect of varying $\sigma_{\min}^{2}$in the following sections.

## Calculation of MAP estimators

Here below we describe in detail the procedure we followed to generate the xTop protein intensities. This procedure is carried out separately for each protein of interest: xTop intensities for different proteins are independent from each other. We name $P$ the number of peptide precursors associated to the protein of interest, and $S$ the number of samples.

Firstly, we only take into account peptide precursors which are detected in at least two samples, since it is necessary to estimate the scatter. If only one peptide precursor is available, then we set $I_{s}^{\mathrm{xTop}}$ to be equal to the intensity of that peptide precursor.

To compute the MAP estimators, it is necessary to write Eq. (N2.4) in a form that allows it to be maximized algorithmically. As shown in Figure N2.2, we collect all log-transformed intensities in a column vector

|  | $\vec{y}=\left( \log I_{11},\log I_{12},\ldots,\log I_{PS} \right)^{T}$ | (N2.8) |
| --- | --- | --- |

with entries $y_{r}$ labeled by and index $r=1,\ldots,R$, skipping non-detected peptides (hence $R\leq PS)$. Similarly, we gather both the xTop intensities and the efficiencies in a vector

|  | $\vec{x}=\left( \log I_{1}^{\mathrm{xTop}},\log I_{2}^{\mathrm{xTop}},\ldots,\log I_{S}^{\mathrm{xTop}},\log\varepsilon_{1},\ldots,\log\varepsilon_{P} \right)^{T}$ | (N2.9) |
| --- | --- | --- |

with entries $x_{q}$ labeled by the index $q=1,\ldots,Q$. This formulation allows us to rewrite Eq. (N2.1) in the form of a canonical multilinear model:

|  | $y_{r}\approx\sum_{q} A_{rq}x_{q} ,$ | (N2.10) |
| --- | --- | --- |

where the matrix $\hat{A}$ connects the intensities in the vector $\vec{y}$ to the corresponding xTop intensities and peptide efficiencies (see example in Fig. N2.2). Similarly, we need to map the peptide-specific standard deviations $\sigma_{p}$ to each component $y_{r}$ of the vector $\vec{y}$ with a matrix $\hat{B}$ with components $B_{rp}$. Note that this latter matrix satisfies two simple sum rules, $\sum_{p} B_{rp}=1$ and $\sum_{r} B_{rp}=n_{p}$ (the number of samples in which peptide precursor $p$ has been detected).

Finally, in order to account for the prior terms involving the peptide efficiencies, we define a vector

|  | $\vec{s}=\left( \sigma_{1},\sigma_{2},\ldots,\sigma_{P},\sigma_{\mathrm{hard}},\sigma_{\mathrm{soft}} \right)^{T}$ | (N2.11) |
| --- | --- | --- |

which includes both the peptide-specific standard deviations $\sigma_{p}$ and the two parameters $\sigma_{\mathrm{hard}}$ and $\sigma_{\mathrm{soft}}$; the quadratic terms in $p_{\mathrm{prior}}^{(1)}$and $p_{\mathrm{prior}}^{(2)}$ are then included by adding a correspondent number of rows to both $\vec{y}$ (zero entries) and the matrices $A_{rq}$ and $B_{rp}$, as illustrated in Fig. N2.2.

Maximizing the posterior probability distribution is equivalent to minimizing its negative logarithm, $L\equiv-\log\left( p_{\mathrm{post}} \right)$, which has however a simpler mathematical form. We compute the MAP estimators as the set of parameters $\vec{x}$ and  $\vec{\sigma^{2}}$ that minimize $L$. Using all the substitutions described above, Eq. (N2.8-N2.11), we can write:

|  | $L\left( \vec{x},\vec{s^{2}} \right)=\frac{1}{2}\sum_{r,p} B_{rp}\left[ \frac{\left( y_{r}-\sum_{q} A_{rq}x_{q} \right)^{2}}{s_{p}^{2}}+\frac{\sigma_{\min}^{2}}{s_{p}^{2}}+\log s_{p}^{2} \right]$ | (N2.12) |
| --- | --- | --- |

where we used $\log\left( \sum_{r} B_{rp}\sigma_{p}^{2} \right)=\sum_{r} B_{rp}\log\sigma_{p}^{2}$ which is implied by the definition of $\hat{B}$ (more specifically, by the fact that $B_{rp}$ is equal to one only for one index $p$ for every choice of $r)$.

The expression in Eq. (N2.12) is minimized when the partial derivatives of $L$ with respect to either $x_{q}$ or $s_{p}^{2}$ vanish. In the first case, $\partial L/\partial x_{q}=0$ leads to the linear set of constraints for $\vec{x}$ (at fixed  $\vec{s^{2}}$):

|  | $\sum_{q'} \left( \sum_{r,p} A_{qr}^{T}\frac{B_{rp}}{s_{p}^{2}}A_{rq'} \right)x_{q'}=\sum_{r,p} A_{qr}^{T}\frac{B_{rp}}{s_{p}^{2}}y_{r}$ | (N2.13) |
| --- | --- | --- |

Another set of equations is obtained by taking the partial derivative against the (squared) standard deviations for each peptide precursor *p*, i.e. $\partial L/\partial s_{p}^{2}=0$. Since $s_{p}=\sigma_{p}$ for $p\leq P$ (we do not take derivates against $\sigma_{\mathrm{hard}}^{2}$ and $\sigma_{\mathrm{soft}}^{2}$, as they are fixed parameters), we can write:

|  | $\sigma_{p}^{2}=\frac{\sum_{r} B_{rp}\left( y_{r}-A_{rq}x_{q} \right)^{2}}{\sum_{r} B_{rp}}+\sigma_{\min}^{2}$ | (N2.14) |
| --- | --- | --- |

which directly provides the variances associated to each peptide precursor. In the light of Eq. (N2.14), it is now clear that the functional form of $p_{\mathrm{prior}}^{(3)}$, Eq. (N2.7), is precisely the one needed to enforce the minimal value $\sigma_{\min}^{2}$ for all peptide variances. We will discuss in a following section how the xTop intensities depend on the value of $\sigma_{\min}^{2}$, and we will show that the performance of the xTop estimator is consistently good for a wide range of $\sigma_{\min}^{2}$ values across several orders of magnitude.

Equations (N2.13) and (N2.14) are jointly solved by using a commonly used iterative procedure. Starting with a reasonable initial value $\vec{\sigma_{[0]}^{2}}$ for the components of the vector $\vec{\sigma^{2}}$ (e.g. ${10}^{-2}$, between $\sigma_{\mathrm{hard}}^{2}$ and $\sigma_{\mathrm{soft}}^{2}$), we solve the linear system (N2.13) to obtain the initial estimate $\vec{x}_{[1]}$; this value is then inserted in Eq. (N2.14), which is then used to compute $\vec{\sigma_{[1]}^{2}}$. These steps are then iterated, leading to the pairs of estimates $\left( \vec{x}_{\left[ 2 \right]},\vec{\sigma_{\left[ 2 \right]}^{2}} \right)$, $\ldots$ , $\left( \vec{x}_{\left[ k \right]},\vec{\sigma_{\left[ k \right]}^{2}} \right)$. By alternatively setting to zero the two expression Eq. (N2.13)-(N2.14), the sequence $L_{k}\equiv L\left( \vec{x}_{\left[ k \right]},\vec{s_{\left[ k \right]}^{2}} \right)$ is progressively minimized, i.e. $L_{k+1}\leq L_{k}$. It is also easy to see, substituting Eq. (N2.14) into (N2.12), that each term is bounded from below by a finite constant $L_{\min}$, as long as $s_{\min}^{2}$ is strictly positive:

|  | $L_{k}=\frac{1}{2}\sum_{r,p} B_{rp}\left( 1+\log\left( s_{\left[ k \right],p}^{2} \right) \right)\geq\frac{1}{2}\sum_{r,p} B_{rp}\left( 1+\log s_{\min}^{2} \right)\equiv L_{\min} .$ | (N2.15) |
| --- | --- | --- |

The monotone convergence theorem, together with the lower bound (N2.15), guarantees that the sequence $\{L_{k}\}$ will converge to a finite value, which corresponds to a maximum of the posterior distribution. In practice, we consider that convergence is attained when ${(L_{k+1}-L_{k})}/{|L_{k}|}$ is less than a threshold value $T={10}^{-10}$ (note that numerical precision prevents lowering $T$ below ${10}^{-15}$). The sequence is usually observed to reach the tolerance within 10 to 20 iterations (Fig. N2.3A-B). In order to guarantee that the minimum of $L$ does indeed correspond to a global minimum, the whole procedure is iterated 10 times starting from different (random) initial peptide uncertainties  $\vec{\sigma_{\left[ 0 \right]}^{2}}$, and the MAP estimators that lead to the smallest $L$ are selected. In the vast majority of cases, the same optimal solution is obtained in all 10 cases, and only in a handful of cases the optimization procedure leads to multiple minima (Fig. N2.3C).


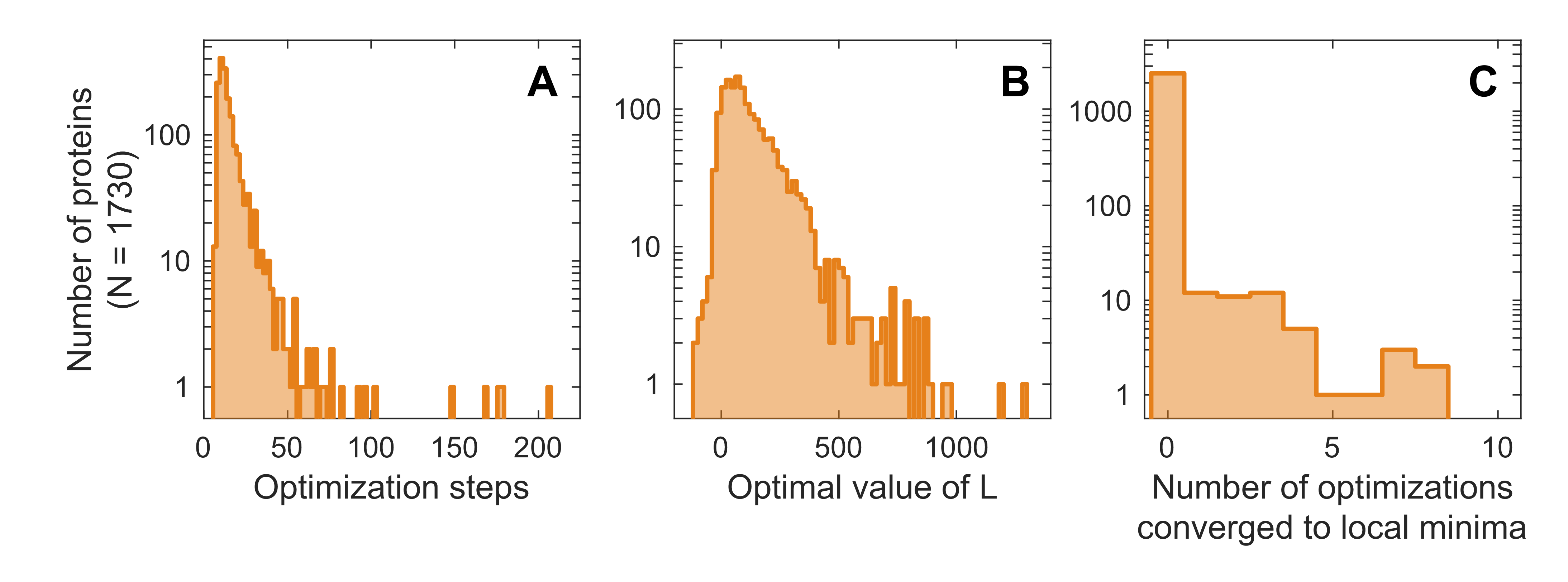


**Figure N2.3. Convergence analysis of the optimization algorithm. (A)** Number of optimization steps needed to reach convergence for the protein abundances in 30 samples from Dataset EV2. Convergence is reached as $\left| L_{k+1}-L_{k} \right|<T\left| L_{k} \right|$, with a threshold $T={10}^{-10}$. The average number of steps to reach convergence is 15.7. **(B)** Distribution of the optimal values of $L$ after convergence. **(C)** For each protein, the optimization procedure was performed 10 times starting from random initial peptide uncertainties. From those, we computed for each protein the number of times the optimal value of $L$ was significantly larger than the minimum value $L_{\min}$ obtained across the 10 iterations, thus identifying a local minimum; in concrete terms, we computed the number of times that $\left( L-L_{\min} \right)>10\cdot T\cdot\left| L_{\min} \right|$, where $T={10}^{-10}$ is the threshold associated to convergence. In the vast majority of cases, all optimizations converge to the same value $L_{\min}$ within the convergence tolerance; if multiple minima are found, the one with the smallest $L$ (equal to $L_{\min}$) is chosen.

The estimated xTop protein intensities can be read off the optimal value of the vector $\vec{x}$. Furthermore, as an additional output, we also obtain the detection efficiencies $\varepsilon_{p}$ for each peptide, as well as the variance $\sigma_{p}^{2}$ in the log-transformed intensity for each peptide precursor. In principle, the posterior probability distribution also encodes for the uncertainties on the fitted intensities and efficiencies, whose determination is an important but challenging problem on its own. While it would be desirable to extend xTop to include proper estimates of confidence intervals for the protein abundances, they would depend directly on the value of the parameter $\sigma_{\min}^{2}$ (see below), so we do not feel confident in interpreting them as proper error bars for the protein intensities. Instead, the inferred values for $\sigma_{p}^{2}$ are to be interpreted as (inverse) weights that modulate how much each peptide precursor affects the estimation of the xTop protein intensity: for instance, if two peptides $p_{1}$ and $p_{2}$ have inferred variances $\sigma_{p_{1}}^{2}>\sigma_{p_{2}}^{2}$, then $p_{2}$ will contribute more than $p_{1}$ to the definition of the xTop intensity (by a factor ${\sigma_{p_{2}}^{2}}/{\sigma_{p_{1}}^{2}}>1$ ).

## On the prior for the standard deviations and the parameter $\boldsymbol{\sigma}_{\mathbf{min}}^{\mathbf{2}}$

In this section we discuss the behavior of the xTop solution for different values of the parameter $\sigma_{\min}^{2}$ setting used to model the peptide scatter. The prior term $p_{\mathrm{prior}}^{(3)}$ consists in the product of exponential terms of the form $\exp(-\sigma_{\min}^{2}/\sigma_{r}^{2})$. As $\sigma_{r}^{2}$ is reduced below $\sigma_{\min}^{2}$, the exponent becomes large and negative, thus suppressing both $p_{\mathrm{prior}}^{(3)}$ and $p_{\mathrm{post}}$. Its effect on the variances maximizing the a posteriori probability can be directly seen in Eq. (1.14): the value $\sigma_{\min}^{2}$ sets the minimum variance associated to each peptide *p*. This means that $\sigma_{\min}^{2}$ can potentially have a strong effect on the estimated xTop intensities.

We tested the behavior of the MAP solutions for different values of $\sigma_{\min}^{2}$ on several simulated datasets, which allow to compare the inferred values of $\sigma_{p}^{2}$ to the “true” values from which the data has been generated; a representative example of these simulations can be seen in Fig. N2.4, in which we compare the true peptide variances (green circles, ranging between $0.01$ and 0.5) to the inferred ones for different values of $\sigma_{\min}^{2}$. When $\sigma_{\min}^{2}$is much smaller than the true peptide variances, the optimization converge to a set of parameters in which only one of the peptide precursors ($p^{*}$) has a very small inferred variance, $\sigma_{p^{*}}^{2}\approx\sigma_{\min}^{2}$ (blue line in Fig. N2.3, corresponding to ${\sigma_{\min}^{2}=10}^{-4}$). In this case, the contributions of the other peptide precursors are suppressed by a factor of the order ${\sigma_{p}^{2}}/{\sigma_{\min}^{2}}\gg1$, and the xTop estimator $I_{s}^{\mathrm{xTop}}$ is approximately given by the product of the intensity of this peptide precursor divided by its inferred detection efficiency, i.e. $I_{s}^{\mathrm{xTop}}\approx{I_{p^{*}s}}/{\varepsilon_{p*}}$. The opposite case is that of $\sigma_{\min}^{2}$ being much larger than the true variance of the peptide intensities (yellow line in Fig. N2.3, corresponding to $\sigma_{\min}^{2}=1$). In this case, all peptide precursors contribute about the same to the definition of the xTop intensity, regardless on their true scatter.

In the range between these two extreme cases, the inferred peptide variances, and therefore contribution of each peptide to the definition of the xTop intensities, depend on both the true variances and the value of $\sigma_{\min}^{2}$. When the value of $\sigma_{\min}^{2}$ is comparable to that of the true variances peptides (e.g. the red line in Fig. N2.4 corresponding to ${\sigma_{\min}^{2}=10}^{-2}$), the MAP estimators $\sigma_{p}^{2}$ appear to be strongly correlated with the true variances. Lower values of $\sigma_{\min}^{2}$ reduce the inferred variance of the most consistent peptides, and hence increase their weight in the determination of the protein intensity. Vice versa, increasing the minimum variance $\sigma_{\min}^{2}$ reduces the spread of $\sigma_{p}^{2}$ between the most and least consistent peptides, thus weighing the different peptides more equally.


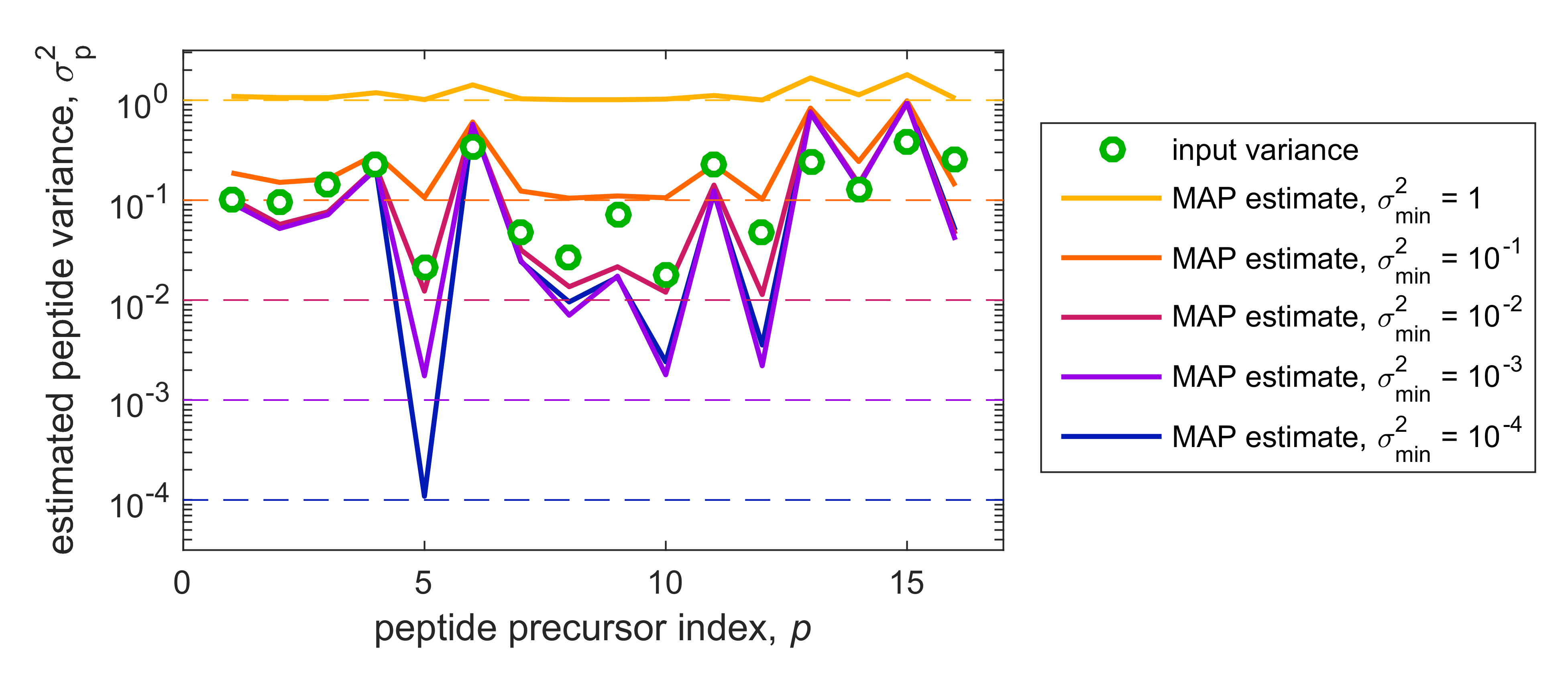


### Figure N2.4. Impact of $\boldsymbol{\sigma}_{\mathbf{min}}^{\boldsymbol{2}}$on the inferred peptide-specific variances. This example illustrates the role of the parameter $\boldsymbol{\sigma}_{\mathbf{min}}^{\boldsymbol{2}}$ on the estimated values of $\boldsymbol{\sigma}_{\boldsymbol{p}}^{\boldsymbol{2}}$ that determine how strongly each peptide $\boldsymbol{p}$ affects the xTop intensity (peptides with lower variance are weighted more). The xTop pipeline was run on a simulated set of 16 peptides across 30 samples with various detection efficiencies and peptide-specific scatter (indicated by the green circles), plus additional noise, for various values of $\boldsymbol{\sigma}_{\mathbf{min}}^{\boldsymbol{2}}$. If $\boldsymbol{\sigma}_{\mathbf{min}}^{\boldsymbol{2}}$ is much larger than the true peptide variances, all peptides are weighted roughly equally. In the opposite case, with $\boldsymbol{\sigma}_{\mathbf{min}}^{\boldsymbol{2}}$ smaller than all true variances, one of the peptide is weighted much more than the others, since it has a much smaller variance close to $\boldsymbol{\sigma}_{\mathbf{min}}^{\boldsymbol{2}}$. xTop reproduces the true variances the best when $\boldsymbol{\sigma}_{\mathbf{min}}^{\boldsymbol{2}}$ is close to the variance of the most precise peptides, which in this example is about $\boldsymbol{10}^{\boldsymbol{-2}}$.

### Estimating the best value for $\boldsymbol{\sigma}_{\mathbf{min}}^{\boldsymbol{2}}$

In summary, the parameter $\sigma_{\min}^{2}$ acts as a “knob” which modulates how much xTop relies on the inferred scatter of the peptide precursors to estimate the xTop intensities. This behavior ranges between picking only the most consistent peptide precursor (for small $\sigma_{\min}^{2}$) to combining all peptide intensities. Each extreme has its own potential advantages: using a small value of $\sigma_{\min}^{2}$ makes use of the “best” peptides, while using a large values might potentially protect against systematic errors such as peptide misidentification. The value of $\sigma_{\min}^{2}$ that optimizes the xTop pipeline depends on the precision of the peptide precursors, and has to be determined empirically. To do so, we compared the scatter between log-transformed protein intensities from pairs of replicate samples. Figure N2.5 shows the variance of the log-ratio of the xTop intensities across two replicates, $\log_{10} I_{p}^{(1)}/I_{p}^{(2)}$, across the proteome. The protein intensities were computed using several values of $\sigma_{\min}^{2}$, leading to varying variance (purple circles).

The variance of the log10-transformed ratios of the protein intensities obtained from TopPep1/3 and iBAQ is between $2.8\cdot{10}^{-3}$ and 1.5$\cdot{10}^{-2}$ (solid lines in Fig. N2.5); we therefore expect the best results to be obtained with a value for $\sigma_{\min}^{2}$ of comparable magnitude. When varying $\sigma_{\min}^{2}$ by four orders of magnitude (from ${10}^{-4}$ to 1), we found that xTop outperforms TopPep1/3 and iBAQ for all values of $\sigma_{\min}^{2}$ across the whole range; the best performance (lowest scatter) is found for $\sigma_{\min}^{2}\approx{10}^{-2}$, which is the value we picked for all our results. Interestingly, the performance of xTop is largely independent on the value of $\sigma_{\min}^{2}$. In fact, most of the scatter in TopPep3 and iBAQ originates from missing peptides, which are accounted for by xTop for all values of the parameter $\sigma_{\min}^{2}$.


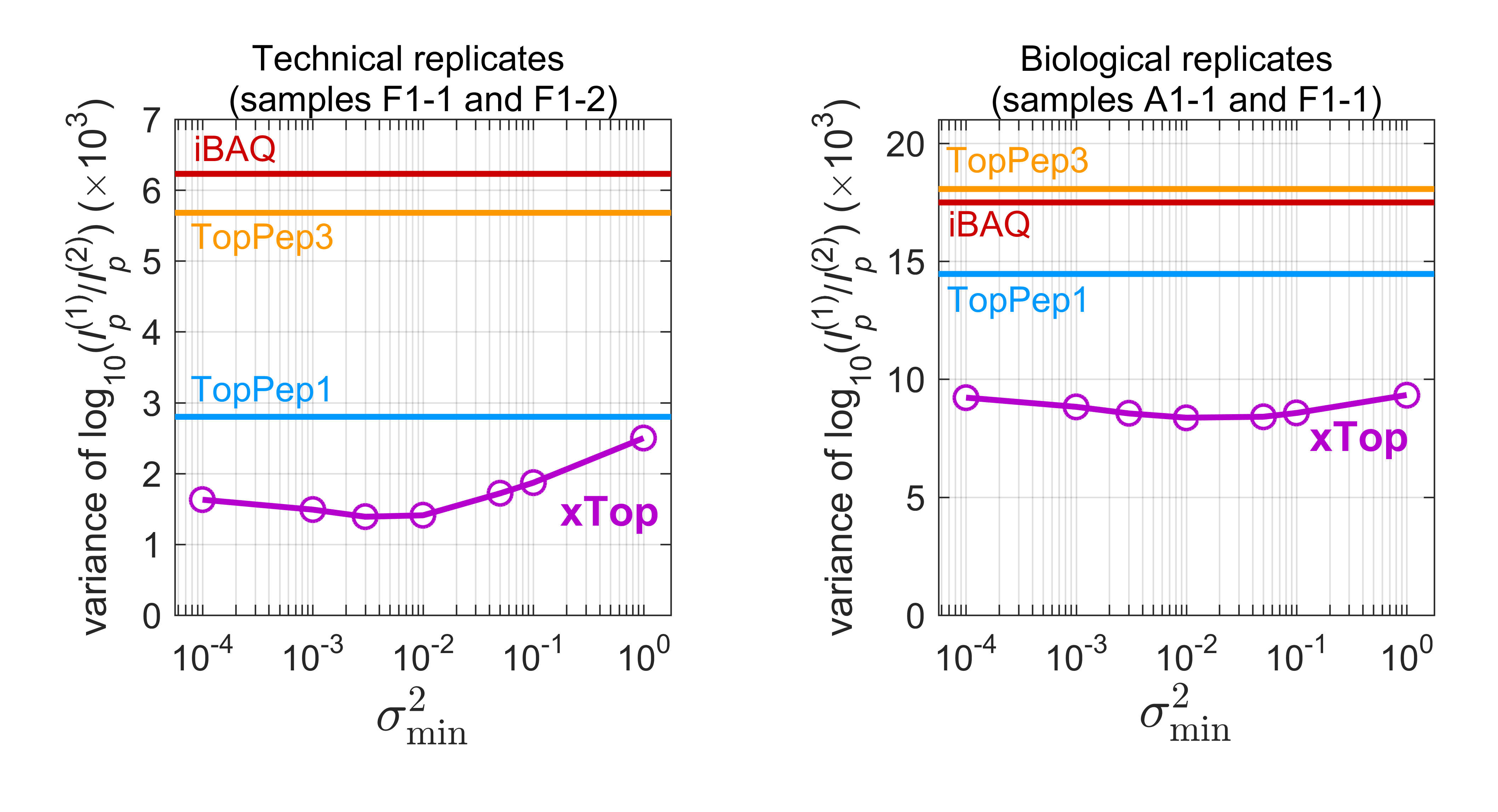


**Figure N2.5. Scatter in protein intensities for different values of** $\boldsymbol{\sigma}_{\mathbf{min}}^{\boldsymbol{2}}$**.** We studied the scatter in the protein intensities we considered protein intensities $I_{p}^{(1)}$ and $I_{p}^{(2)}$ corresponding to either two technical replicates (left panel, samples F1-1 and F1-2) or biological replicates (right panel, samples A1-1 and F1-1) and computed the variance of their log-trasformed ratio, $\mathrm{Var}\left( \log_{10} {I_{p}^{(1)}}/{I_{p}^{(2)}} \right)$ (the same quantity indicated in Fig. 2H and Appendix Figure S2B, S2E). This was done for both xTop protein intensities obtained for several values of the parameter $\sigma_{\min}^{2}$ (purple circles) and for TopPep1, TopPep3 and iBAQ protein intensities (horizontal lines). xTop is seen to outperform all other three approaches for all values of $\sigma_{\min}^{2}$ between ${10}^{-4}$ and 1; the performance depends only weakly on the value of $\sigma_{\min}^{2}$, only worsening significantly for large $\sigma_{\min}^{2}$ in the case of technical replicates (left). The best results are obtained using $\sigma_{\min}^{2}\approx{10}^{-2}$, which is the value used for all main results in this work. Note that the variance reported on the y-axis depends only on the ratio of protein intensities, and is therefore not affected by overall variations in protein intensities across different methods (e.g. TopPepN yielding numerically larger protein intensities than TopPep1).

# Note S3: Quantitative analysis of bias in protein intensities

In this note we will study the quality of the absolute quantification of xTop, TopPep1, TopPep3 and iBAQ approaches in absolute terms, by studying the correlations of the protein intensities to the Riboseq protein abundances, protein lengths and number of detected peptide precursors. In this section we will use mass fractions, which we indicate with $\phi_{i}$ for mass spec and $\rho_{i}$ for ribosome profiling. First, we define the log-transformed quantities:

|  | $w_{i}^{(\alpha)}=\log_{10} \phi_{i}^{(\alpha)}, z_{i}=\log_{10} \rho_{i}$ | (N3.1) |
| --- | --- | --- |

where $\alpha$ stands for either TopPep1, TopPep3, iBAQ or xTop. Their differences $x_{i}^{(\alpha)}$ are the log-transformed ratios of the mass fractions, shown e.g. in Appendix Fig. S4D-G:

|  | $x_{i}^{(\alpha)}=w_{i}^{(\alpha)}-z_{i}=\log_{10} {\phi_{i}^{\left( \alpha\right)}}/{\rho_{i}} .$ | (N3.2) |
| --- | --- | --- |

If there were a perfect match between mass spec and ribosome profiling-derived mass fractions, all log-ratios $x_{i}^{(\alpha)}$ would be zero. In reality, the values of the $x_{i}^{(\alpha)}$ depend on both random scatter (noise in both mass spectrometry and ribosome profiling measurements) and on systematic biases.

As a measure of spread, we consider the variance of the log-ratios, $\sigma_{x}^{2}$, as well as the standard deviation $\sigma_{x}$, and its associated fold-error ${10}^{\sigma_{x}}$ in the ratio $\phi_{i}/\rho_{i}$, i.e. the typical fold-distance between mass spec- and ribosome profiling-quantified protein mass fractions. To give an idea of the magnitude of the errors, consider that $\sigma_{r}= 0.2,$0.3 and 0.4 correspond to 1.6, 2 and 2.5-fold deviations in the residuals, respectively.

We report in Table N3.1 the variances, standard deviations and corresponding fold changes of $x_{i}^{(\alpha)}$ for the four protein quantification methods in a representative calibration sample (F1-1). Variances are also shown in Fig. 3F as a bar chart. We see that variances associated to iBAQ and xTop are larger than those TopPep1 and xTop. As noted in the Main Text, TopPep3 and iBAQ mass fractions present some nonlinear behavior, with an apparent underestimation of protein abundances for $\phi_{i}\lesssim{10}^{-4}$. This nonlinearity is reflected into a larger scatter of the residuals, $\sigma_{r}$, and a larger correlation $R(r_{i},w_{i})$ between the log-ratios and the log-transformed protein mass fractions, compared to that of TopPep1 and xTop. On the other hand, iBAQ is much less biased in terms of protein size than TopPep1/3 and xTop, as indicated by the almost vanishing correlation with the log-transformed protein sizes, $R(r_{i},f_{i}^{L})$; instead, all other methods correlate strongly with the log-transformed protein lengths, as also visually apparent in Appendix Figure S4D-G.

|  | Method | Variance $\left( \sigma_{x}^{2} \right)$ | Standard deviation $\left( \sigma_{x} \right)$ | Fold change  $\left( {10}^{\sigma_{x}} \right)$ | correlation coefficient | |
| --- | --- | --- | --- | --- | --- | --- |
|  |  |  |  |  | $R\left( x_{i},w_{i}^{\left( \alpha\right)} \right)$ | $R\left( x_{i},\log_{10} l_{i} \right)$ |
| Statistics of  $x_{i}=\log_{10} \phi_{i}^{\left( \alpha\right)}/\rho_{i}$ | TopPep1 | 0.114 | 0.338 | 2.18 | 0.236 | 0.443 |
|  | TopPep3 | 0.147 | 0.384 | 2.42 | 0.488 | 0.491 |
|  | iBAQ | 0.139 | 0.372 | 2.35 | 0.585 | 0.022 |
|  | xTop | 0.113 | 0.336 | 2.17 | 0.221 | 0.453 |

**Table N3.1.** Statistics of the log-ratio between mass spectrometry and ribosome profiling-based protein mass fractions.

## Multivariate analysis

To further disentangle the possible biases in the log-ratios $x_{i}^{(\alpha)}=\log_{10} \phi_{i}^{(\alpha)}/\rho_{i}$, we fit them with a family of linear models of the form $M_{i}=c_{0}+\sum_{k} c_{k}f_{i}^{k}$, where the vectors $f_{i}^{k}$ are the “features” and $c\equiv\{c_{k}\}$ is a set of parameters, one per feature plus an overall constant $c_{0}$, which tune the impact of each feature on the ratios $x_{i}$. After optimizing the parameters $c_{k}$ to the values $\bar{c}_{k}^{\alpha}$ minimizing the sum of squared differences, $\sum_{i} \left( x_{i}^{(\alpha)}-M_{i} \right)^{2}$, it is possible to compute the residuals $r_{i}^{(\alpha)}=x_{i}^{(\alpha)}-\bar{M}_{i}^{(\alpha)}$ as well as the associated statistics. The variance of the components $\bar{c}_{k}^{\alpha}f_{i}^{k}$ represent the components of variance “explained” by the features $f_{i}^{k}$ (shown as colored bars in Fig. 3F); furthermore, the correlation coefficient of the residuals with the features give us information on the biases that could not be removed by the fit, and hence on the simultaneous presence of different sources of bias. We considered models based upon the following features:

- The first feature is the log-transformed protein length $f_{i}^{L}=\log_{10} l_{i}$, with $l_{i}$ being the number of residues of i-th protein. Note that this feature does not depend on the sample, i.e. it is condition-independent.
- Secondly, we considered two features based on the number of distinct peptide precursors observed in the condition at hand, $n_{i}$. We considered $f_{i}^{1}=\delta(n_{i},1)$ and $f_{i}^{2}=\delta(n_{i},2)$, where $\delta(\cdot,\cdot)$ is the Kronecker delta ($\delta\left( A,B \right)=1$ if A=B, and 0 otherwise).

We focus a single representative sample (F1-1), only considering the proteins for which $\rho_{i}\geq{5\cdot10}^{-6}$ to remove a few points with very low protein abundance that might skew the correlations. We end up with a set of $1779$ proteins for which both ribosome profiling and mass spectrometry data is available. The performances of the different protein quantification approaches, as well as the improvements obtained by taking into consideration additional features, are summarized in Table N3.2, displayed in Figures N3.1 and N3.2, and described in the rest of the note.

### Correction for proteins with 1 or 2 peptide precursors

First, we want to determine the contribution to the residuals of the proteins with either one or two peptide precursors to the overall scatter and to the other biases. To do so, we fit the differences $x_{i}^{\left( \alpha\right)}$ with a three-parameter model:

| (Model #1) | $M_{i}=c_{0}+c_{1}f_{i}^{1}+c_{2}f_{i}^{2} .$ | (N3.3) |
| --- | --- | --- |

Here, $\delta_{i}^{1}$ and $\delta_{i}^{2}$ shift rigidly the ratios of all proteins with 1 or 2 peptide precursors by a constant $c_{1}$ or $c_{2}$, respectively. As we can see in Table N3.2, the correlation of the residuals with the protein intensity, $R(r_{i},w_{i})$, is greatly reduced for TopPep3, becoming comparable to the ones of TopPep1 and xTop. A similar reduction is also observed for iBAQ. The correlations with the protein lengths are not qualitatively different, with a further reduction in $R(r_{i},f_{i}^{L})$ for iBAQ only.

### Protein size bias

We then focused on the bias on protein size observed for TopPep1/3 and xTop. We thus checked if a simple linear model is able to remove a large fraction of the bias:

| (Model #2) | $M_{i}=c_{0}+c_{L}f_{i}^{L} .$ | (N3.4) |
| --- | --- | --- |

As can be seen by comparing Table N3.1 and Table N3.2, the scatter in the residues, as measured by $\sigma_{r}$, is strongly reduced for both TopPep1/3 and xTop using a value $c_{L}\approx0.6$. Interestingly, the correlation with the protein abundance, $R(r_{i},w_{i})$, is also strongly reduced, suggesting that the nonlinearities hinted by the correlation of the log-ratios $x_{i}$ with the protein abundances $w_{i}$ were actually an artifact produced by the protein size bias. Instead, the iBAQ intensities show little improvement, consistently with the fact that they were not correlated with the protein sizes in the first place. We note that the number of fully tryptic enzymes correlates strongly with the protein size, which accounts for iBAQ not being strongly biased with respect to protein size.

**Combining peptide precursor number and protein size biases**

Finally, we combined the two models studied above, thus accounting for both the number of detected peptides and the protein size, as well as the interactions between the two:

| (Model #3) | $M_{i}=c_{0}+c_{1}f_{i}^{1}+c_{2}f_{i}^{2}+c_{L}f_{i}^{L}$ | (N3.5) |
| --- | --- | --- |

In this case, the only protein quantification method that displays a significant improvement with respect to the previous two linear models is TopPep3, signaling that the accuracy of this method is affected by both peptide quantification and protein size. Indeed, both effects explain a sizable component of the variance. It is important to note that the best-fit values for the coefficients $c_{k}$ did not change significantly compared to Models #1 and #2, implying that effects of the features on the log-ratios $x_{i}$ are mostly independent.

| **Model** | **Quant. method** | **Best-fit parameters** | | | | **Explained variance** | | | **Statistics of fit residuals** $\boldsymbol{r}_{\boldsymbol{i}}$ | | | | |
| --- | --- | --- | --- | --- | --- | --- | --- | --- | --- | --- | --- | --- | --- |
|  |  | $\boldsymbol{c}_{\boldsymbol{0}}$ | $\boldsymbol{c}_{\boldsymbol{L}}$ | $\boldsymbol{c}_{\boldsymbol{1}}$ | $\boldsymbol{c}_{\boldsymbol{2}}$ | $\boldsymbol{f}_{\boldsymbol{i}}^{\boldsymbol{L}}$ | $\boldsymbol{f}_{\boldsymbol{i}}^{\boldsymbol{1}}$ | $\boldsymbol{f}_{\boldsymbol{i}}^{\boldsymbol{2}}$ | $\boldsymbol{\sigma}_{\boldsymbol{r}}^{\boldsymbol{2}}$ | $\boldsymbol{\sigma}_{\boldsymbol{r}}$ | $\boldsymbol{10}^{\boldsymbol{\sigma}_{\boldsymbol{r}}}$ | $R\left( r_{i},w_{i}^{\left( \alpha\right)} \right)$ | $R\left( r_{i},f_{i}^{L} \right)$ |
| Model #1  ($f_{i}^{1}$, $f_{i}^{2}$) | **TopPep1** | 0.0891 | *NA* | -0.0986 | -0.0217 | *NA* | 0.0013 | <10^-4^ | 0.113 | 0.336 | 2.17 | 0.181 | 0.433 |
|  | **TopPep3** | 0.0768 | *NA* | -0.4647 | -0.2021 | *NA* | 0.0299 | 0.0044 | 0.117 | 0.342 | 2.20 | 0.202 | 0.475 |
|  | **iBAQ** | 0.0320 | *NA* | -0.5157 | -0.2525 | *NA* | 0.0373 | 0.0068 | 0.100 | 0.316 | 2.07 | 0.261 | -0.050 |
|  | **xTop** | 0.0958 | *NA* | -0.0846 | -0.0145 | *NA* | 0.0010 | <10^-4^ | 0.112 | 0.335 | 2.16 | 0.185 | 0.447 |
| Model #2  $\left( f_{i}^{L} \right)$ | **TopPep1** | -1.3003 | 0.5612 | *NA* | *NA* | 0.0225 | *NA* | *NA* | 0.092 | 0.303 | 2.01 | 0.069 | 0.000 |
|  | **TopPep3** | -1.7551 | 0.7071 | *NA* | *NA* | 0.0356 | *NA* | *NA* | 0.112 | 0.334 | 2.16 | 0.334 | 0.000 |
|  | **iBAQ** | -0.1636 | 0.0318 | *NA* | *NA* | 0.0001 | *NA* | *NA* | 0.139 | 0.373 | 2.36 | 0.560 | 0.000 |
|  | **xTop** | -1.3203 | 0.5722 | *NA* | *NA* | 0.0233 | *NA* | *NA* | 0.090 | 0.300 | 2.00 | 0.044 | 0.000 |
| Model #3  $\left( f_{i}^{1},f_{i}^{2},f_{i}^{L} \right)$ | **TopPep1** | -1.3067 | 0.5634 | -0.0343 | 0.0511 | 0.0227 | 0.0002 | 0.0003 | 0.091 | 0.302 | 2.00 | 0.069 | 0.000 |
|  | **TopPep3** | -1.4667 | 0.6228 | -0.4020 | -0.1259 | 0.0276 | 0.0224 | 0.0017 | 0.090 | 0.300 | 2.00 | 0.093 | 0.000 |
|  | **iBAQ** | 0.2514 | -0.0885 | -0.5240 | -0.2633 | 0.0006 | 0.0385 | 0.0074 | 0.100 | 0.316 | 2.07 | 0.262 | 0.000 |
|  | **xTop** | -1.3336 | 0.5767 | 0.0269 | 0.0560 | 0.0237 | 0.0001 | 0.0003 | 0.089 | 0.299 | 1.99 | 0.066 | 0.000 |

**Table N3.2.** Best fit parameters and statistics of the residuals $r_{i}$ for the models shown in Table N1 and discussed in the note, for the four protein quantification methods TopPep1/3, iBAQ and xTop. Model #1 includes two terms shifting all values of $x_{i}$ for the proteins with one or two peptide precursors detected; Model #2 is a linear function of the log-transformed protein size; Model #3 combines both previous models. The residuals of the fits are shown in Figures N3.1 and N3.2. The explained variances for Model #3 are shown in Main Text Figure 2F as colored bars. Note that the correlation between residuals and the log-transformed protein size $f_{i}^{L}$ for models #2 and #3 is zero by construction.


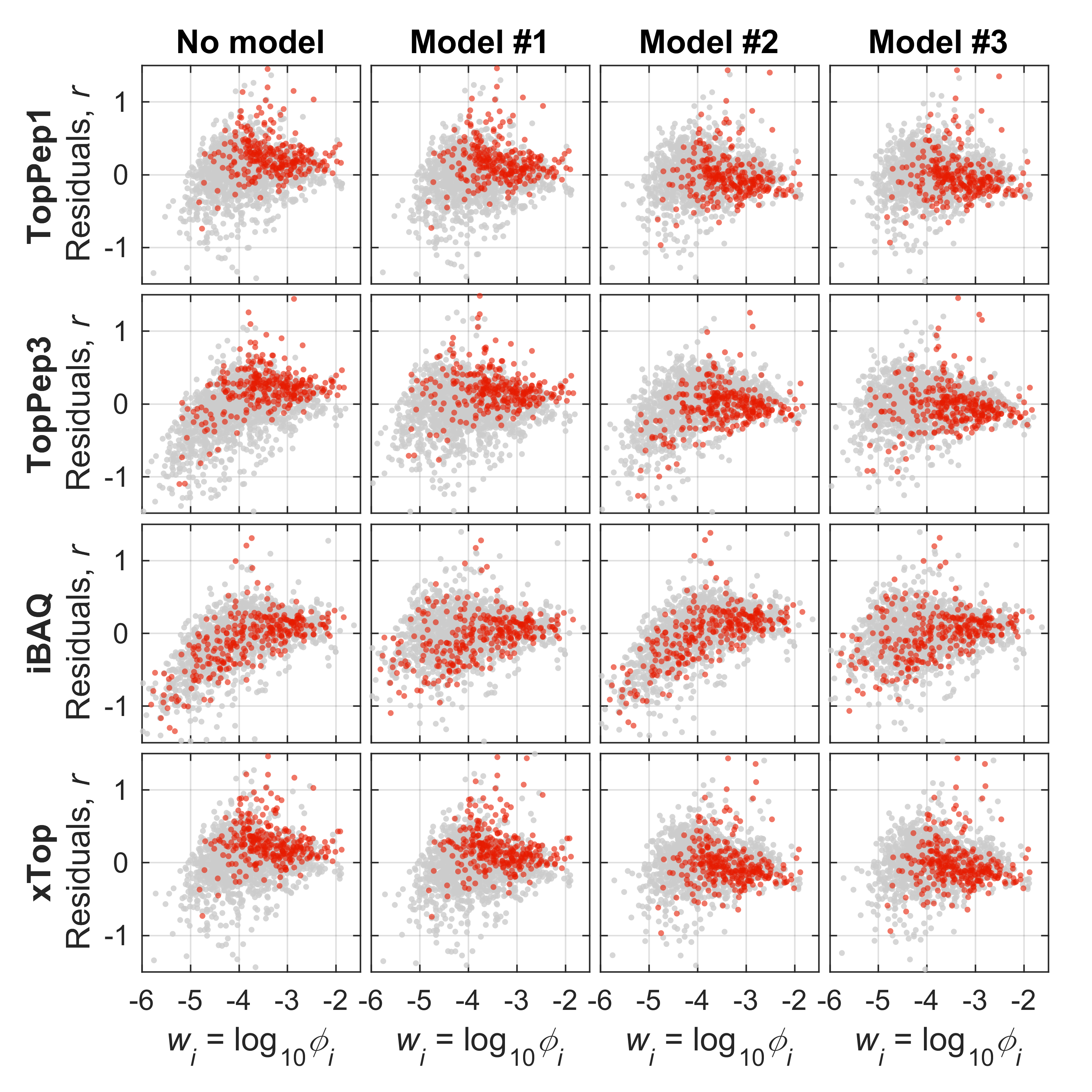


**Fig N3.1.** Fit results for the models. Large proteins with at least 500 residues are highlighted in red. For these proteins, the TopPep1 and TopPep3 residuals are weakly correlated with ribosome profiling mass fractions, while the strong correlation between iBAQ residuals and Riboseq mass fractions $\rho_{i}$ cannot be completely removed.


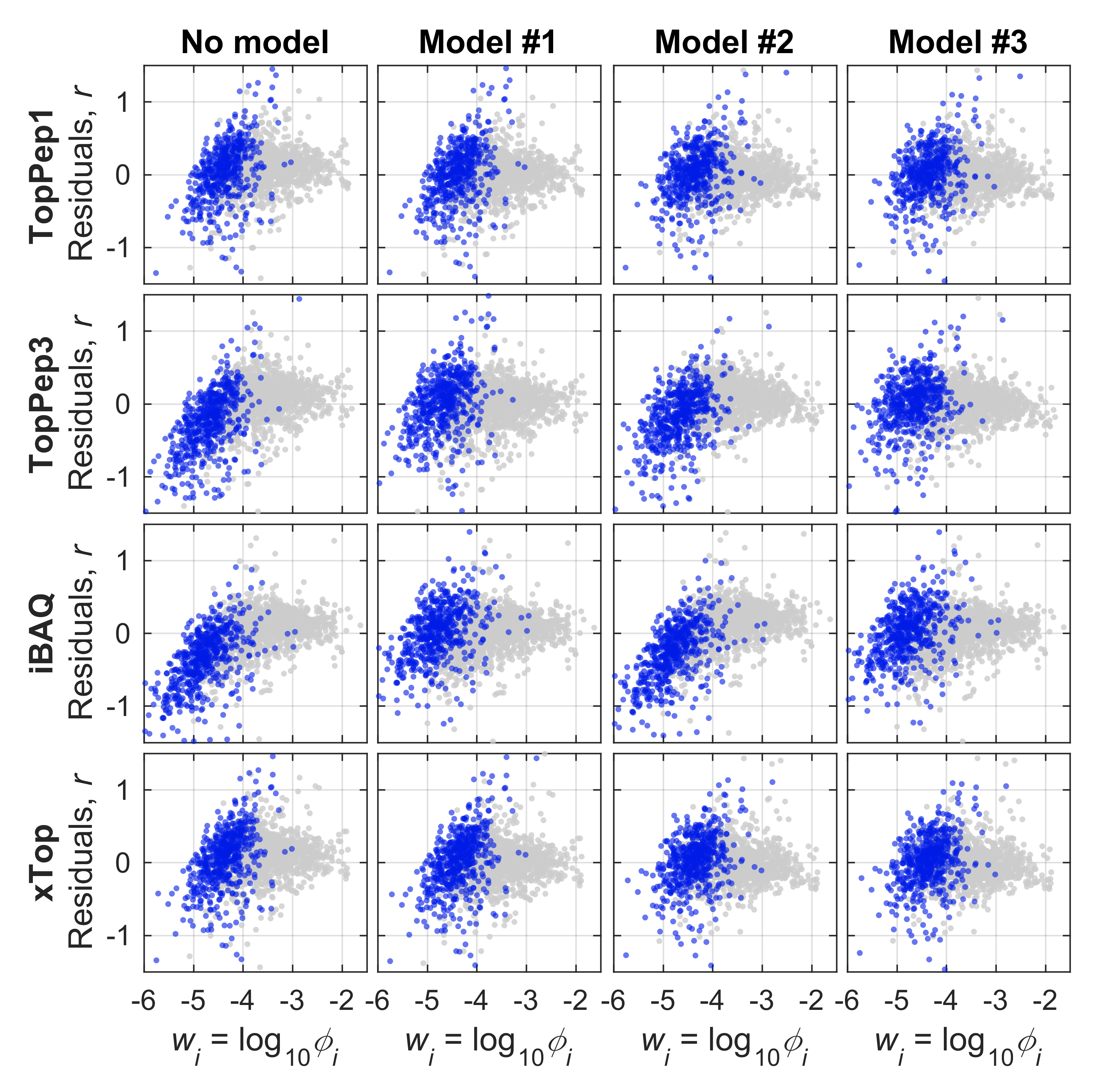


**Fig N3.2.** Fit results for the models. Protein for which less than 3 peptides has been detected in this condition are highlighted in blue. TopPep3 and iBAQ tend to yield negative residuals for these proteins (panels on the left). The models M1 and M3, allowing to increase the intensity of the proteins with one or two peptides detected, allows to shift the residues so that the average residue is zero, but is not able to improve the spread in the data.

**Bias removal via calibration of protein abundances**

The strategy employed in the “versatile workflow” described in the Main Text includes a scaling using some “calibration” absolute protein mass fractions obtained via ribosome profiling. Here we indicate the mass fraction of protein $k$ in sample $s$ as $\phi_{k,s}$. The calibration is performed by multiplying the protein mass fractions $\phi_{k,s}$ by a condition-independent scaling factor $c_{k}$, and then normalizing the result to unity:

|  | $\phi_{k,s}\to\frac{\phi_{k,s}c_{k}}{\sum_{j} \phi_{j,s}c_{j}}$ | (N3.6) |
| --- | --- | --- |

In the case of ribosome profiling calibration described in the Main Text, the scaling factors $c_{k}$ are the ratio of ribosome profiling-derived and mass spectrometry (xTop) derived protein mass fractions in the calibration samples, $c_{k}=\rho_{k,\mathrm{cal}}/\phi_{k,\mathrm{cal}}$. Since these factors average around 1, the denominator $\sum_{j} \phi_{j,s}c_{k}$ in Eq. N3.6 is in practice close to unity, so the action of the calibration on individual proteins is to rescale by a constant all mass fractions across the samples, $\phi_{k,s}\to c_{k}\phi_{k,s}$.

The result of the analysis presented in this note suggest however an alternative scaling that can potentially improve the quality of absolute protein quantification in absence of such calibration samples. Such scaling factors can be easily obtained with the best fit parameters shown in Table N3.2. For instance, the protein size $L_{k}$ is among the parameters that can be used to calibrate the protein mass fractions. According to the results of model #2 discussed in the previous section, a value $c_{L}\sim0.57$ removes the correlation of $x_{i}=\log_{10} \rho_{i}/\phi_{i}$ to the log-transformed protein lengths $f_{i}^{L}=\log_{10} L_{i}$ for the xTop method. This implies that the quantity $x_{i}^{'}=\log_{10} \phi_{i}L_{i}^{-c_{L}}/\rho_{i}$ is not correlated with $f_{i}^{L}$. This suggests to recalibrate the protein mass fractions using $L_{i}^{-c_{L}}$ as scaling factor as follows:

|  | $\phi_{k,s}\to\frac{\phi_{k,s}L_{k}^{-0.57}}{\sum_{j} \phi_{j,s}L_{j}^{-0.57}} .$ | (N3.7) |
| --- | --- | --- |

By construction, the resulting protein mass fractions are completely unbiased towards protein size, and show reduced scatter when used to compute the log-ratio with ribosome profiling-derived mass fractions (shown in Fig. N3.1 and N3.2). This is a viable approach that can be used to improve the absolute quantitation when “reference” absolute protein abundances (e.g. from ribosome profiling) are not available.

In the particular case of a protein size-dependent scaling of xTop protein intensities, the variance in the log-ratio after the scaling is reduced from 0.112 to 0.090, i.e. a 20% reduction, which translates in a reduction of the typical fold-distance (${10}^{\sigma_{x}}$) between mass spec- and ribosome profiling-based protein mass fractions from 2.16 to 2-fold. While the reduction in the overall spread does not seem large, the scaling has a strong effect on the estimated protein abundances. Consider two proteins that have very different sizes, e.g. $L_{1}=500$ residues and $L_{2}$ = 100 residues. If the two proteins are quantified by xTop to have the same protein mass fraction, after the scaling the estimated protein mass fraction for the small protein would be a factor $5^{0.57}=2.5$ larger compared to the large protein. The magnitude of this effect is also clearly visible in Figure N3.1 as a downward shift in the abundance of large proteins with more than 500 residues (red). In conclusion, while this scaling method can be used to remove a striking bias in the absolute quantitation provided by the xTop method, the relative minor reduction in the spread of the residues suggest that other sources of bias are likely to be present, either at the protein level or at the peptide precursor level.

# Note S4: GO-term enrichment analysis

We performed a GO-term enrichment on each one of the eight protein sectors defined along the lines of Hui *et al.* (Hui *et al.*, 2015), as follows. First, a list of GO-terms for all biological processes, and the list of GO-terms associated to each individual gene, were downloaded from Ecocyc (Keseler *et al*, 2017). From this list, we generated a list of unique GO-terms, removing all terms which are associated to less with less than 3 genes, but have a parent term associated to 3 or more genes. We ended up with a matrix $M_{it}$ whose entries are 1 if the *i*-th gene is associated to the *t*-th GO-term, and zero otherwise.

When performing the enrichment analysis, we wanted to highlight the contribution from the most abundant proteins. To do so, it is necessary to weigh the frequency of the GO-terms by some measure of protein abundance. We found results to be largely independent on the specific choice of these weights $w_{i}$. The results presented in this manuscript are obtained according to the following procedure:

1. We denote $\phi_{i}$ the arithmetic mean of the protein mass fraction in reference condition and in each one of the three extreme limitation conditions (C-, A- and R-limitation, with slowest growth), i.e. $\phi_{i}=\left( \phi_{i}^{\mathrm{ref}}+\phi_{i}^{C-lim}+\phi_{i}^{A-lim}+\phi_{i}^{R-lim} \right)/4$.
2. For each gene, we define $f_{i}=6+\log_{10} \phi_{i}$ if $\phi_{i}\geq{10}^{-6}$, and $f_{i}=0$ otherwise (note that $f_{i}\geq0$). This choice is slightly different from what had been used in Hui et al. 2015, where the weights were proportional to the protein mass fraction $\phi_{i}$.
3. Then, for each protein sector X we define the weights $w_{X,i}\equiv f_{i}/\sum_{j\in\{X\}} f_{j}$, where $\{X\}$ indicates the set of all genes included in X. These weights are non-negative numbers in the range 0 (for low abundant proteins at or below our detection limit) to 1 (if the set X consists of only one protein), linearly related to the log-transformed protein mass fraction.
4. Finally, we ranked the GO-terms *t* by sorting in decreasing order the following score:

$$r(X,t)=\sum_{i\in\left\{ X \right\}} w_{X,i}\cdot M_{it}$$

The score ranges from 0 if the GO-term is not represented in X, to 1 if all genes in the group are associated to the same GO-term. In fact, for each group X, the maximum value of the score across all GO-terms is a measure of the degree of specialization of X: the higher the score, the more likely are the genes to share the same biological function.

# Appendix Figures

**
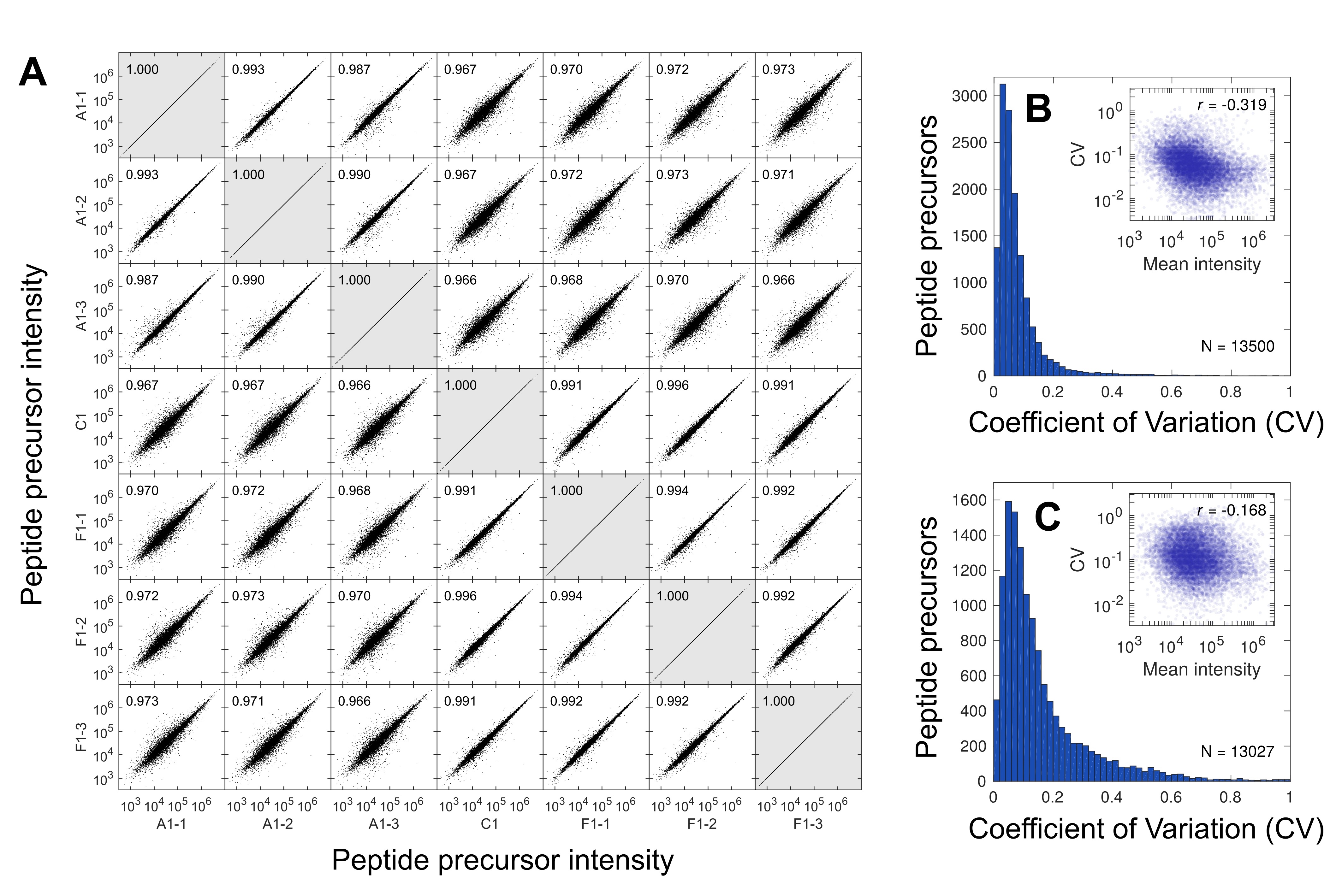
**

**Appendix Figure S1. Reproducibility of peptide precursors intensities.** **(A)** Scatter plot of peptide intensities among the control samples for *E. coli* MG1655 at hand. The A1, C1 and F1 samples are three biological replicates of the same condition (glucose minimal media; see Dataset EV5). The A1 and F1 samples have been measured in technical triplicates, which are identified as A1-1/2/3 and F1-1/2/3. In each panel the number indicate the Pearson correlations *r*, which is consistently above 0.987 for technical replicates and 0.966 for biological replicates. **(B)** Coefficients of variation (CV, standard deviation over mean) of peptide precursor intensities within three technical replicates (F1-1/2/3, on the left). Inset: scatter plot of CV and mean intensity of the peptides, in double-log scale. **(C)** Same as panel (B), but for three biological replicates (A1-1, C1, F1-1). The biological replicates are considerably more scattered than the technical replicates, as indicated by the larger median (0.108 vs. 0.0547) and by the longer tail of the distribution (90^th^ percentiles: 0.360 vs. 0.150). In both cases the CVs decrease with peptide abundance, with technical replicates showing a stronger dependence on intensity than biological replicates.


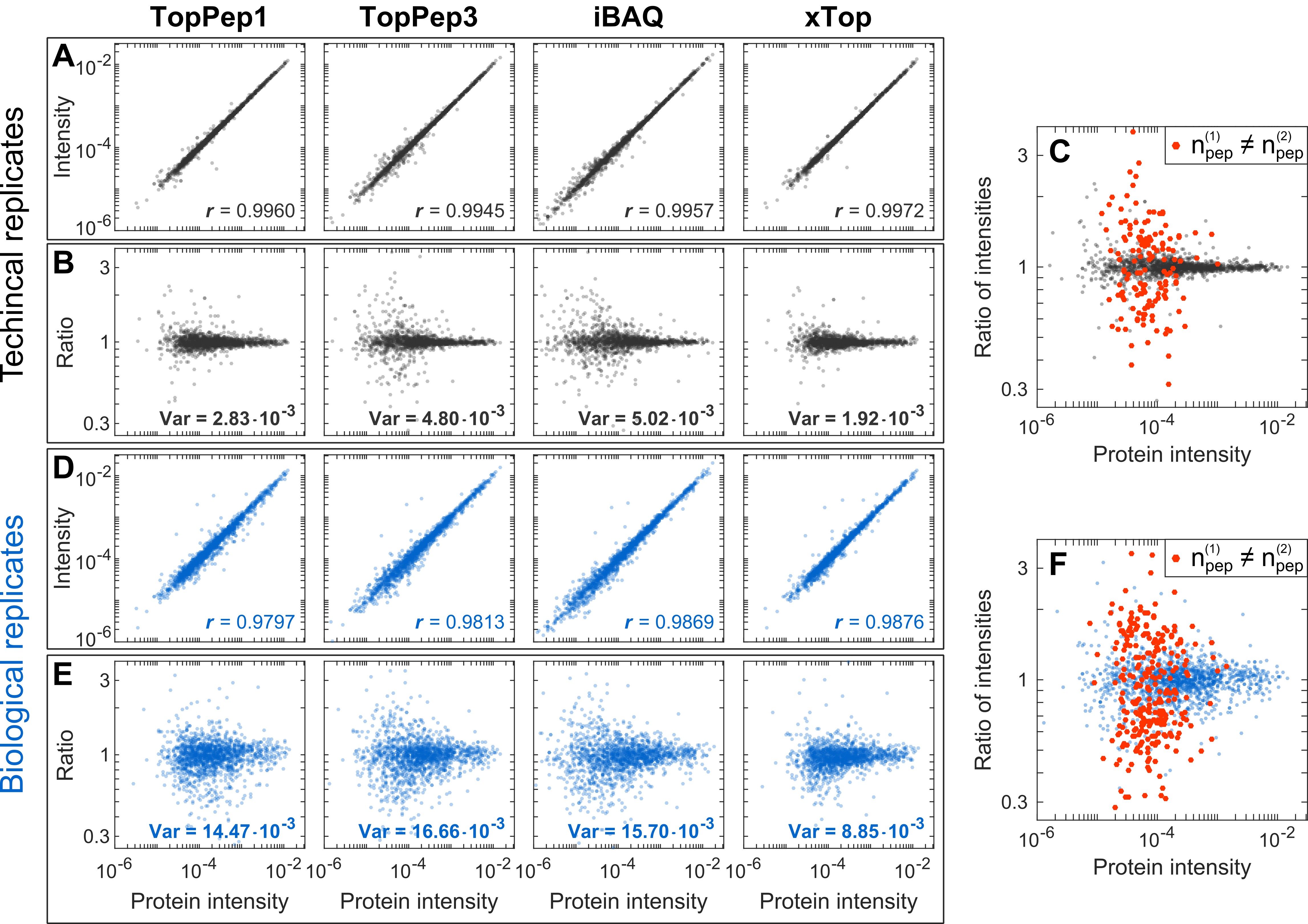


**Appendix Figure S2. Reproducibility of protein measurements for *E. coli* MG1655 in glucose minimal medium. (A)** Scatter plot of protein mass fraction for two representative technical (F1-2 versus F1-1). The protein intensities are computed with either TopPep1, TopPep3, iBAQ or xTop (left to right) and normalized to 1. The Pearson correlation coefficient ($r$) of the log-transformed intensities is shown in each panel. **(B)** Ratio of the protein intensities displayed in panel (A), plotted against the average protein intensity between the two replicates. The variance of the log-transformed ratios is shown in each panel. Despite similar correlation coefficients, TopPep3 and iBAQ display more scatter for low abundant proteins (approximately below 10^-4^). **(C)** For TopPep3, a large fraction of the scatter visible in panels (A) and (B) arises from proteins with unequal number of top peptide precursors $(1\leq n_{\mathrm{pep}}\leq3$) detected in the two samples (red points, 145 proteins), which occur due to missing peptide intensity values in one of the two samples that are compared to each other. **(D-F)** Same as the previous panels but using two biological replicates (A1-1 and F1-1); same data shown in Main Text Figure 2. The red points in panel (F) are 273, almost twice as many as in panel (C). The same *N=*1631 proteins which are detected by all methods in all conditions are shown in all panels.

**
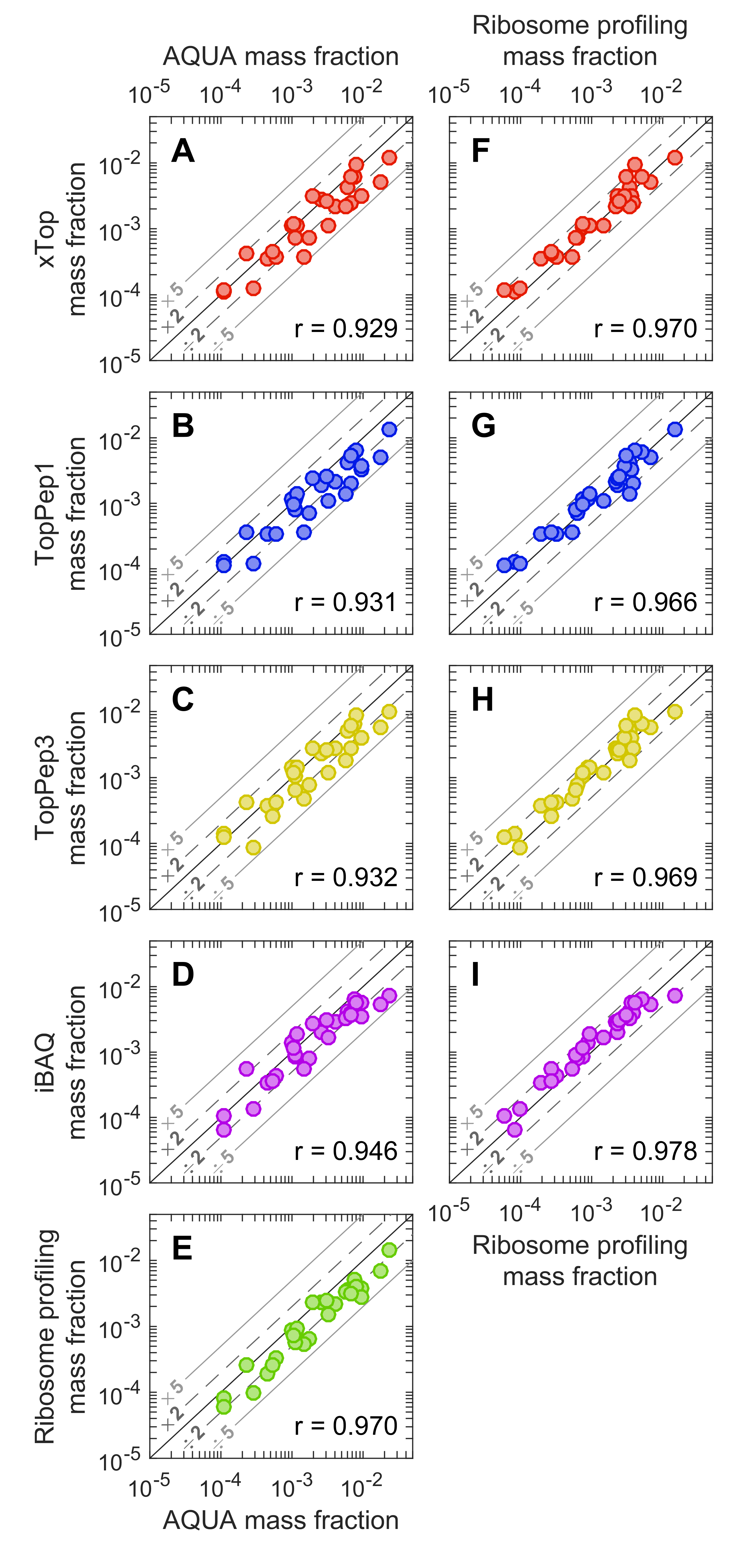
Appendix Figure S3. Absolute protein quantification with AQUA peptides.**

**(A-D)** Comparison of protein mass fractions obtained from four different protein quantification methods (from sample A1-1) and from ribosome profiling to mass fractions derived from stable isotope-labeled synthetic peptides (AQUA peptides) for a set of 29 anchor proteins (Dataset EV7). In each case, all data lies within 5-fold from the diagonal, with large (0.929 to 0.946) correlation coefficients (shown in each panel).

**(F)** Ribosome profiling-based mass fractions are also strongly correlated with the AQUA-derived mass fractions, with an even larger correlation coefficient ($r=0.970$) than for mass spec-based mass fractions.

**(F-I)** Comparison of mass spectrometry-derived protein mass fractions to mass fractions derived from ribosomal profiling data, for the same 29 proteins measured with AQUA. Correlations are even higher (0.966 to 0.978), and most data lie within 2-fold from the diagonal.


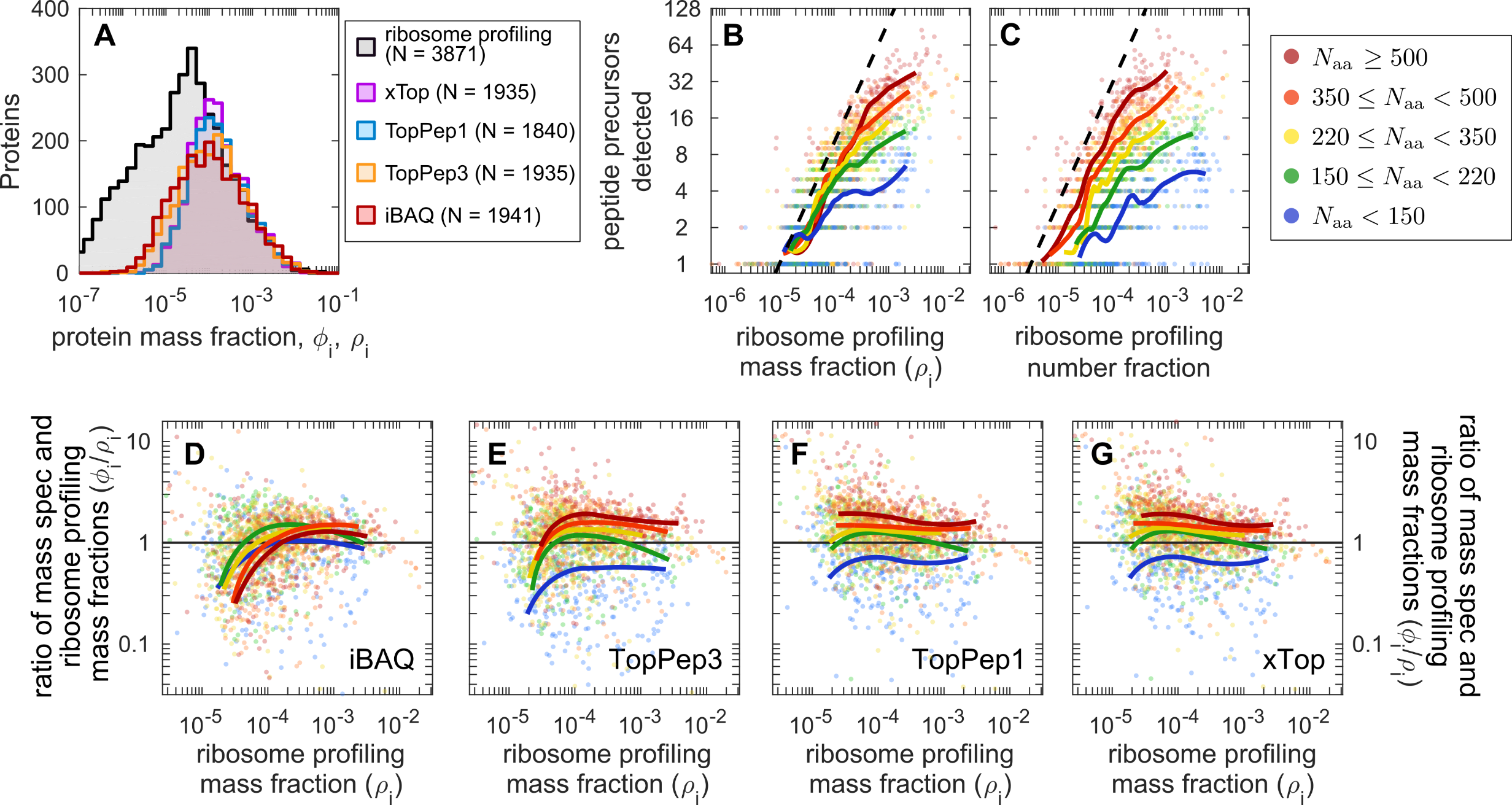


**Appendix Figure S4. Comparison of mass spectrometry based and ribosome profiling-based protein mass fraction. (A)** Histogram plotting the number of proteins detected in the calibration samples per mass fraction bin. In grey we show the distribution of mass fractions calculated from ribosome profiling reported using the data from Li *et al.* (Li *et al.*, 2014); the other colors represent the distribution of mass fractions determined in this work using proteomics and for different quantitative protein inference methods (xTop, TopPep1, TopPep3, iBAQ) in a representative calibration sample (F1-2). The distributions for the five approaches coincide for proteins with mass fractions above ${10}^{-4}$, suggesting that the vast majority of proteins above this mass fraction were quantified. At a mass fraction $\sim{10}^{-4}$, xTop and TopPep1 reported more proteins than TopPep3 and iBAQ; on the other hand iBAQ and TopPep3 reported more proteins below a mass fraction $3\cdot{10}^{-5}$ than xTop and TopPep1. Finally, ribosome profiling has a much deeper coverage than mass spec-based methods, allowing to capture proteins synthesized by the cell at very low rates (abundances below 1 protein per cell). (**B)** Number of peptide precursors detected as a function of ribosome profiling number fraction (number of protein synthesized per unit of time, normalized to the total number, see Appendix Note S2). The different colors indicate protein length windows (*L*); the solid lines represent moving averages for the proteins in each group. The number of peptide precursors increases with both the protein abundance (as reported by ribosome profiling, x-axis), and the protein size (increasing from blue to red). In particular, it vanishes at the same protein mass fraction ($\phi_{i}\sim{10}^{-5}$) for all protein size groups, and tapers off for abundant proteins at a value dependent on the size of the protein. The slope of the black dashed line corresponds to a linear relationship between peptide precursors detected and ribosome profiling signal. **(C)** Same as panel (B), but as a function of ribosome profiling number fractions, which are proportional to protein concentrations. In this case, the protein fraction at which the number of detected peptide precursors vanishes depends strongly on the protein size. **(D-G)** Ratio of mass spec- to ribosome profiling-derived protein mass fractions, $\phi_{i}/\rho_{i}$, versus the ribosome profiling-based mass fractions $\rho_{i}$. The solid lines are computed from running averages of $\phi_{i}$ and $\rho_{i}$ for proteins with sizes in the same five windows as in panels (B) and (C). A reduction in the ratio in $\phi_{i}/\rho_{i}$ for low abundant proteins ($\rho_{i}\lesssim{10}^{-4}$ is clearly visible for TopPep3 and iBAQ. On the other hand TopPep1/3 and xTop display a noticeable bias towards large proteins, i.e. tend to underestimate the signal of small proteins (blue) with respect to large ones (red).


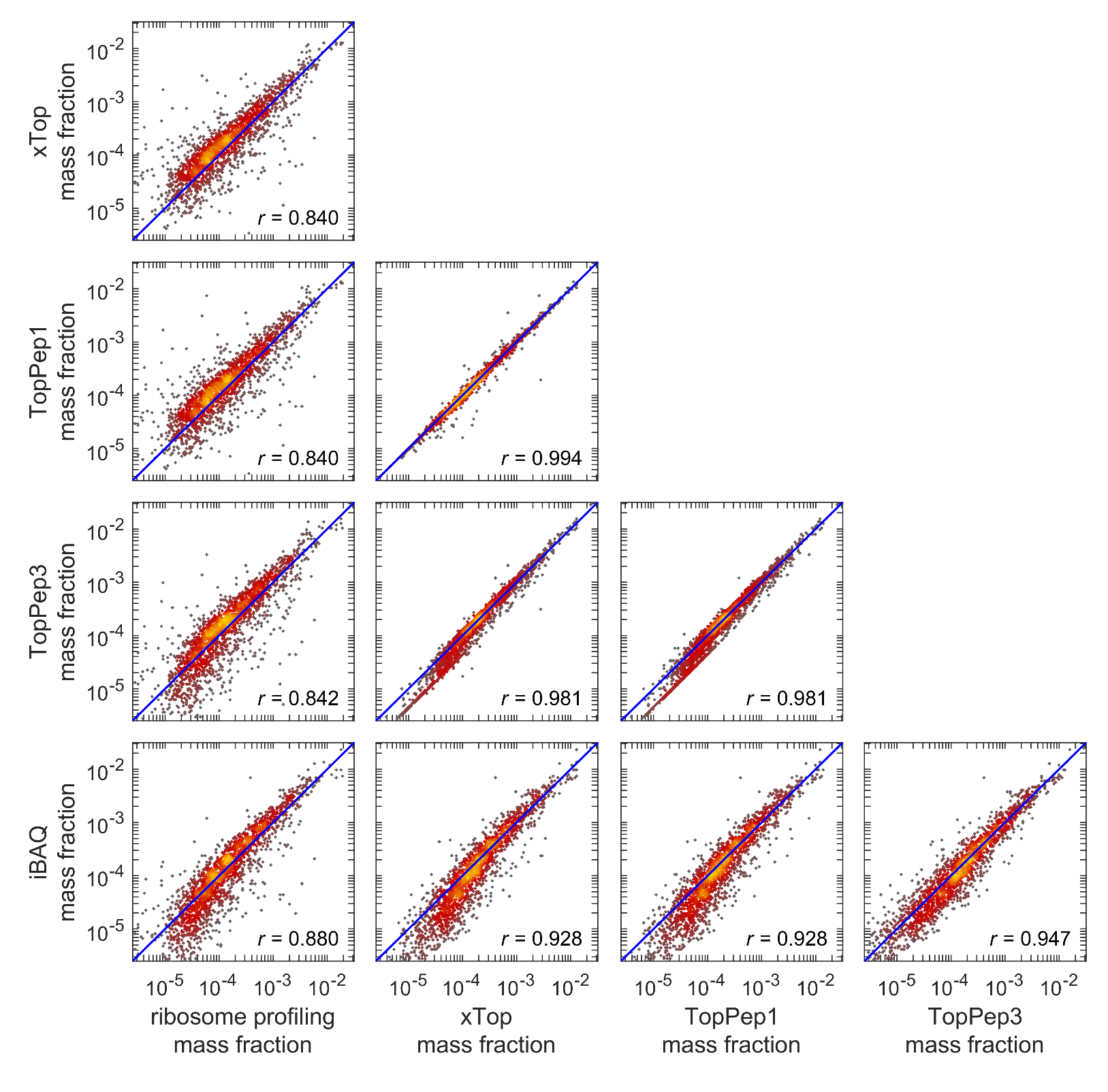


**Appendix Figure S5. Additional comparisons between mass spectrometry and ribosome profiling.** These scatter plots compare protein mass fractions from either ribosome profiling or the four different mass spectrometry-based protein quantification methods xTop, TopPep1, TopPep3 and iBAQ in a representative calibration sample (F1-2). The same proteins ($N=1823$) are shown in all panels. All mass spectrometry-based methods correlate strongly with ribosome profiling-based mass fractions ($r>0.84$); for TopPep3 and iBAQ a downward bend is visible for low-abundant proteins, as also shown perhaps more clearly in Appendix Figure S4D-E. When comparing different mass spectrometry-based quantification methods, we can make a number of observations. TopPep1 and xTop protein mass fractions are extremely tightly correlated; this was expected since the absolute scale of the xTop abundances is set to approximate that of TopPep1, but with reduced noise impact. TopPep3 mass fractions are well correlated with TopPep1 and xTop mass fractions, but in this case, a set of low abundant proteins can be seen forming a line roughly 2-fold below the diagonal. The reason behind this feature is that, for these proteins, only the top peptide precursor is detected, and thus TopPep3 and TopPep1 mass fractions are proportional to each other. Finally, the mass fractions computed via iBAQ show noticeable downward bends for low abundant proteins when comparing to either ribosome profiling, xTop or TopPep1.


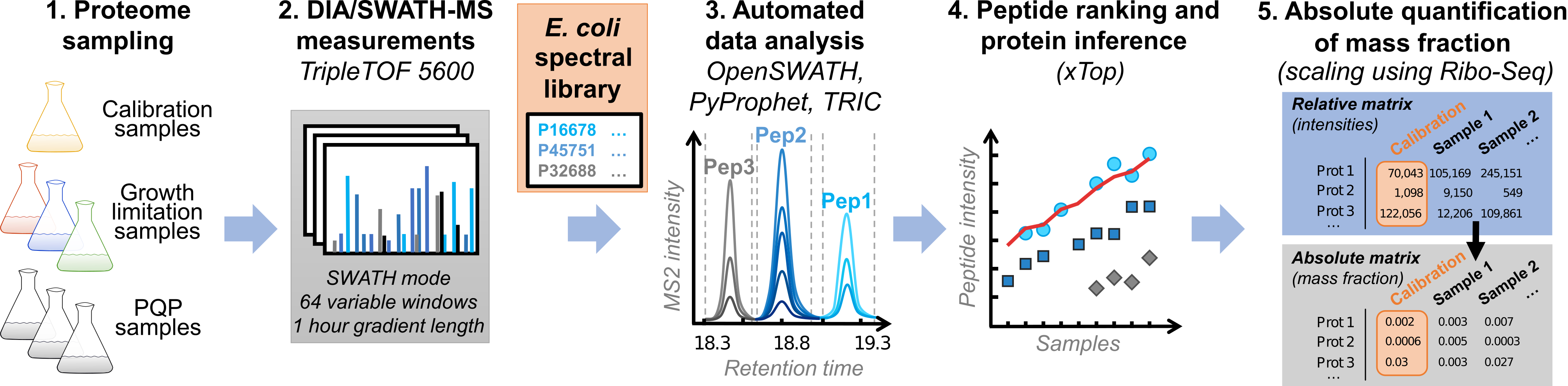
**Appendix Figure S6. Summary of the versatile workflow of DIA/SWATH protein quantification.** All *E. coli* full proteome samples were analyzed by DIA/SWATH in single shot measurements with a 60 min LC gradient and a 64 variable window data-independent acquisition scheme. The dataset was analysed in an automated fashion using OpenSWATH and our *E. coli* spectral library. The data was scored with OpenSWATH, validated with PyProphet and aligned in retention time with TRIC. Quantitative protein inference was performed using xTop. Absolute protein synthesis rates determined by Li *et al.* with ribosomal profiling (Li *et al.*, 2014) were used to convert xTop protein intensities through simple linear scaling into absolute protein mass fractions. The resulting absolute quantitative data on protein-level are provided in Datasets EV8 and EV9.


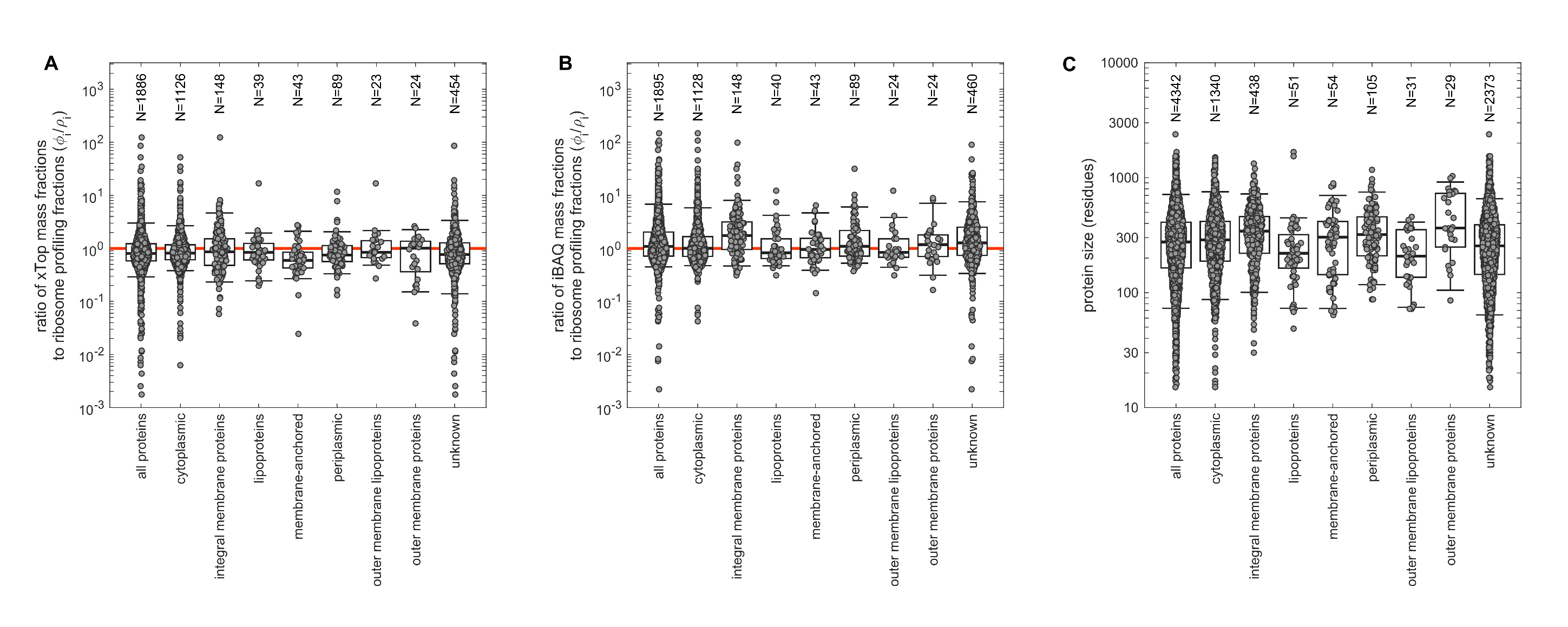


**Appendix Figure S7. Effect of protein localization on mass spec-based protein mass fractions. (A)** Ratio of xTop protein mass fractions to ribosome profiling-based mass fractions, $\phi_{i}/\rho_{i}$, for proteins with different cellular localization. All median ratios are close to unity, possibly with the exception of (the few) membrane anchored proteins, for which the median ratio is close to 0.6. **(B)** Same as panel (A), but for iBAQ protein intensities. In this case, the abundance of integral membrane proteins appears to be overestimated ~2-fold by iBAQ compared to ribosome profiling, while all other ratios are close to unity. **(C)** Protein size of proteins with different cellular localizations. While the average protein size is close to 300 residues, it is possible to observe patterns for different protein groups, e.g. integral membrane proteins tend to be larger than average, and lipoproteins tend to be smaller.


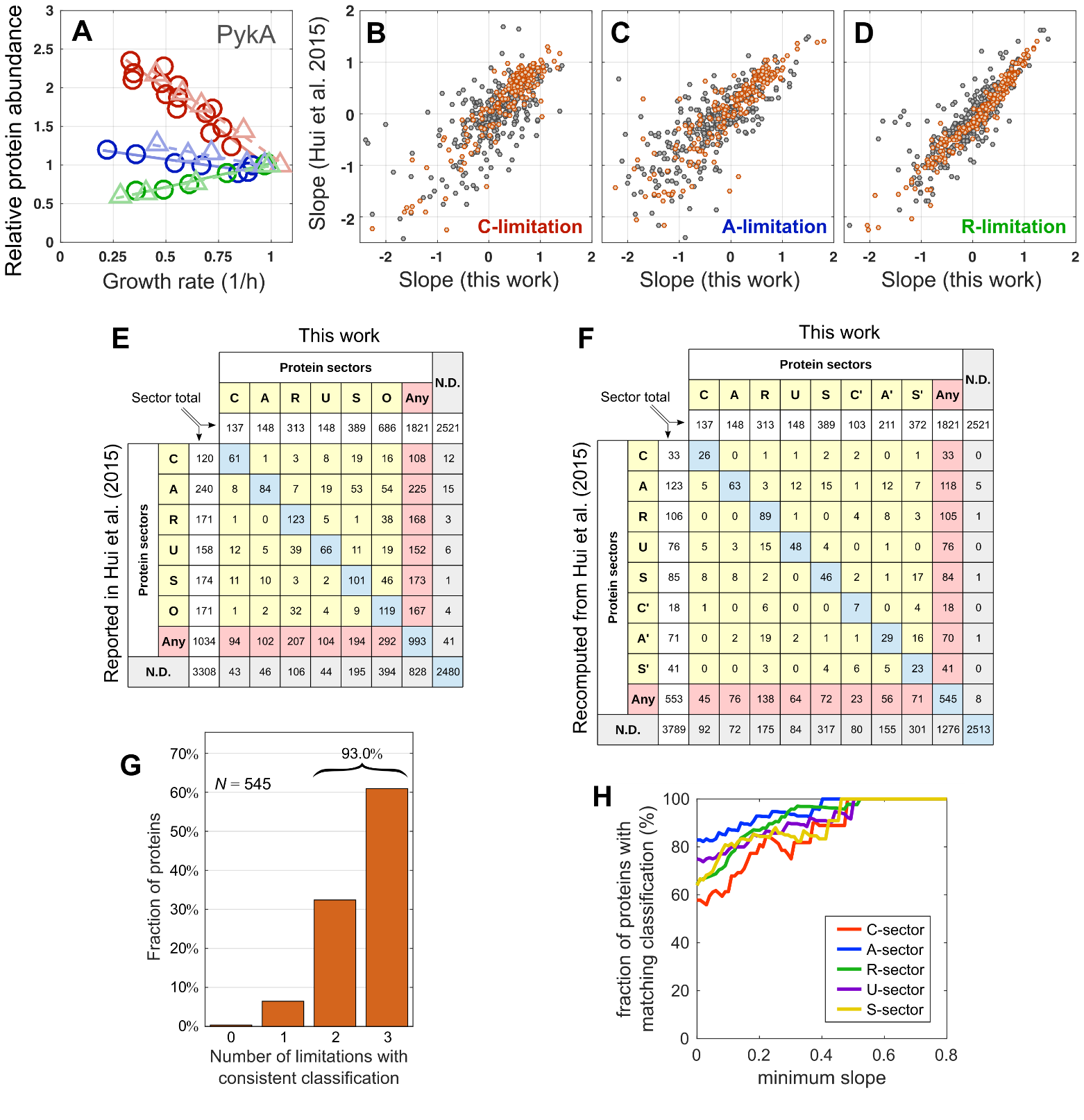


**Appendix Figure S8. Comparison with relative protein abundances from Hui *et al.* (2015)** (Hui *et al.*, 2015). **(A)** Relative protein abundance for the PykA (Pyruvate kinase A) protein against growth rate. Circles: this work. Triangles: Hui et al. (2015). The colors indicate the limitation series (C-, A- and R-limitation in red, blue and green, respectively). **(B-D)** For each of the three growth limitations (C-limitation, B; A-limitation, C; R-limitation, D), our absolute protein fractions were rescaled by their value $\phi_{i}^{\mathrm{ref}}$ in the reference condition (growth rate ~$0.9/h$). Then, these relative protein abundances $r_{i}={\phi_{i}}/{\phi_{i}^{\mathrm{ref}}}$and the ones from Hui et al. were fitted with a linear function of the growth rate as $r_{i}=r_{i,0}+s_{i}\lambda$. The fitted lines for the PykA protein are shown in panel (A) (Solid: this work; dashed, Hui et al.). The panels show how the fitted slopes compare to each other for 553 proteins that have been detected in all three limitations (with at least 3 detections per limitation series) in both datasets. Orange points represent proteins with a mass fraction in reference condition above ${10}^{-3}$ (197 proteins). **(E)** Sector membership comparison between the protein classification reported in Hui *et al*. and our work. Here, the C’-, A’- and S’-sectors have been merged together to allow comparison with the O-sector. Note that in Hui *et al*., the sector determination could be based on very sparse data (e.g. proteins only detected in C-limitations were assigned to the C-sector), while in this work we require at least three detections in each limitation. **(F)** Same as panel (E), except the protein membership was recomputed from the relative data in Hui *et al*. by using the same procedure as in our work. This reduces the number of protein with assigned sector compared to what done in Hui *et al*., but also improves the agreement between the two classifications by reducing the off-diagonal entries, especially for the A-sector membership from the Hui *et al*. dataset. **(G)** To summarize the consistency between the binary classifications from our work and from Hui et al. (2015), we computed for each protein the number of growth limitations in which the sign of the slope of protein abundance vs. the growth rate (panels A-D) is the same in both Hui dataset and ours. About 60% of the proteins are classified consistently in all three growth limitations, and thus belong to the same protein sector; more than 90% have at most one mismatch. **(H)** Proteins whose mass fractions have only a mild dependence on growth rate dependence cannot be confidently categorized into a single sector, and represent instead “borderline” cases. To quantify the impact of these mildly-varying proteins, we quantified the fraction of proteins with the same sector assignation based on our and Hui *et al.* datasets, only considering proteins whose slopes in the three limitations are at least larger than a threshold (shown on the x-axis). The panel shows the results for the five sectors with a well-defined growth rate response. The fraction of proteins with the same assignation starts above $\sim60\%$ with zero threshold, and increases towards 100% as the minimum slope threshold is increases, implying that the classification is more consistent when borderline proteins are removed.


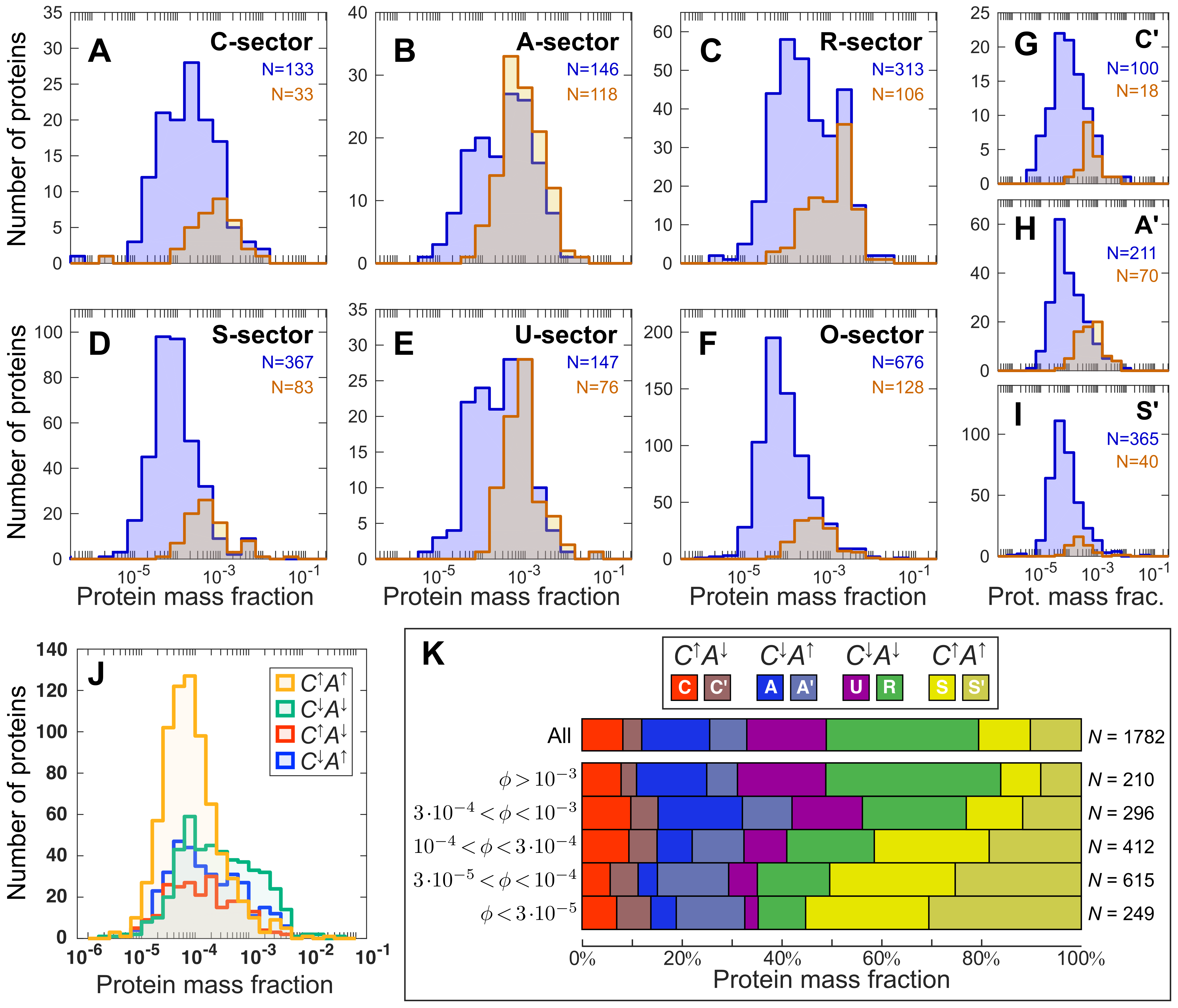


**Appendix Figure S9. Breakdown of protein abundances for the eight protein sectors defined Figure 4.** **(A-I)** For each protein sector, we show in blue the distribution of mass fractions in reference condition (glucose minimal medium) for the proteins in each sector (the number of proteins for each sector is shown in blue, summing up to 1782). For comparison, we also show the distributions for the proteins detected consistently (at least three samples per limitation series) in both our dataset and in the one from Hui *et al*., against our own absolute protein abundances (the number of proteins for each sectors is displayed in orange, summing up to 544). While the distributions are similar for mass fractions above ${10}^{-3}$, we were able to detect a large number of proteins with mass fractions between ${10}^{-5}$ and ${10}^{-4}$ which were not detected in the previous work. Panels (A-F) show the mass distributions for the six (C,A,R,U,S,O) sectors introduced in Hui *et al*. (2015), with the O-sector corresponding to the sum of C’-, A’- and S’-sectors (displayed in panels (G) to (I). **(J)** Distribution of proteins with different response in C- and A-limitation, as a function of protein abundance in reference condition. Proteins that are upregulated in both C-limitation and A-limitation ($C^{\uparrow}A^{\uparrow}$, belonging to either the S- or the S’-sector) are predominant among low abundant proteins (below $\phi\geq3\cdot{10}^{-4}$), while constituting only a small fraction of the high abundant proteins. Instead, the group of proteins that are downregulated in both limitations ($C^{\downarrow}A^{\downarrow}$, R- and U-sectors) can be characterized as a set of few, but highly abundant, proteins. **(K)** Breakdown of the proteome composition of *E. coli* in glucose minimal medium into the eight protein sectors, for different ranges of protein abundances. The first row (“All”) shows the mass fraction of the eight sectors in reference condition (glucose minimal medium). This is broken down below into bins corresponding to different protein abundances. The overall composition of the proteome into sectors only reflects the regulation of the most abundant proteins ($\phi\geq3\cdot{10}^{-4}$), while the regulation of low abundant proteins ($\phi<{3\cdot10}^{-4}$) is qualitatively different, as more than half of the proteins are upregulated in both C- and A-limitation (S- and S’-sectors).


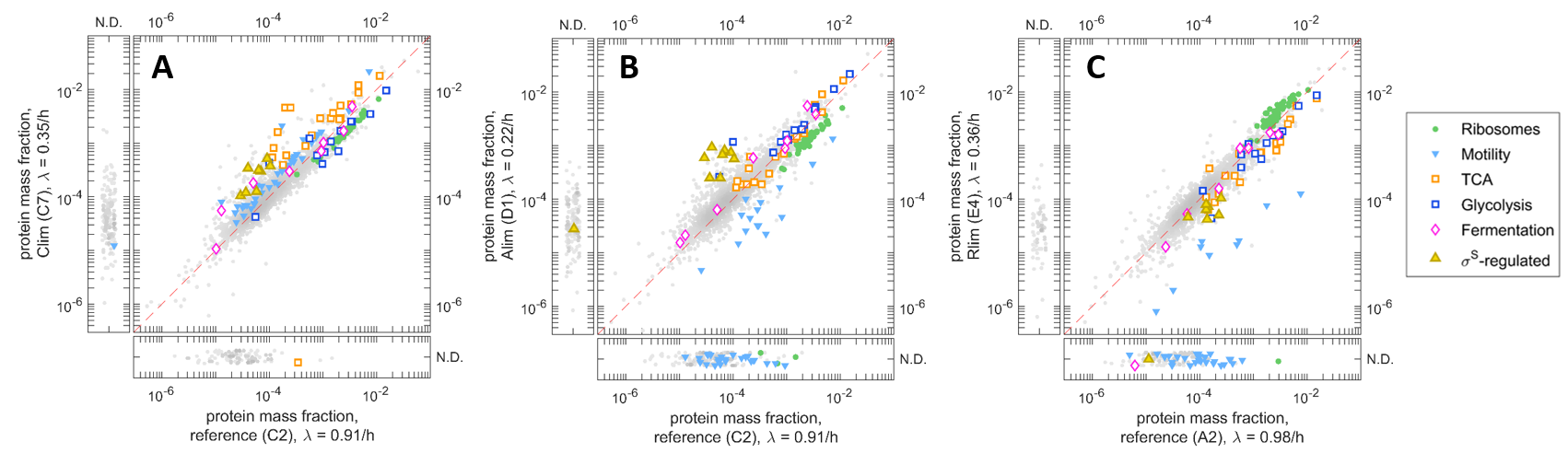
**Appendix Figure S10. Scatter plot of protein mass fractions in extreme growth limitation versus reference condition.** Each of these plots compares the absolute protein mass fractions between a pair of samples; the axis labels include the sample ID and the corresponding growth rate. The side boxes include proteins that have been detected only on one of the two samples. Some protein functional groups are highlighted with colored symbols: ribosomal proteins (*rpl*, *rpm* and *rps* gene groups, green circles), motility proteins (*che*, *flg* and *fli* gene groups, plus *aer*, *motAB*, *tap*, *tar*, *trg* and *tsr* genes, downward cyan triangle), TCA proteins (*aceAB, acnAB, acs, fumAC, glcB, gltA, icd, mdh, mqo, sdhABCD* and *sucABCD* genes, open orange squares), glycolytic proteins (*aceEF, eno, fbaAB, gapA, gpmAM, pfkAB, pgi, pgk, pykAF* and *tpiA* genes, open blue squares), fermentation enzymes (*ackA, adhE, fdhF, frd, hyc, ldhA, pflB* and *pta* genes, pink diamonds) and a set of $\sigma^{S}$-driven genes (*dps*, *ecnB*, *elaB*, *katE*, *osmCE*, *otsAB* and *wrbA* genes, open yellow triangles). **(A)** Slow C-limited growth (titrating glucose uptake) versus reference condition (NCM3722 in glucose minimal medium). **(B)** Slow A-limited growth (titrating ammonia assimilation) versus reference condition. **(C)** Slow R-limited growth (sublethal doses of the translation limiting chloramphenicol) versus reference condition.


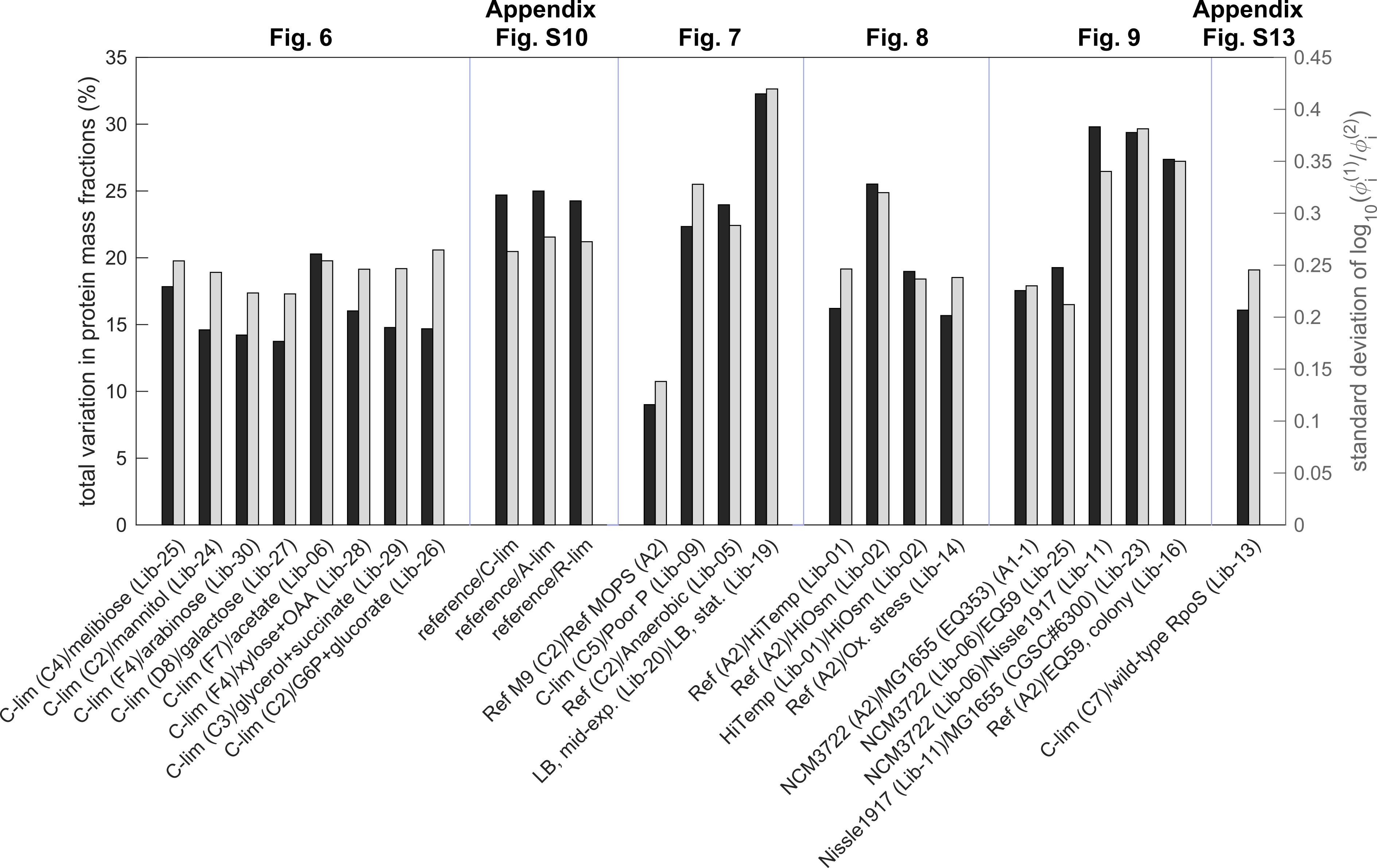


**Appendix Figure S11. Total change in proteome composition between pairs of samples.** The plot includes two distinct aggregate measures of proteome similarity for various pairwise comparisons described in the text. The total variation in protein mass fractions (black bars, left axis) was obtained by computing for each protein $i$ the differences in mass fractions between the two samples, $\Delta\phi_{i}=\phi_{i}^{(1)}-\phi_{i}^{(2)}$, and then summing all positive differences to obtain the overall change in proteome allocation. An additional measure of proteome similarity is given by the standard deviation of the log-transformed protein ratios (light grey, right axis), which more directly corresponds to the overall spread visible in scatter plots between pairs of samples. To do so, we computed for each pair of samples the log-transformed ratios of protein mass fractions, $\log_{10} \phi_{i}^{(1)}/\phi_{i}^{(2)}$ for protein $i$, and then the standard deviation of their logarithm (light grey bars, right axis). This measure provides a measure of the overall spread visible in scatter plots of the two protein mass fractions.

**
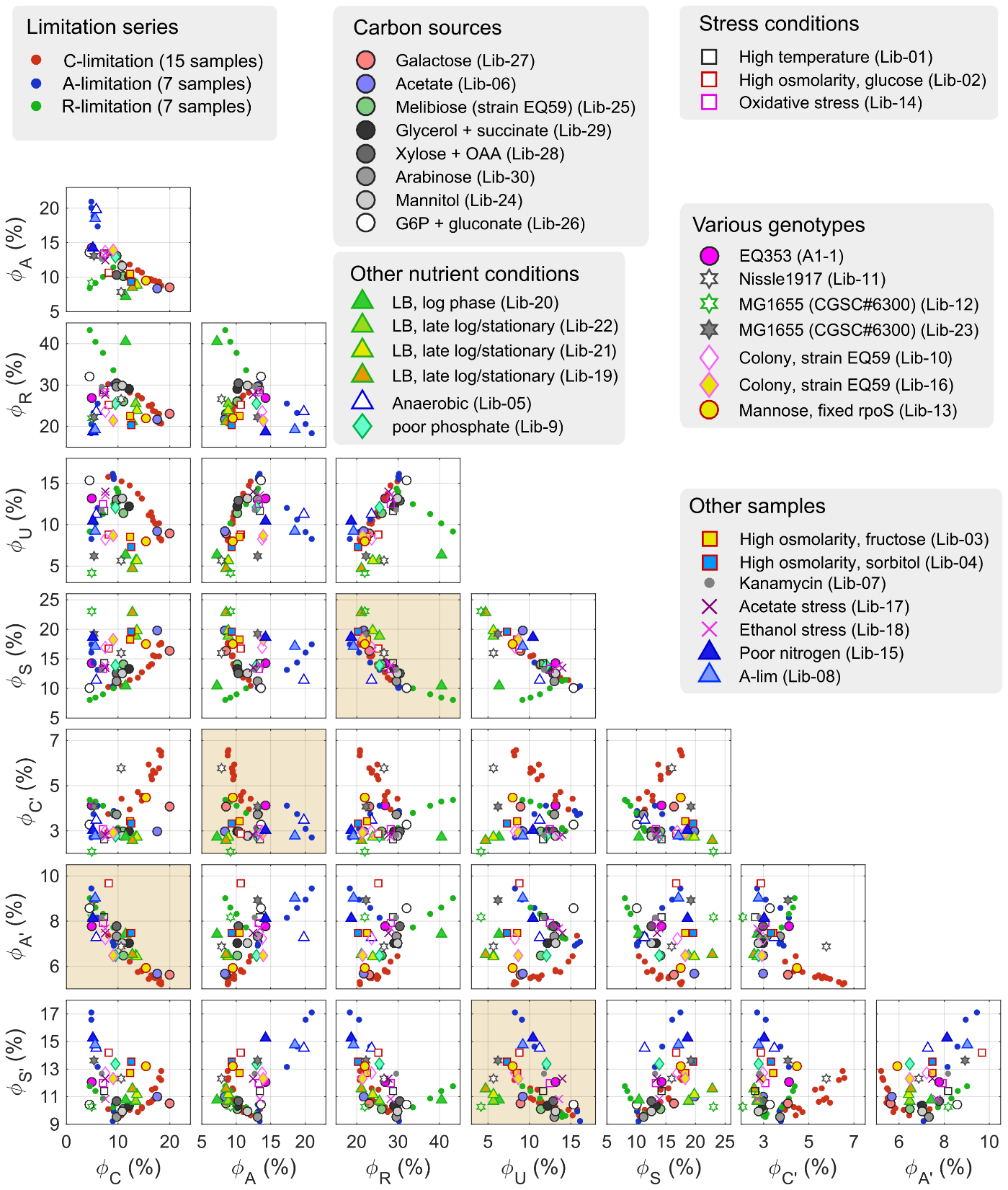
**

**Appendix Figure S12. Correlation between the eight protein sectors across various conditions.** The eight protein sectors described in Fig. 4 are correlated against each other. Panels with dark background represent complementary sectors, e.g. the S-sector ($C^{\uparrow}A^{\uparrow}R^{\downarrow}$) against the R-sector ($C^{\downarrow}A^{\downarrow}R^{\uparrow}$), for which the protein mass fractions anti-correlate in each of the three growth limitations.


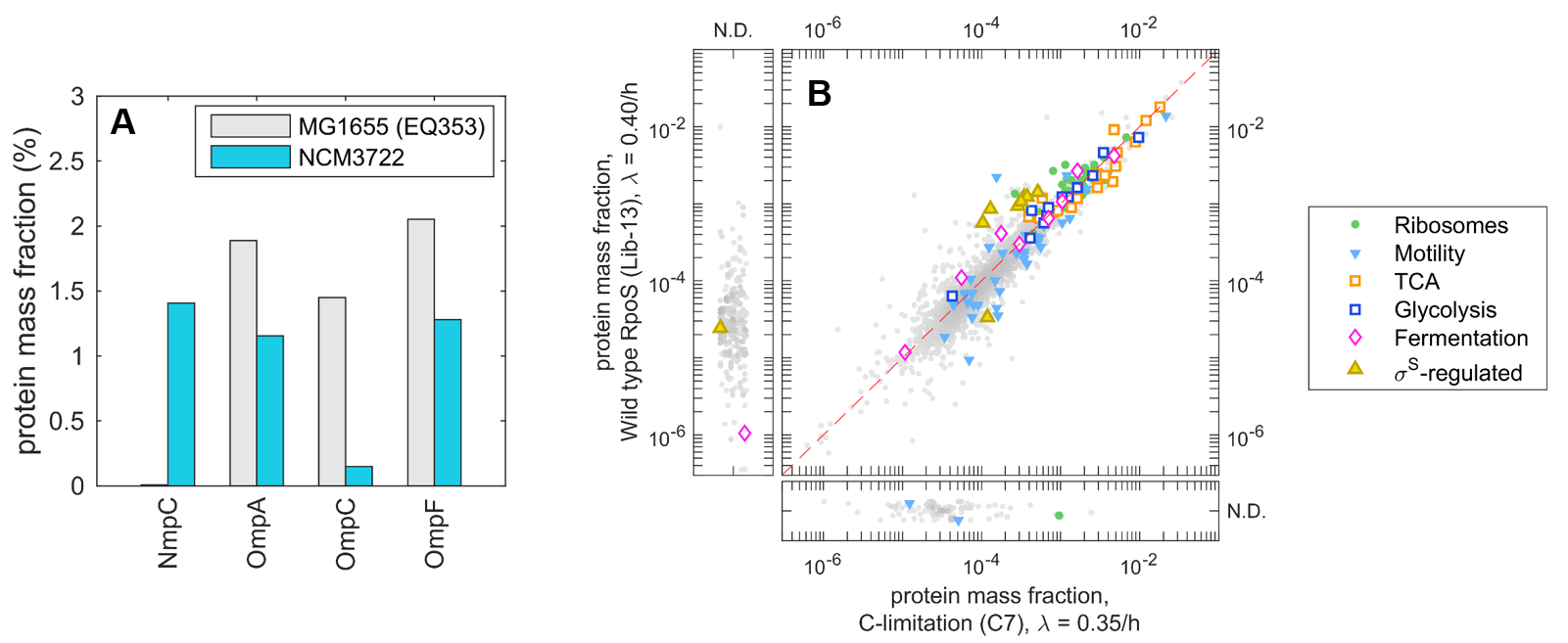


**Appendix Figure S13. Expression of porins and** $\boldsymbol{\sigma}^{\boldsymbol{S}}$**-dependent genes. (A)** Abundance of the four major outer membrane porins in E. coli MG1655 (EQ353) and NCM3722. The *nmpC* gene is nonfunctional in MG1655 due to the presence of an insertion element. The lack of NmpC is apparently compensated by a greatly increased expression of OmpC. **(B)** To understand the mild upregulation of $\sigma^{S}$-dependent genes in MG1655 (EQ353), we compared the protein mass fraction between strain NQ1527 (restored *rpoS* expression, removing amber codon mutation present in NCM3722; see Extended experimental methods) in mannose minimal medium, and slow carbon-limited growth (NCM3722-derived strain). Protein expression is similar in the two cases, with only a mild upregulation of $\sigma^{S}$-dependent genes (yellow triangles), similar to what is seen for MG1655.


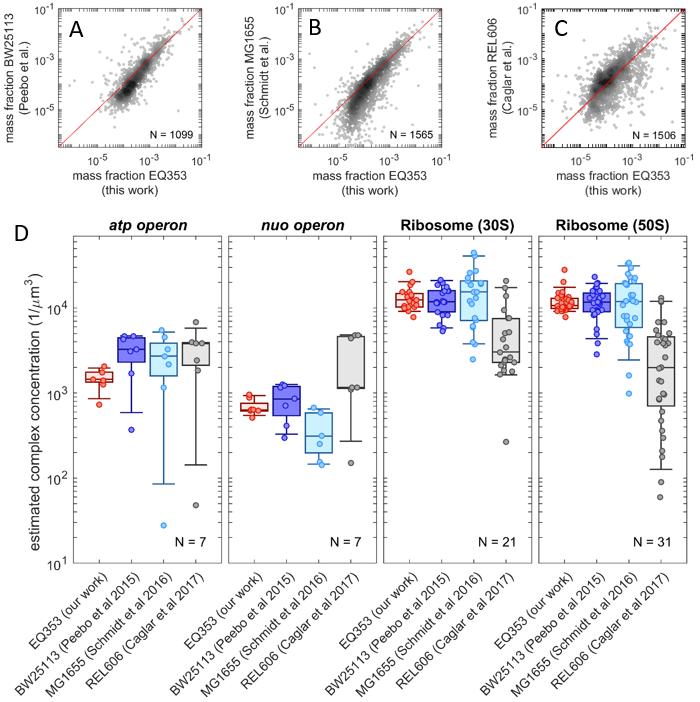


**Appendix Figure S14. Comparison with other proteomics works. (A)** Scatter plot of protein mass fractions from Peebo et al. (Peebo *et al*, 2015) for *E. coli* BW25113 in glucose minimal medium to MG1655 (EQ353) (sample A1-1). **(B)** Scatter plot of protein mass fractions from Schmidt et al. (Schmidt *et al*, 2016a) for *E. coli* MG1655 in glucose minimal medium compared to our data for MG1655 (EQ353) in the same condition. **(C)** Scatter plot of protein mass fractions from Caglar et al. (Caglar *et al*, 2017) for exponentially growing *E. coli* B REL606 in glucose minimal medium compared to our data for MG1655 (EQ353). **(D)** Estimated protein complex concentration for four different complexes (ATP synthase complex, NADH dehydrogenase, small ribosome subunit and large ribosome subunit). The figure is similar to Fig. 3B-E, except here we compare the protein abundances from our work, Peebo et al., Schmidt et al. and Caglar et al. Only proteins detected in all four datasets were considered for the comparison.

# Datasets

**Datasets EV1-EV12. Full proteomics and ribosome sequencing dataset of this study.** The *xlsx* files available in the online version of this work provide the full *E. coli* proteomics and ribosome sequencing dataset generated in this study, including description of the strains and of the growth conditions, peptide-level intensities, absolute protein mass fractions, ribosome sequencing synthesis rates, as well as the results of the downstream analysis (definition of protein sectors and the corresponding GO-term enrichment analysis).

# References

Baba T, Ara T, Hasegawa M, Takai Y, Okumura Y, Baba M, Datsenko KA, Tomita M, Wanner BL, Mori H (2006) Construction of Escherichia coli K-12 in-frame, single-gene knockout mutants: the Keio collection. *Molecular systems biology* 2: 2006.0008

Basan M, Hui S, Okano H, Zhang Z, Shen Y, Williamson JR, Hwa T (2015a) Overflow metabolism in Escherichia coli results from efficient proteome allocation. *Nature* 528: 99-104

Basan M, Zhu M, Dai X, Warren M, Sevin D, Wang YP, Hwa T (2015b) Inflating bacterial cells by increased protein synthesis. *Molecular systems biology* 11: 836

Brown SD, Jun S (2015) Complete Genome Sequence of Escherichia coli NCM3722. *Genome announcements* 3

Caglar MU, Houser JR, Barnhart CS, Boutz DR, Carroll SM, Dasgupta A, Lenoir WF, Smith BL, Sridhara V, Sydykova DK *et al* (2017) The E. coli molecular phenotype under different growth conditions. *Scientific reports* 7: 45303

Chen CM, Ye QZ, Zhu ZM, Wanner BL, Walsh CT (1990) Molecular biology of carbon-phosphorus bond cleavage. Cloning and sequencing of the phn (psiD) genes involved in alkylphosphonate uptake and C-P lyase activity in Escherichia coli B. *The Journal of biological chemistry* 265: 4461-4471

Choi H, Nesvizhskii AI (2008) Semisupervised model-based validation of peptide identifications in mass spectrometry-based proteomics. *Journal of proteome research* 7: 254-265

Collins BC, Hunter CL, Liu Y, Schilling B, Rosenberger G, Bader SL, Chan DW, Gibson BW, Gingras AC, Held JM *et al* (2017) Multi-laboratory assessment of reproducibility, qualitative and quantitative performance of SWATH-mass spectrometry. *Nature communications* 8: 291

Craig R, Beavis RC (2003) A method for reducing the time required to match protein sequences with tandem mass spectra. *Rapid communications in mass spectrometry : RCM* 17: 2310-2316

Csonka LN, Ikeda TP, Fletcher SA, Kustu S (1994) The accumulation of glutamate is necessary for optimal growth of Salmonella typhimurium in media of high osmolality but not induction of the proU operon. *Journal of bacteriology* 176: 6324-6333

Deutsch EW, Chambers M, Neumann S, Levander F, Binz P-A, Shofstahl J, Campbell DS, Mendoza L, Ovelleiro D, Helsens K *et al* (2012) TraML—A Standard Format for Exchange of Selected Reaction Monitoring Transition Lists. *Molecular & cellular proteomics : MCP* 11: R111.015040

Deutsch EW, Mendoza L, Shteynberg D, Farrah T, Lam H, Tasman N, Sun Z, Nilsson E, Pratt B, Prazen B *et al* (2010) A guided tour of the Trans-Proteomic Pipeline. *Proteomics* 10: 1150-1159

Eng JK, Jahan TA, Hoopmann MR (2013) Comet: an open-source MS/MS sequence database search tool. *Proteomics* 13: 22-24

Escher C, Reiter L, MacLean B, Ossola R, Herzog F, Chilton J, MacCoss MJ, Rinner O (2012) Using iRT, a normalized retention time for more targeted measurement of peptides. *Proteomics* 12: 1111-1121

Geer LY, Markey SP, Kowalak JA, Wagner L, Xu M, Maynard DM, Yang X, Shi W, Bryant SH (2004) Open mass spectrometry search algorithm. *Journal of proteome research* 3: 958-964

Gerber SA, Rush J, Stemman O, Kirschner MW, Gygi SP (2003) Absolute quantification of proteins and phosphoproteins from cell lysates by tandem MS. *Proceedings of the National Academy of Sciences of the United States of America* 100: 6940-6945

Gutnick D, Calvo JM, Klopotowski T, Ames BN (1969) Compounds which serve as the sole source of carbon or nitrogen for Salmonella typhimurium LT-2. *Journal of bacteriology* 100: 215-219

Hui S, Silverman JM, Chen SS, Erickson DW, Basan M, Wang J, Hwa T, Williamson JR (2015) Quantitative proteomic analysis reveals a simple strategy of global resource allocation in bacteria. *Molecular systems biology* 11: 784

Keseler IM, Mackie A, Santos-Zavaleta A, Billington R, Bonavides-Martínez C, Caspi R, Fulcher C, Gama-Castro S, Kothari A, Krummenacker M *et al* (2017) The EcoCyc database: reflecting new knowledge about Escherichia coli K-12. *Nucleic Acids Res* 45: D543-d550

Koch AL, Levy HR (1955) Protein turnover in growing cultures of Escherichia coli. *The Journal of biological chemistry* 217: 947-957

Kochanowski K, Volkmer B, Gerosa L, Haverkorn van Rijsewijk BR, Schmidt A, Heinemann M (2013) Functioning of a metabolic flux sensor in Escherichia coli. *Proceedings of the National Academy of Sciences of the United States of America* 110: 1130-1135

Lam H, Deutsch EW, Eddes JS, Eng JK, King N, Stein SE, Aebersold R (2007) Development and validation of a spectral library searching method for peptide identification from MS/MS. *Proteomics* 7: 655-667

Lam H, Deutsch EW, Eddes JS, Eng JK, Stein SE, Aebersold R (2008) Building consensus spectral libraries for peptide identification in proteomics. *Nature methods* 5: 873-875

Langmead B, Trapnell C, Pop M, Salzberg SL (2009) Ultrafast and memory-efficient alignment of short DNA sequences to the human genome. *Genome biology* 10: R25

Li GW, Burkhardt D, Gross C, Weissman JS (2014) Quantifying absolute protein synthesis rates reveals principles underlying allocation of cellular resources. *Cell* 157: 624-635

Li GW, Oh E, Weissman JS (2012) The anti-Shine-Dalgarno sequence drives translational pausing and codon choice in bacteria. *Nature* 484: 538-541

Lyons E, Freeling M, Kustu S, Inwood W (2011a) Using genomic sequencing for classical genetics in E. coli K12. *PloS one* 6: e16717

Lyons E, Freeling M, Kustu S, Inwood W (2011b) Using genomic sequencing for classical genetics in E. coli K12. *PloS one* 6: e16717-e16717

MacLean B, Eng JK, Beavis RC, McIntosh M (2006) General framework for developing and evaluating database scoring algorithms using the TANDEM search engine. *Bioinformatics (Oxford, England)* 22: 2830-2832

MacLean B, Tomazela DM, Shulman N, Chambers M, Finney GL, Frewen B, Kern R, Tabb DL, Liebler DC, MacCoss MJ (2010) Skyline: an open source document editor for creating and analyzing targeted proteomics experiments. *Bioinformatics (Oxford, England)* 26: 966-968

Makino K, Kim SK, Shinagawa H, Amemura M, Nakata A (1991) Molecular analysis of the cryptic and functional phn operons for phosphonate use in Escherichia coli K-12. *Journal of bacteriology* 173: 2665-2672

Malmstrom J, Lee H, Nesvizhskii AI, Shteynberg D, Mohanty S, Brunner E, Ye M, Weber G, Eckerskorn C, Aebersold R (2006) Optimized peptide separation and identification for mass spectrometry based proteomics via free-flow electrophoresis. *Journal of proteome research* 5: 2241-2249

Mori M, Schink S, Erickson DW, Gerland U, Hwa T (2017) Quantifying the benefit of a proteome reserve in fluctuating environments. *Nature communications* 8: 1225

Neidhardt FC, Bloch PL, Smith DF (1974) Culture medium for enterobacteria. *Journal of bacteriology* 119: 736-747

Peebo K, Valgepea K, Maser A, Nahku R, Adamberg K, Vilu R (2015) Proteome reallocation in Escherichia coli with increasing specific growth rate. *Molecular bioSystems* 11: 1184-1193

Picotti P, Bodenmiller B, Mueller LN, Domon B, Aebersold R (2009) Full dynamic range proteome analysis of S. cerevisiae by targeted proteomics. *Cell* 138: 795-806

Reiter L, Claassen M, Schrimpf SP, Jovanovic M, Schmidt A, Buhmann JM, Hengartner MO, Aebersold R (2009) Protein identification false discovery rates for very large proteomics data sets generated by tandem mass spectrometry. *Molecular & cellular proteomics : MCP* 8: 2405-2417

Rosenberger G, Bludau I, Schmitt U, Heusel M, Hunter CL, Liu Y, MacCoss MJ, MacLean BX, Nesvizhskii AI, Pedrioli PGA *et al* (2017a) Statistical control of peptide and protein error rates in large-scale targeted data-independent acquisition analyses. *Nature methods* 14: 921-927

Rosenberger G, Liu Y, Rost HL, Ludwig C, Buil A, Bensimon A, Soste M, Spector TD, Dermitzakis ET, Collins BC *et al* (2017b) Inference and quantification of peptidoforms in large sample cohorts by SWATH-MS. *Nature biotechnology* 35: 781-788

Rost HL, Liu Y, D'Agostino G, Zanella M, Navarro P, Rosenberger G, Collins BC, Gillet L, Testa G, Malmstrom L *et al* (2016) TRIC: an automated alignment strategy for reproducible protein quantification in targeted proteomics. *Nature methods* 13: 777-783

Rost HL, Rosenberger G, Navarro P, Gillet L, Miladinovic SM, Schubert OT, Wolski W, Collins BC, Malmstrom J, Malmstrom L *et al* (2014) OpenSWATH enables automated, targeted analysis of data-independent acquisition MS data. *Nature biotechnology* 32: 219-223

Schmidt A, Kochanowski K, Vedelaar S, Ahrne E, Volkmer B, Callipo L, Knoops K, Bauer M, Aebersold R, Heinemann M (2016a) The quantitative and condition-dependent Escherichia coli proteome. *Nature biotechnology* 34: 104-110

Schmidt A, Kochanowski K, Vedelaar S, Ahrné E, Volkmer B, Callipo L, Knoops K, Bauer M, Aebersold R, Heinemann M (2016b) The quantitative and condition-dependent Escherichia coli proteome. *Nature biotechnology* 34: 104-110

Schubert OT, Gillet LC, Collins BC, Navarro P, Rosenberger G, Wolski WE, Lam H, Amodei D, Mallick P, MacLean B *et al* (2015) Building high-quality assay libraries for targeted analysis of SWATH MS data. *Nature protocols* 10: 426-441

Sharma V, Eckels J, Schilling B, Ludwig C, Jaffe JD, MacCoss MJ, MacLean B (2018) Panorama Public: A Public Repository for Quantitative Data Sets Processed in Skyline. *Molecular & cellular proteomics : MCP* 17: 1239-1244

Shteynberg D, Deutsch EW, Lam H, Eng JK, Sun Z, Tasman N, Mendoza L, Moritz RL, Aebersold R, Nesvizhskii AI (2011) iProphet: multi-level integrative analysis of shotgun proteomic data improves peptide and protein identification rates and error estimates. *Molecular & cellular proteomics : MCP* 10: M111.007690

Sonnenborn U, Schulze J (2009) The non-pathogenic Escherichia coli strain Nissle 1917 – features of a versatile probiotic. *Microbial Ecology in Health and Disease* 21: 122-158

Tabb DL, Fernando CG, Chambers MC (2007) MyriMatch: highly accurate tandem mass spectral peptide identification by multivariate hypergeometric analysis. *Journal of proteome research* 6: 654-661

Teleman J, Rost HL, Rosenberger G, Schmitt U, Malmstrom L, Malmstrom J, Levander F (2015) DIANA--algorithmic improvements for analysis of data-independent acquisition MS data. *Bioinformatics (Oxford, England)* 31: 555-562

Warren MR, Sun H, Yan Y, Cremer J, Li B, Hwa T (2019) Spatiotemporal establishment of dense bacterial colonies growing on hard agar. *eLife* 8

You C, Okano H, Hui S, Zhang Z, Kim M, Gunderson CW, Wang Y-P, Lenz P, Yan D, Hwa T (2013) Coordination of bacterial proteome with metabolism by cyclic AMP signalling. *Nature* 500: 301-306
